# Polar questions in Dutch Sign Language (NGT): A production experiment

**DOI:** 10.1371/journal.pone.0354015

**Published:** 2026-07-29

**Authors:** Marloes Oomen, Lyke Esselink, Floris Roelofsen

**Affiliations:** 1 Amsterdam Center for Language and Communication, University of Amsterdam, Amsterdam, The Netherlands; 2 Institute for Logic, Language and Computation, University of Amsterdam, Amsterdam, The Netherlands; Lamar University, UNITED STATES OF AMERICA

## Abstract

Polar questions play a fundamental role in communication and have been investigated across a wide range of spoken languages, from which we know that polar question forms may vary considerably across varying contexts. Much less is known about polar question forms in different contexts in sign languages. This paper concentrates on one particular sign language, namely Dutch Sign Language (NGT). It investigates (i) which polar question forms exist in NGT, (ii) in which types of context these different forms are used, and (iii) what the semantic contribution is of specific elements that may occur in polar questions. We present a production experiment in which two contextual factors are manipulated through role play between the participant and two confederates: (i) the prior expectations of the person asking the question with respect to the truth of the proposition that the question is about, and (ii) the evidence available in the immediate context of utterance with respect to the truth of this proposition. We identify a broad range of polar question forms, differing in terms of *polarity marking* (positive, negative, null), the presence or absence of *question tags*, and use of *non-manual markers*. We observe that each question form has a distinct distribution across the different contexts of use under consideration. With regard to non-manuals, a notable finding is that, contrary to expectation given the previous literature on polar questions in sign languages, raised eyebrows are not consistently used in the polar questions in our dataset; in fact, brow lowering is also rather common. This leads us to suggest that a head or body forward position – not raised eyebrows – is the main polar question marker in NGT. We suggest that raised and lowered eyebrows mark a broad range of other functions that may be relevant in (non-canonical) polar questions.

## Introduction

Polar questions are questions which inquire about the truth of some proposition. The sentence in (1) is an example of a polar question in English, inquiring about the truth of the proposition that *whales are mammals*. It involves subject-auxiliary inversion and typically rising intonation toward the end of the sentence, which are usually seen as characteristic features of ‘canonical’ or ‘prototypical’ polar questions in English.

(1) Are whales mammals?

Polar questions play a fundamental role in communication and are attested in all of the 955 languages surveyed in the World Atlas of Language Structures (WALS; [1]), as well as all of the 76 languages surveyed in the World Atlas of Pidgin and Creole Language Structures (APiCS; [2]). As a consequence, polar questions have been studied extensively. Much progress has been made in (i) identifying the different **forms** that polar questions can have across languages (in terms of syntactic structure, the use of particles, and prosody), (ii) identifying the **contexts of use** for each of these polar question forms (in particular in terms of prior speaker belief and contextual evidence with respect to the truth of the proposition in question), and (iii) identifying the **semantic contribution** of specific elements that may occur in polar questions (e.g., particles, negation, and certain prosodic patterns).

In particular, much previous work has shown that polar questions can take all sorts of forms, involving sentence structures and intonation patterns diverging from the ‘canonical’ polar question form in (1). Consider, for instance, the examples in (2).

(2) a. Whales are mammals, aren’t they?       inverse polarity tag question

   b. Whales are mammals, right?           confirmation tag question

   c. Aren’t whales mammals?             high negation question

   d. Are whales not mammals?             low negation question

All of the question forms in (2), like the one in (1), raise the same issue (whether or not whales are mammals), yet they differ from each other in terms of sentence structure and intonation contour. The situations in which these forms can be felicitously uttered, i.e., their contexts of use, differ as well. Indeed, the questions in (2) are generally referred to as *biased* questions, because they are only used in contexts which involve certain expectations on the part of the speaker and/or certain contextual evidence for or against the proposition that *whales are mammals* (e.g., [3–7]; see [8] for an overview).

While there is a vast body of empirical and theoretical work available on polar questions in a number of spoken languages, we know comparatively little about the ways in which (biased) polar questions are expressed in sign languages. Indeed, the WALS and APiCS typological surveys cited above are based on samples which only contain spoken languages. Although for a range of sign languages we know how ‘canonical’ polar questions (purely information-seeking polar questions occurring in out-of-the-blue contexts) are expressed, and for some sign languages a handful of specific types of ‘non-canonical’ polar questions have been investigated (see the section *Previous work on polar questions in sign languages* for an overview), there are hardly any sign languages for which the forms and contexts of use of canonical and non-canonical polar questions have been systematically and comprehensively investigated.

The aim of the present paper is to contribute toward diminishing this knowledge gap by investigating polar questions in one specific sign language, namely Dutch Sign Language (*Nederlandse Gebarentaal*; NGT). This sign language is also referred to in the academic literature as *Sign Language of the Netherlands*. However, there have been signals that the Dutch deaf community prefers NGT to be referred to in English as *Dutch Sign Language*. This impression is echoed in a recent master’s thesis [9]. Moreover, as Klomp [10] reports (based on information in [11]), the use of NGT extends beyond the Netherlands to at least Suriname and the (former) Netherlands Antilles, which used to be Dutch colonies. We therefore choose to refer to the language as *Dutch Sign Language* here.

In the case of NGT, it has been proposed that raised eyebrows and a forward position of the head are the most important features of canonical polar questions [10,12,13]. These non-manual markers have been associated with canonical polar questions in various other sign languages as well, often in combination with wide-open eyelids, eye contact with the addressee, and a forward position of the body [14,15]. Yet, several experimental and corpus-based studies indicate that there is, in fact, quite a lot of variation in how polar questions are expressed in NGT and other sign languages (see *Previous work on polar questions in NGT* for further discussion). Our general hypothesis is that much of this variation can be better understood if we take *bias* into account. Just like English and other spoken languages have many different polar question forms connected to restricted contexts of use, we expect that NGT and other sign languages also have many different polar question forms whose contexts of use are conditioned by bias.

Concretely, we will address the following two research questions:

1. **Sentence structures**
  (a) Which sentence structures are used to express polar questions in NGT?
  (b) In which kinds of contexts can each sentence structure be felicitously used? In particular: How do signer expectations and contextual evidence affect the felicity of the different polar question forms?
  (c) What are the communicative functions of different structural elements that may be used in polar questions in NGT, such as polarity marking and question tags?
2. **Non-manuals**
  (a) Which non-manual markers co-occur with the different sentence structures that are used to express polar questions in NGT?
  (b) In which kinds of contexts are these non-manual markers used?
  (c) What are the communicative functions of these non-manual markers when used in polar questions?

To address these questions, we developed a production task in which signers of NGT were prompted to ask questions in different situations. The signer’s expectations with respect to the truth of the proposition that the question was about and the contextual evidence with respect to this proposition were manipulated through role play with two confederates. This allowed us to identify many different (biased) polar question forms, and to observe in which types of context each of these forms was used.

The paper is structured as follows. In the *Background* section, we introduce some central notions in the analysis of polar questions, and provide an overview of relevant previous work on polar questions in sign languages. The section *Materials and methods* describes the production experiment. The processing and annotation of the resulting dataset is discussed in the section *Dataset and annotation procedure*. The section *Results: Polar question structures and their contexts of use* describes the sentence structures found among the polar questions in the dataset, based on which a number of generalizations are proposed about their contexts of use in the section *Discussion: Polar question structures and their contexts of use*. The section *Results: Nonmanuals* presents non-manual marker patterns in the data and the functions of these non-manuals are analyzed in the section *Discussion: Nonmanuals*. *Limitations and future work* addresses limitations of the study and avenues for future research. The *Conclusion* section concludes the paper.

## Background

This section provides an overview of relevant previous work. The literature on polar questions is too extensive to be reviewed exhaustively here. We focus on describing and illustrating the main parameters that have been used in characterizing the felicity conditions of various polar question forms, and on work which is concerned with polar questions in sign languages and, more specifically, in NGT.

## Central notions in the analysis of polar questions

Two contextual parameters have played a central role in characterizing the contexts of use of polar question forms across languages: *prior speaker belief* (or, in the context of sign languages, *prior signer belief*) and *contextual evidence* (e.g., [3,4,16–19]) Prior speaker belief (SB) refers to the speaker’s belief about the truth of the proposition *p* expressed by the sentence radical—in (2), the proposition that *whales are mammals*—prior to the current conversational context. Contextual evidence (CE) refers to evidence concerning the truth of *p* that is provided in the conversational context, immediately before the question is asked.

To illustrate the relevance of SB, consider the scenario in (3):

(3) Scenario: Ann and Sue are gathering with a group of classmates in front of the cinema to go see a movie. Several people have already arrived. Ann asks “Are we all here?”. Sue responds:

a. Mary is coming as well, isn’t she?          inverse polarity tag question
b. Mary is coming as well, right?             confirmation tag question
c. Isn’t Mary coming as well?                high negation question

The tag questions in (3-a-b) and the high negation question in (3-c) are felicitous in the given scenario if Sue believes that Mary is indeed coming (positive SB), but not if she believes that Mary is not coming (negative SB) or if she has no idea whether Mary is coming or not (neutral SB). More generally, in English, tag questions and high negation questions can only be used felicitously in contexts with positive SB.

To illustrate the relevance of CE, consider the scenario in (4).

(4)  Scenario: Ann and Sue want to go see a movie with some classmates. They are discussing who to invite. Sue says “I only want to invite Peter and Mary”. Ann responds:

a. Do you want to invite Peter?               canonical polar question
b. # Do you not want to invite Peter?             low negation question
c. # Do you want to invite Sebastian?            canonical polar question
d. Do you not want to invite Sebastian?            low negation question

In the given scenario, there is clear CE *for* the proposition that Sue wants to invite Peter, and *against* the proposition that Sue wants to invite Sebastian. As seen in (4-a-d), this affects the felicity of canonical polar questions and low negation questions asking about Sue’s interest to invite Peter or Sebastian. In general, in English, a low negation question of the form ‘not *p*?’ requires CE *against p*, while a canonical polar question of the form ‘*p*?’ is compatible with CE *for p* and with contexts in which there is no clear evidence with respect to *p*, but not with CE *against p* [16].

While contextual parameters other than SB and CE have been argued to be relevant for characterizing the felicity conditions of certain polar question forms as well (e.g., whether the speaker *wants p* to be true; see [20,21]), we will restrict our attention in this paper mainly to these two factors, which have been of central interest in previous literature.

### Previous work on polar questions in sign languages

Zeshan [14,15] provides a typological overview of interrogatives in sign languages, focusing mainly on *canonical* question forms. Based on data from 37 sign languages (though in some cases the amount of available data for a given language is minimal), Zeshan concludes that canonical polar questions are most commonly marked by non-manual markers alone (without specific manual signs or a characteristic constituent order). Raised eyebrows, wide-open eyelids, eye contact with the addressee, and a forward head/body position are cross-linguistically the most common markers. Zeshan further notes that, depending on various factors discussed in no further depth, signers may use different non-manuals than these, such as “lowering instead of raising of the eyebrows in pragmatically marked questions” ([14], p. 20), like when expressing doubt (cf. [22]). About one fourth of the sign languages in Zeshan’s sample have optional manual question particles, with East Asian sign languages being particularly rich in them. Subject pronoun doubling and verb doubling are also common in canonical polar questions across sign languages.

Not much is known about *non-canonical* polar question forms in sign languages. Question tags have been mentioned briefly for Italian Sign Language [23], for Chinese Sign Language [24], and for British Sign Language [25]. Negative polar questions have been described for New Zealand Sign Language, Japanese Sign Language, American Sign Language (ASL), Croatian Sign Language (HZJ), Turkish Sign Language (TİD), Flemish Sign Language, and Indo-Pakistani Sign Language (for an overview, see [26]), Swedish Sign Language [27], Catalan Sign Language (LSC; [28]), and Hong Kong Sign Language (HKSL; [29]). Most of these studies report that negative polar questions combine (non-manual) markers of negation and interrogativity, for instance a headshake or backward head movement to mark negation, and raised eyebrows to signal that the utterance is a polar question. Additionally, brow furrowing and forward or backward head thrusts have been observed to occur in polar questions (e.g., in HKSL and LSC) and are said to mark surprise or doubt. For ASL, both Gökgöz and Wilbur [26] and Gonzalez and colleagues [30] report that, when negation is expressed manually, negative polar questions sometimes lack non-manual marking of negation (a headshake), while in negative assertions non-manual marking of negation is obligatory.

Very few studies have investigated the contexts of use of canonical and non-canonical polar question forms in sign languages. Gökgöz and Wilbur [26] and Sze and Lee [29] investigated the form and contexts of use of non-canonical polar questions involving negation in HZJ, ASL, and TİD. In terms of form, their main finding is that in HZJ and TİD negative polar questions exhibit a particular, rather fixed combination of non-manual markers, while in ASL there is not one particular pattern that stands out as there is quite some variation. A representative example from TİD is given in (5) (example from [26], p. 27; adapted to match our glossing conventions). In this example, backward head movement (‘h.bw’) and upward chin movement (‘ch.u’) signal negation, while the forward head movement (‘h.fw’), downward chin movement (‘ch.d’) and eyebrow raise (‘br.r’) are typical non-manual markers of polar questions. The translation suggests that the authors interpret the construction as a high negation question. Indeed, Gökgöz and Wilbur argue that “a positive epistemic implicature arises in TİD when a yes/no question bears negation” (p. 27), whereas negation is claimed to be interpreted low in negative polar questions in HZJ and ASL.



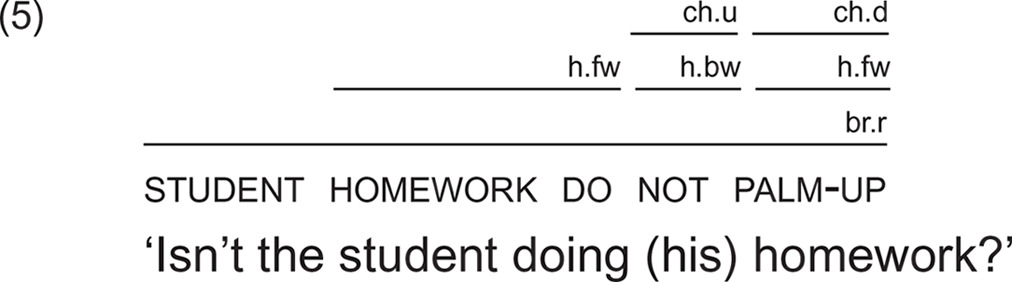



As for the interpretation of negative polar questions and the contexts in which they can be used, the main finding in [26] is that negative polar questions in TİD, like the one in (5), signal positive SB, but negative polar questions in HZJ and ASL do not, i.e., they are neutral with respect to the signer’s prior belief. Gökgöz and Wilbur do not consider CE as a contextual factor.

Sze and Lee [29] investigate the form and contexts of use of negative polar questions in HKSL. They consider four types of contexts, of which we discuss the three most relevant ones here. In the first type of context, there is (implicit) positive SB and neutral CE. In this case, negative polar questions, and high negation questions specifically, do not seem to be licensed in HKSL. In the second and third type of context, there is positive SB and negative CE. The difference is that in the second type of context, the positive SB is explicit, while it is implicit in the third type. The second type of context is reported to trigger high negation questions, while the third triggers low negation questions. These different question types are claimed to be distinguished through different non-manual markings, although the specific non-manual marker patterns used are also reported to be inconsistent between signers.

Finally, Cañas Peña [28] investigates the contexts of use of canonical and various non-canonical polar question forms in Catalan Sign Language (LSC). More specifically, Cañas Peña elicited polar question forms in different contexts from two native LSC signers. In these contexts, epistemic bias (comparable to SB in our study) and evidential bias (comparable to our CE) were manipulated. In a follow-up step, the same signers were shown different combinations of contexts and question forms to determine which forms could be used in which contexts. Cañas Peña found that different combinations of epistemic and evidential bias were associated with different combinations of brow configuration (raised vs. furrowed) and head and body position (backward vs. forward). In total, Cañas Peña describes five different non-manual marker combinations. Example (6) ([28,28], p. 12; ‘b.fw’ = body forward) illustrates one such combination (brow raise, head and body forward), felicitous when epistemic bias is positive and evidential bias is not negative (e.g., in (6-a)), and infelicitous otherwise (e.g., in (6-b)). The English translation and context descriptions have been slightly adapted for clarity.



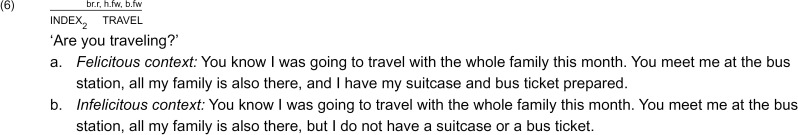



Although the study by Cañas Peña considers various polar question forms and various contexts differing in terms of SB and CE, it does not cover the entire spectrum: in total, five polar question forms are considered and each is associated with a particular combination of SB and CE. For two possible combinations of SB and CE (neutral SB with positive CE, and neutral SB with negative CE), Cañas Peña leaves open which polar question form(s) they are compatible with. The range of polar question forms that are investigated is most likely not exhaustive either, because question forms involving negation are not taken into consideration.

### Previous work on polar questions in NGT

The reference grammar for NGT by Klomp [10] characterizes canonical polar questions as being marked by raised eyebrows and a forward position of the head. The grammar mainly draws on the work of Coerts [12] and inspection of the Corpus NGT [31], and its findings align with [13], who find exactly the same non-manuals in canonical polar questions in NGT. Klomp adds that the forward position of the head is often combined with a head turn or tilt, and that polar questions also optionally involve a manual element, namely the sign palm-up, which usually appears sentence-finally (see also [32]). Finally, Klomp notes that polar questions and declarative clauses do not differ from each other in terms of constituent order (again, see also [13]).

It is worth summarizing the work by Coerts [12], the main source for of information for Klomp, in a bit more detail. Coerts elicited data from 19 informants, all L1 signers of NGT of different ages and from different regions in the Netherlands. During the elicitation sessions, informants worked in pairs and were given four tasks. Three of these involved storytelling by just one of the informants. In these tasks, polar questions directed toward the addressee only occurred twice. The fourth task was a guessing game: one informant was given a drawing of a certain object and the other informant had to find out what kind of object this was by asking questions. This yielded a total of 75 polar question instances.

Across these 77 instances in total, the marker ‘head forward’ occurred 78% of the time, while ‘raised eyebrows’ occurred 57% of the time. They occurred together in 51% of the cases, so 27% of the cases featured only ‘head forward’ without ‘raised eyebrows’ and 6% of the cases featured only ‘raised eyebrows’ without ‘head forward’. Almost all of the polar questions that did *not* involve either of these two markers did involve one or more of the following markers: ‘eyes wide open’, ‘head tilt’, ‘body/shoulder up/forward’. Coerts remarks that “one could argue that these features replace the characteristic features ‘eyebrows up’ and ‘head forward’ in certain contexts” (p. 107). Only in one case, the markers ‘head and body backward’ and ‘brows down’ were observed.

It is important to note that the task in Coerts’s study was such that the informants asking questions generally did not have any particular prior belief with respect to the answer (they had no idea what the object in the drawing was), and the context generally did not provide any evidence leading them to expect a particular answer either. Further, they presumably did not have a preference for any particular answer, and likely asked their questions in a polite and emotionally neutral way.

Besides Coerts, the only other previous experimental study of polar questions in NGT is that of De Vos and colleagues [33]. In this study, two L1 signers of NGT were shown videos of ten polar questions and were asked to pose the same ten questions in a neutral, angry, and surprised affective state, resulting in 30 target items per participant. The questions did not involve negation or question tags.

The analysis focused on the position of the eyebrows only. Video recordings were annotated using the Facial Action Coding Scheme (FACS, [34]). This scheme includes an annotation value for raised eyebrows (AU 1 + 2) and one for lowered eyebrows (AU 4), as well as a third value for a configuration of the face which results from engaging both the brow lowering muscles and the brow raising muscles simultaneously (AU 1 + 2 + 4). In terms of eyebrow position, this facial configuration is much more similar to a configuration with lowered eyebrows than to one with raised eyebrows.

When participants posed the ten questions in a neutral affective state, raised eyebrows (AU 1 + 2) occurred 67% of the time, while lowered eyebrows with forehead wrinkles (AU 1 + 2 + 4) were attested in the remaining 33%. In an angry state, lowered eyebrows (AU 4) occurred in 78% of the cases, while the remaining 22% involved either sequential or simultaneous combinations of AU 1 + 2 and AU 4. Finally, in a surprised affective state, AU 1 + 2 was observed in 58% of the cases, AU 1 + 2 + 4 in 17% of the cases, and sequential or simultaneous combinations of AU 1 + 2 and AU 4 in the remaining 25%. The authors further report that the intensity of the brow raises in a surprised affective state was higher on average than in a neutral affective state, though they also note that annotations of the intensity of facial action units may not always be reliable/reproducible.

Overall, these results indicate that (i) there is considerable variation in eyebrow configuration in polar questions even in a neutral affective state, and (ii) the signer’s affective state can have a considerable impact on eyebrow configuration. In particular, in an angry affective state, polar questions frequently involved ‘pure’ lowered eyebrows (AU 4) while in a neutral or surprised affective state, lowered eyebrows were attested less frequently and only in combination with AU 1 + 2, resulting in forehead wrinkles. We hypothesize that contextual parameters like SB and CE similarly affect eyebrow configuration and other aspects of the form of polar questions. The production experiment detailed in the following section serves to test this hypothesis.

## Materials and methods

To address our two main research questions, we designed a production experiment to elicit (biased) polar questions in NGT in different contexts. In a role-play setting, adult signers who acquired NGT by the age of four were prompted to ask questions to two signing confederates, whose responses introduced different prior signer belief (SB) and contextual evidence (CE). These exchanges were intended to trigger a target question directed toward the second confederate at the end of each role play.

In the sections below, we provide details about the study participants, the experimental stimuli, the studio where the sessions took place, and the experimental procedure. Part of this section is an abridged version of a document we previously published at https://doi.org/10.21942/uva.21701954.v2 to make it possible for other researchers to set up similar experiments using comparable materials. Data processing and annotation are discussed separately in the section Dataset and annotation procedure.

Prior to data collection, the study was approved by the Ethical Committee of the Faculty of Science (FEC) of the University of Amsterdam (reference FEC 2022−01). All participants gave written informed consent for the processing, analysis, and online archiving of the video-recorded data by the researchers, as well as discussion of the data in academic publications (in anonymized form). All participants except the trial participant gave written consent for making the video-recorded data publicly accessible (in non-anonymized form) e.g., by showing (fragments of) the recordings in academic and general publications and presentations, and in education and courses; for making the data available in an online research data repository; and for sharing the recordings with other researchers. The individuals pictured in Fig 4–8 have provided written informed consent (as outlined in the PLOS consent form) to publish their image in this article.

### Participants

Participants were recruited between 26 April and 12 July 2022 via a video-recorded call for participation in a study on questions in NGT, placed on the Facebook page of the university’s sign language group. Eight deaf signers of NGT responded to the call. One of the signers participated in a trial session to evaluate and optimize the experimental procedure and stimuli; the data resulting from this session were not analyzed and are not publicly available. After annotating the data of the remaining seven signers, we decided to exclude the data from one participant from the study. The signer in question had indicated in the participant survey that they began learning Sign Supported Dutch from the age of four (primary school) and only started learning NGT in high school. Because the participant indicated that they used NGT as their primary means of communication in daily life, the signer was allowed to participate in the study. However, the influence from Sign Supported Dutch was obvious in their signing, and their productions clearly diverged from those by the other signers. Since our main focus lies on the expression of different kinds of questions in NGT, we decided to exclude this participant from the study. Thus, we ended up including the data of six of the participants in our analysis.

Prior to the start of the study, participants answered a number of questions about their (language) background via the online survey platform Qualtrics. The questions, most of them multiple-choice, were presented in Dutch only. Of the six signers whose data were analyzed – all of them women –, two were between 18 and 29; one between 30 and 39, and three between 50 and 59 years of age. All signers self-identified as deaf, and one of the participants reported having a cochlear implant. Five of the signers reported to be right-handed and one ambidextrous. All participants reported that they use NGT on a daily basis, and all indicated that they acquired NGT either from birth or within the first four years of life. In the Netherlands, there are five main variants of NGT connected to the locations of the (former) five deaf schools in Groningen (North East), Amsterdam (North West), (Sint-Michiels)gestel (South), Rotterdam, and Voorburg (both West) [35,36]. All variants except one (Gestel) are used by the participating signers. Rotterdam (N = 2) and Voorburg (N = 2) are both represented by two signers, while the Groningen and Amsterdam variants are represented by one signer each. All participants indicated that they use Dutch either on a daily basis or most days. Other languages used by participants include English (‘most days’, N = 1; ‘occasionally’, N = 2) and American Sign Language (‘occasionally’, N = 1). Three of the signers also reported that they occasionally use International Sign. All except one signer, whose parents are both deaf, have hearing parents. Approximately half of the hearing parents were reported to know little to no NGT; the reported signing skills of the remaining hearing parents ranged from ‘limited’ to ‘good’.

Participants were financially compensated for their time and travel expenses.

### Stimuli

Questions were elicited from participants in a role-play setting in which they interacted in NGT with two members of our research team, who we refer to as confederates A and B. Both are deaf and early acquirers of NGT. During the experimental sessions, the confederates were referred to as *Ria* (confederate A) and *Tom* (confederate B) to contribute toward the sense of a play-acting setting. The confederates signed pre-scripted utterances in response to participant productions that were prompted by stimulus materials projected on a laptop screen. The confederates’ utterances were intended to introduce SB (confederate A) and CE (confederate B). The only language used during the experimental sessions was NGT.

We created six situations (one used as practice trial) designed to elicit polar questions from participants in different contexts. The situations were loosely based on selected scenarios from a study on biased polar questions in spoken German and English [19], which was not a production experiment but a forced-choice task using written stimuli in which participants had to pick the best option from a number of sentence forms given a particular context description. For each situation, there were seven experimental conditions with different combinations of SB and CE (Table 1), which we refer to as variations. Only double positive and double negative evidence combinations were not included in the study, as it is unnatural to ask a question in such cases. Note that Domaneschi et al. [19] tested only six experimental conditions in their study, because they also excluded the combination negative SB – neutral CE. While it can indeed be somewhat unnatural to ask a question in such a setting, it is not impossible, so we decided to include this condition in our task. All situations in the experiment are described in English in S1 File.

**Table 1:** Experimental conditions.

	Prior signer belief		
Contextual evidence	*Positive*	*Neutral*	*Negative*
*Positive*		X	X
*Neutral*	X	X	X
*Negative*	X	X	

**Fig 1:**
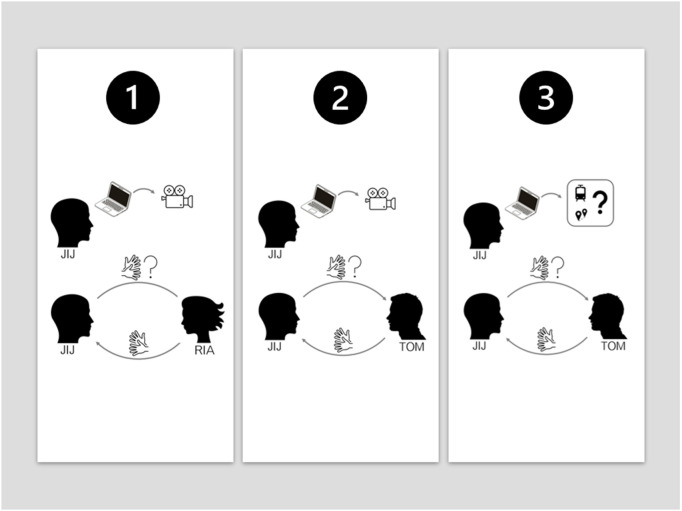
Visualization of the structure of each experimental trial. *Jij* = ‘you’ in Dutch. *Ria* and *Tom* are the confederates. Reprinted from [37] under a CC BY license, with permission from University of Amsterdam / Amsterdam University of Applied Sciences, original copyright 2022.

Each trial, i.e., each variation within a situation, consisted of three short interactions between the participant and confederate A (first interaction) and confederate B (second and third interaction). The structure of each trial is visualized in Fig 1, which was also printed on a poster and hung in the studio where the experimental sessions took place.

Participants first saw a context video on a laptop screen, prompting them to ask a question to confederate A, who through their answer introduced positive, neutral, or negative SB with regard to the issue at stake (e.g., whether or not Kim is a vegetarian). In the second interaction, participants watched another context video in which they were instructed to ask another question to confederate B, who then gave a response which provided positive, neutral, or negative CE for the question at issue. Participants were subsequently shown a picture prompt on the laptop, which was intended to trigger participants to ask another question, the target question, to confederate B in the final interaction. After the participant asked the target question, confederate B gave a brief unscripted response to conclude the role play.

Participants were instructed to minimally include the concepts that were represented in the picture prompts. For instance, in situation 1 (see S1 File), the concept ‘vegetarian’ is represented by the V label (logo for vegetarianism) with two small icons of an apple and corn, and ‘Kim’ is represented by a silhouette of a woman’s head and the word ‘Kim’ below it. The question mark on the right was intended to remind the participant that their response should come in the form of a question, not a statement. Any images that were unclear to the participant were explained during the practice round of each first variation of a situation (see the section *Experimental procedure* for more details on the experimental procedure). We chose to use picture prompts for triggering the target question in order to avoid having to use video recordings of signs, which could influence participants in terms of lexical choices but also sign order. We also purposely aligned the pictorial representations of concepts vertically rather than horizontally. However, we realize that this vertical alignment could still have an effect on constituent order. We therefore created two sets of picture prompts, where the top and bottom images on the left of the prompts were reversed (see S1 File for all picture prompts). Two participants saw version 1 and four participants saw version 2 of the picture prompt set. A seventh participant saw version 1 but was excluded from the study after collection of the data; hence the imbalance in the number of participants who saw each version.

All stimulus materials are available online. The context videos, signed in NGT, can be found at https://doi.org/10.21942/uva.21695150, and recordings of the scripted confederate utterances can be found at https://doi.org/10.21942/uva.26870170. All target utterances from our participants (the final utterance in each trial) are available at https://doi.org/10.21942/uva.21666203.

### Recording studio

**Fig 2:**
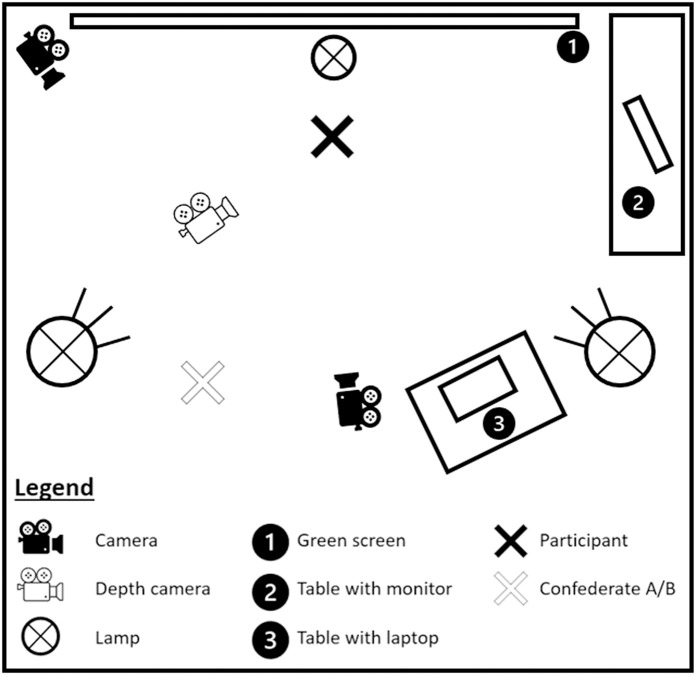
Overhead view of the recording studio setup. Reprinted from [37] under a CC BY license, with permission from University of Amsterdam / Amsterdam University of Applied Sciences, original copyright 2022.

The experimental sessions took place at a recording studio at the University of Amsterdam, equipped with studio lighting and a green screen. Fig 2 presents an overhead view of the studio set-up during the sessions.

Participants were stood in front of a green screen. Confederates, when in dialogue with the participant, stood slightly toward the signer’s right, positioned exactly between two cameras on tripods. They would stand and wait at the back of the room when not interacting with the participant. We used two cameras to record the participant: a Sony FDR-AX53 (Full HD) positioned slightly toward the signer’s left (confederate’s right) and an iPhone with depth camera for 3D recording, toward the signer’s right (confederate’s left). A second Sony FDR-AX53 camera (top left corner in the figure) recorded the confederates, to make it possible later on to do a double-check when needed, e.g., in case of an unexpected utterance from the participant in reaction to a confederate. The confederate recordings made during the experimental sessions are not publicly available, but we recorded the scripted confederate utterances separately after the sessions (available at https://doi.org/10.21942/uva.27043453).

In addition to the participant and confederates, there were two experimenters in the room. The first stood behind a high table (3 in Fig 2) with a keyboard and mouse connected to a laptop facing the signer (also on table 3), and a monitor visible to both the confederates and the experimenter on a low table (2). This experimenter guided each session and was responsible for providing instructions, projecting the stimuli (see the section *Stimuli*) on the laptop and monitor, as well as handling the participant camera. A second experimenter was positioned between the confederate camera and the studio lamp on the left in Fig 2, and was responsible for handling the confederate camera and depth camera. This experimenter also kept track of the trial number on a poster on the wall as well as on a clapperboard, which was displayed in front of the cameras at the start of each trial.

### Experimental procedure

Participants were given a brief introduction by the lead experimenter and were then shown pre-recorded instruction videos in NGT on the laptop on Table 3. In the video, participants were told that they were going to participate in short role plays together with the confederates, who were introduced as ‘Ria’ and ‘Tom’ and referred to with initialized sign names, and that they had to ask these confederates a number of questions during the role plays. The sign name for ‘Kim’, which was used in two of the situations, was also introduced in the instruction video. The participants then witnessed two example role plays in real time, where the lead experimenter took the role of the participant and played two variations within the practice situation with the confederates (see S1 File). The lead experimenter is a hearing L2 signer of NGT with nine years of signing experience at the time the data collection took place. She uses NGT mostly in interaction with signing colleagues and participants.

Then, the structure of a role play was explicated using the visualization in Fig 1. Participants were instructed that they always had to ask questions to confederates, but that there were no restrictions on sign order or use of facial expressions. They were instructed to keep their productions brief, preferably restricted to a single sentence, and to sign them as naturally as possible. It was explained that there were five different situations with seven different variations within each situation, as well as a practice situation. Participants were told that, within each situation, the context videos and picture prompts were always identical, but that the responses of the two confederates would differ from variation to variation. Participants were instructed to adapt their questions depending on the confederates’ utterances, meaning that they might want to ask the same question slightly differently for each variation within a situation. To help participants get a sense of what we were after, they were shown three examples of variations of the same base question (e.g., “Is Kim home?”), but with different use of facial expressions and other non-manual markers (e.g., headshake for negation).

Then the practice session commenced, which included several variations within the practice situation (see S1 File). This allowed the participants to get used to the structure of each role play and to experience how the variations within a given situation would slightly differ from each other and as a result may lead them to ask the final question in each variation in a different way. In our experience, the practice phase usually took up quite some time, but once participants got the hang of the experimental procedure, the actual experimental phase would typically go rather smoothly. The main phase of the experiment began after the practice session and after participants had no more questions about the procedure. In total, all sessions lasted between 3–4 hours including breaks. Usually, the instructions and the practice session lasted about 1.5 hours, after which the tempo sped up considerably.

Remember from the section *Stimuli* that, in each trial, participants were presented with context videos preceding their first two interactions with the confederates. In practice, these context videos were not replayed over and over again. Rather, when the first variation within a new situation was introduced, participants would take some time to absorb the information in the context videos and to remember the questions they had to ask to the confederates. They would then do a practice run, only after which we would start recording. The context videos would not be shown anymore after this, unless the signer requested it. Each trial, then, consisted solely of interactions between the participant and the confederates, and the laptop where videos and stimuli were presented would display just the picture prompt for the target question. This way of going through the trials worked well and generally made the participant’s interactions with the confederates feel more natural, both to the participant and to the observing experimenters.

## Dataset and annotation procedure

After data collection, we processed the resulting video recordings and prepared them for annotation. As we detail in the sections below, the subsequent annotation procedure consisted of four steps. We first delimited the scope of each target question and glossed the manual signs used in them with ID-glosses. Then, we enriched the dataset with (i) annotations for sentence structure, (ii) non-manual markers, and (iii) other potential elements of interest.

### Dataset and data processing

The data of all six participants whose productions were analyzed are available at https://doi.org/10.21942/uva.21666203. Each file includes a compilation of five target questions involving the same participant and the same experimental condition (combination of SB and CE), one for each of the five different situations represented in S1 File. Video file names refer to the participant number, stimuli version (picture prompt set; see the section *Stimuli*), and experimental condition, where, e.g., ‘PosNeut’ stands for positive SB and neutral CE. For all participants, there is also a video with ‘baseline’ questions; these include for each situation one of the questions asked by the participant to confederate A at the beginning of each variation. Since these questions were asked without any bias yet involved, we considered these baseline, or ‘out of the blue’, polar questions. For participants with participant numbers 03, 05, 06, and 07, we also elicited declarative versions of the target questions for each situation (e.g., ‘Kim is a vegetarian’); these recordings are also included in the online dataset. No such recordings were made with participants 01 and 02.

Thus, the dataset comprises 52 files with five target sentences each, amounting to 260 constructions in total. This total includes 30 baseline questions (five from each signer) and 20 declarative sentences (five each from four signers). These 50 sentences were all annotated but will not be discussed in detail in the results sections below. We simply remark here that the declarative sentences all consisted of a single clause and did not involve overt polarity marking except in two cases in which the signer used subtle head nodding. The baseline questions involved somewhat more variation, albeit considerably less so than the question forms elicited in the various experimental conditions. Six baseline questions involved embedding (“Do you know if...”; cf. section *Sentences with embedding*), 14 consisted of a single clause without polarity marking (cf. section *Sentence radical only*), and ten were made up of a sentence radical (SR) followed by an *or...* tag or a hesitation tag (cf. section *Sentence radical and tag(s)*). Of those ten questions, two involved positive polarity marking, while the others did not show marking of polarity.

Table 2 indicates the number of target questions included in the analysis per condition. A total of 210 target questions were elicited across the six participants and seven experimental conditions. A total of thirteen of these question sentences – generally one or two per condition – had to be excluded from the analysis, leaving 197 questions for analysis.

**Table 2:** Number of included target questions per condition.

	SB		
CE	*+*	*0*	*–*
*+*		28	28
*0*	29	30	29
*–*	28	25	

In most cases, the reason for exclusion was that the construction was unanimously assessed by two of the authors (two hearing L2 signers of NGT) and a deaf L1 language consultant as (i) not representing a question sentence (N = 4), or (ii) a clear mismatch with the experimental condition (N = 5). In two cases, the signer used a predicate sign antonymic to the target predicate (closed instead of open), inversing the polarity of the sentence which inadvertently complicated the analysis of the results. In one case, the signer inquired into the interlocutor’s knowledge about the issue at stake (“Did you know that...”) rather than the issue itself. One construction had a structure that was unclear to both the authors and the language consultant.

It can be observed that in the condition with neutral SB and negative CE, a total of five constructions were excluded; in four of those cases the reason for exclusion was that the elicited question form was judged not to be a good match with the preceding context. It is possible that this particular combination of SB and CE renders the asking of a follow-up question somewhat unnatural, making it harder for participants to provide a fitting response. Nonetheless, we obtained 25 question sentences that were suitable for analysis in this condition.

The remaining 210 target questions, plus the baseline and declarative sentences, were subsequently glossed and annotated for manual and non-manual markers in ELAN Linguistic Annotator across a set of 21 newly created tiers. All steps of the annotation procedure are described in the subsections below. The annotation files are publicly available at https://doi.org/10.21942/uva.22737074.

### Delimiting and glossing target questions

We first determined the scope of each target question by creating time-aligned annotations on the main tier labeled ‘Question’, and subsequently gave it a unique identifier code indicating participant number, condition (e.g., PosNeg for positive SB and negative CE), and situation number.

On two child tiers, we added ID-glosses for manual signs, both in English (‘Glosses_EN’) and in Dutch (‘Glosses_NL’). The glosses correspond to the ID-glosses in the NGT database on Global Signbank (https://signbank.cls.ru.nl/). Signs that were not included in Signbank at the time data annotation took place, such as vegetarian, were prefixed with % in the annotations on these tiers. In Global Signbank, lexical variants of the same concept are distinguished from one another with letter suffixes in the ID-glosses (e.g., free-of-charge-a and free-of-charge-b); these are also included in the annotations, as well as in the glossed examples that we include in this paper. A comment tier (‘Comments’) was also included.

The annotations for this first triptych of tiers were made by the first author of this research paper, a hearing L2 signer. Cases of uncertainty were discussed with the NGT consultant.

### Annotating sentence structure

Annotations for sentence structure were made across three tiers. On the tier ‘Structure’, a child tier of the main ‘Question’ tier, we indicated the general sentence structure of each question. There were five possible annotation values for this tier, listed in Table 3.

**Table 3:** Annotation values on the tier ‘Structure’.

Value	#	Description
*EXCL*	13	Excluded from analysis
*EMB*	18	Complex sentence with embedding
*SR*	114	Sentence radical only
*SR-T1*	63	Sentence radical followed by a tag
*SR-T1-T2*	2	Sentence radical followed by two tags

Firstly, constructions that were excluded from the analysis, of which there were thirteen (see the section *Dataset and data processing*), were labeled ‘EXCL’. Secondly, constructions involving embedding (N = 24) were labeled ‘EMB’. For questions without embedding, there were three possible annotation values. Preliminary analysis of the data revealed that each question sentence consisted of either just the SR (‘SR’), or a SR followed by one (‘SR-T1’) or two (‘SR-T1-T2’) sentence-final phrases, which we refer to as *tags*. We define tags as particular combinations of manual and non-manual markers following the SR, fulfilling a specific semantic function (see the section *Sentence radical and tag(s)* for examples and discussion).

On the tier ‘Structure-components’, a dependent of the ‘Structure’ tier, we specified further details about the properties of SRs and tags, if present. The annotations on this tier concerning the SR took the form of combination labels indicating (i) whether the SR was overtly marked for polarity, and (ii) whether it included a prosodically integrated palm-up sign. palm-up (see Fig 4 and 7 for examples) is a frequently used sign/gesture in both sign languages and (co-speech) gesture expressing a wide range of epistemic meanings [38]. Table 4 gives an overview of the annotation labels and frequencies.

**Table 4:** Annotation values and frequencies for SRs on the tier ‘Structure-components’ (N = 179).

Value	#	Description
*SR.pos(.PU)*	62 (6)	SR with positive polarity marking (and prosodically integrated palm-up)
*SR.no-pol(.PU)*	60 (5)	SR without polarity marking (and prosodically integrated palm-up)
*SR.neg(.PU)*	43 (3)	SR with negative polarity marking (and prosodically integrated palm-up)

All SRs are included, including those followed by one or two tags.

Polarity was often marked non-manually by signers by means of head nodding or headshaking. Concerning head nodding, both single and repeated head nods were attested in the data. Single head nods were not annotated as markers of positive polarity on the sentence structure tiers, because all instances appeared to fulfill functions other than polarity marking. It is known, for instance, that a single head nod in NGT may be used to signal imperatives [39], to mark an intonation phrase boundary and/or to signal emphasis [10]. In contrast, instances of repeated head nodding, typically spanning multiple signs, were analyzed as positive polarity markers. Headshake is a known marker of negation in NGT [12,40], but – as we shall see – it may also fulfill other types of (pragmatic) functions, in particular when it occurs on certain types of tags (see the section *Hesitation tag* for discussion). When a headshake accompanies the SR, we found that it usually served a negation function, thus marking negative polarity. Some SRs (also) included manual polarity markers, such as the basic clause negator not-a or the sign wel, which appears to function similarly to the word ‘wel’ in spoken Dutch, a multifunctional particle signaling positive polarity [41]. Given these commonalities, and given that English does not possess a suitable translational equivalent, we use the Dutch Signbank ID-gloss wel rather than the English one (yes-a). SRs that were marked for positive and negative polarity, manually or non-manually, were annotated as ‘SR.pos’ and ‘SR.neg’, respectively. SRs that were not overtly marked for polarity received the annotation ‘SR.no-pol’. The annotation labels for SRs that included a prosodically integrated palm-up sign – that is, a palm-up not functioning as (part of) a tag – received the suffix ‘.PU’.

For the tag parts of SR-T1(-T2) constructions, we annotated the tag type. The options are listed in Table 5; see the section *Sentence radical and tag(s)* for descriptions and examples of all tag types.

**Table 5:** Annotation values for tags on the tier ‘Structure-components’.

Value	#	Tag type
*HES(.inqhs)*	20 (19)	Hesitation tag (with inquisitive headshake)
*TOCH*	11	toch tag
*ORpos / ORneg*	3 / 8	*Or*... tag (with inquisitive headshake)
*CONF*	4	Confirmation tag
*HUH*	2	Confusion tag

Questions labeled ‘SR-T1-T2’ on the ‘Structure’ tier involve two tags; hence the numbers add up to 67 (across 63 sentences with one tag and two sentences with two tags).

Finally, the tier ‘Structure-elements’, also a child tier of the ‘Structure’ tier, was used for free annotation of additional potentially relevant information. For instance, several items included a contrastive discourse particle in the SR, of which note was made on this tier.

The annotation protocol for the structure tiers was developed and evaluated by the first and last author of this paper. Annotations on these tiers were subsequently added by the first author. Cases of uncertainty were discussed with one of the confederates in the production experiment.

### Annotating non-manual markers

All question forms were further annotated for non-manual markers (NMMs) across a set of 12 tiers. For this, we developed, evaluated, and then applied a new annotation guideline that went through several stages of development; we report extensively on this process in [42]. The version of the guideline that was used during the main annotation phase is available at https://doi.org/10.21942/uva.24080868.

For every question form, we made continuous NMM annotations on each of the 12 NMM tiers. We used Controlled Vocabularies containing between three to nine labels, i.e., different non-manual feature specifications, per tier. Table 6 lists the annotation labels available for each tier. The Controlled Vocabularies of all NMM tiers included at least the labels *neutral* and *other* (except ‘NMM.eye-gaze’, which does not include a *neutral* label). *Neutral* was used in cases where the relevant non-manual feature was not actively engaged, e.g., when the eyebrows were in neutral position. *Other* could be selected when none of the annotation labels were considered to fit with the observed NMM. In such cases, the marking had to be specified on the ‘Comments’ tier.

**Table 6:** NMM tiers and annotation label inventories.

Tier name	Annotation labels
*NMM.eyebrows*	neutral, raised, lowered, raised-low, inner-raise, other
*NMM.eye-shape*	neutral, squint-full, squint-half, wide-full, wide-half, closed, other
*NMM.eye-gaze*	addressee, researcher, space, closed, other
*NMM.shoulders*	neutral, up, down, other
*NMM.body-position*	neutral, forward, backward, tilted, sideways, other
*NMM.head-x*	neutral, tilt, other
*NMM.head-y*	neutral, up, down, other
*NMM.head-z*	neutral, forward, backward, other
*NMM.head-move*	neutral, nod, nodding, shake, shaking, sideways, other
*NMM.lip-corners*	neutral, up, down, mouth-action, other
*NMM.lips*	neutral, rolled, pressed, upper-raised, dimpled, puckered, pout, mouth-action, other
*NMM.nose*	neutral, wrinkled, other

20% of the question forms in the dataset were randomly selected for independent annotation by two annotators, a hearing L2 signer of NGT (the first author of this paper) and a deaf L1 signer of NGT. The resulting two sets of annotations were evaluated for inter-rater agreement. The outcomes of this evaluation are reported in detail for all tiers and all annotation labels in a technical report (https://doi.org/10.21942/uva.25563540). Here, we just concentrate on the NMMs which we identified as playing a role of interest in the question forms in our dataset, and which will thus feature prominently in our discussion of the results in the sections *Results: Polar question structures and their contexts of use* (annotations on the ‘NMM.head-move’ tier) and *Results: Nonmanuals* (annotations on other NMM tiers).

The Cohen’s Kappa (κ) scores for the relevant labels are listed in Table 7. Before we further discuss these scores, a couple of notes are in order on (i) the method of calculation of these scores, and (ii) the label selection presented in Table 7.

**Table 7:** Cohen’s κ scores for various NMM labels, under a frame-based approach.

Tier name	Label	κ score
*NMM.head-move*	nodding	0.71
	shaking	0.91
*NMM.eyebrows*	raised	0.72
	lowered	0.59
*NMM.eye-shape*	squint-full	0.57
	wide-full	0.64
*NMM.head-z*	forward	0.38
*NMM.body-position*	forward	0.56
*NMM.lip-corners*	down	0.66

With regard to the first issue, in the technical report, *κ* scores are reported based on two different approaches: a frame-based approach, where inter-annotator agreement is evaluated on a frame-by-frame basis, and an event-based approach, where inter-annotator agreement is evaluated at the level of the event, i.e., the annotation labels assigned to time intervals spanning an entire event rather than individual frames. The event-based approach tends to be somewhat more conservative. While we conclude in the report that the event-based approach – enriched with an automatic error analysis – is more suitable for the purposes of understanding the sources of coder disagreements and further improving the guideline, here, we report κ scores under a frame-based analysis. The reason for this is that in certain situations, the event-based approach is arguably too punitive, leading to sometimes much deflated agreement scores that do not seem to be an accurate reflection of the situation. To give an example that is also discussed in the extended report, imagine that one annotator coded an entire sentence as involving a downward head position (*down*) on the ‘NMM.head-y’ tier, while a second annotator broke up the same unit into three separate long *down* segments, with two intervening short *neutral* segments. Under the event-based method, *all* events coded on this tier would be analyzed as ‘unmatched’, thus heavily impacting the inter-annotator agreement score. Under the frame-based method, only the two short segments labeled *neutral* by the second annotator would be labeled as disagreements. Moreover, we observe that labels that are not so frequently used (such as *down* on the ‘NMM.lip-corners’ tier) generally yield much lower agreement scores under an event-based than under a frame-based method. For the purposes of the current paper, we therefore consider reporting the κ-scores under the frame-based method the most appropriate.

With regard to the label selection, firstly, for the analysis of sentence-structure patterns (section *Results: Polar question structures and their contexts of use*), it was important to consider non-manual polarity marking in our dataset. The primary NMMs for marking polarity are *nodding* (positive polarity) and *shaking* (negative polarity) on the ‘NMM.head-move’ tier; we therefore report the inter-annotator agreement scores for these labels in the table. However, we do not report the κ-scores for the labels *nod* and *shake*, which were used for single rather than repeated head nods and headshakes. While single head nods can be used as polarity markers (see (9-b) for an example), they can also have other linguistic functions. We therefore decided to exclude single head nods from the analysis altogether. The label *shake* was hardly ever used, as headshakes typically tend to involve multiple side-to-side head movements in NGT. Indeed, at least in a negating function, headshakes in NGT are known to be prone to spread over larger parts of the sentence [12,40].

Secondly, unlike the other tiers, the ‘NMM.eye-shape’ tier included ‘degree’ labels: *squint-full* vs. *squint-half*, and *wide-full* and *wide-half*. It turned out that the ‘half’ labels were more difficult to reach agreement on between the two annotators, as is also reflected by the lower inter-annotator agreement scores (0.45 for *squint-half* and 0.32 for *wide-half*). In cases of disagreement, when one of the coders had selected the ‘full’ label, the other coder had typically selected the corresponding ‘half’ label. In other words, coders agreed on the NMM itself but not on the degree to which it was engaged. But the pattern does not hold in the opposite direction: in cases where one coder had selected the ‘half’ label and the other coder disagreed, the nature of the disagreement varied: sometimes the other coder had selected the ‘full’ label and sometimes the *neutral* label. Given this, the analysis of NMM patterns in our dataset presented in the section *Results: Nonmanuals* is based on the annotation labels *squint-full* and *wide-full* only, which is the more conservative approach. Similar reasons also partially motivate our decision to exclude the labels *raised-low* and *inner-raised* on the ‘NMM.eyebrows’ tier from the analysis in the section *Results: Nonmanuals*. Both these labels received pretty low agreement scores (0.33 and 0.18, respectively), and the label *inner.raise* in particular was also applied relatively infrequently. A more theoretically-motivated reason not to include these labels in our analysis is that the literature on question forms in sign languages typically identifies specifically brow lowering and (supposedly full) brow raising as non-manual interrogative markers.

Returning to Table 7, overall, inter-annotator agreement for all but one of the NMMs that play a role in the discussions in this paper is quite decent, reaching moderate (0.41–0.60) or substantial (0.61–0.80) and in one case even almost perfect (0.81+) agreement. The exception is the label *forward* on the ‘NMM.head-z’ tier, which got a κ-score of 0.38, demonstrating only fair (0.21–0.40) agreement. This relatively low score can in large part be explained by mismatches due to annotators not agreeing on whether or not the label *forward* should apply (i.e., cases where one of the coders selected *forward* and the other *neutral*). Additionally, in the cases where both coders had identified a *forward* event, they sometimes disagreed on the onset and offset, further impacting the overall reliability score. We believe that the fact that annotations had to be made based on a single 2D camera view – and lacking a side-view camera perspective – made it difficult for annotators to reliably code head position. Indeed, not just the agreement scores for the ‘NMM.head-z’ tier were low but also for the ‘NMM.head-y’ tier with the labels *up* and *down*. As such, we expect that the addition of a side-view camera would increase reliability scores. For the present study, this issue cannot be overcome; hence, the discussion on the inquisitive function of forward head (and body) position in the section *What is the main non-manual marker of polar questions / inquisitiveness* should be appreciated with this relatively low inter-annotator agreement score in mind.

After we evaluated inter-rater reliability of the 20% of the dataset that was annotated by the two coders, 100% of the data was annotated for NMMs by one master coder, the first author of the paper.

### Annotating additional elements of interest

Finally, every annotation file included three additional tiers that could be used for annotating further elements of interest. Instances of palm-up, a multifunctional marker which can also function as a question particle in NGT [12,32], were annotated on the tier ‘MM.PU’. Other potentially interesting manual markers we wanted to keep track of were annotated on the tier ‘MM.sign’, while possibly relevant mouthings of Dutch spoken words accompanying manual signs were annotated on the tier ‘NMM.mouthing’.

We should note that we did not systematically analyze these additional elements of interest, which can be considered a limitation of the present study. Nevertheless, mouthings will be shown in the section *Or...* *tag* to play an important role in one particular type of question form.

## Results: Polar question structures and their contexts of use

Roughly speaking, we can distinguish three main types of question forms in our dataset in terms of their sentence structure.

1. SR: Questions consisting of a sentence radical only (N = 114);
2. SR-T1(-T2): Questions consisting of a sentence radical followed by one or two tags (N = 65);
3. EMB: Question forms involving embedding (N = 18).

**Fig 3:**
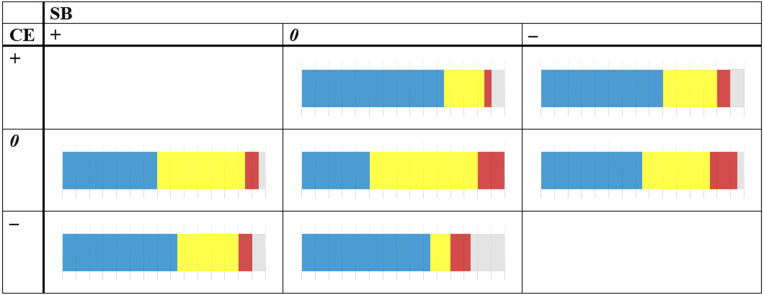
Frequency of occurrence of the main question types in each experimental condition. SR = blue; SR-T1(-T2) = yellow; EMB = red; EXCL = light gray.

Fig 3 gives an overview of the distribution of these main types of question forms across conditions. We can observe that instances of all three general sentence types are found in every condition. Moreover, conditions with neutral CE have relatively low counts of question forms consisting of a SR only, while forms containing a SR plus tag(s) and, to a lesser extent, forms involving embedding, are fairly common. Tags are also quite frequent in the contrastive conditions (PosNeg and NegPos). Question forms in conditions with positive or negative CE are more likely to consist of a SR only.

The following sections include examples from our data, which are glossed according to standard glossing conventions in the field. Sign glosses correspond to the ID-glosses in the NGT dataset published on Global Signbank (https://signbank.cls.ru.nl/datasets/NGT). We represent glosses in English except in the case of the signs wel (see this section) and toch-a/b (sure-a/b) (see the section *toch tag*). For clarity, the sign with the Signbank ID-gloss pu is glossed as palm-up in the examples in this paper. Non-manuals are indicated with abbreviations and a line above the manual glosses, which indicates their scope. The first part of each abbreviation signals which body part is engaged; the second part (after the period) indicates how the relevant body part is engaged. Glossary: ‘e’ = eyes [‘w’ = widened; ‘sq’ = squinted]; ‘br’ = (eye)brows [‘r’ = raised; ‘ir’ = inner brows raised; ‘l’ = lowered]; ‘h’ = head [‘s’ = shake/shaking; ‘n’ = nod/nodding; ‘fw’ = forward; ‘bw’ = backward; ‘d’ = down; ‘t’ = tilt]; ‘m’ = mouth [‘sh’ = shrug]; ‘l’ = lips [‘pr’ = pressed]; ‘n’ = nose [‘wr’ = wrinkle]; ‘b’ = body [‘fw’ = forward; ‘bw’ = backward; ‘sw’ = sideward]. The scope of these markers may differ, although they tend to spread over larger parts of the sentence.

In the next section, only non-manual *polarity* markers are glossed in the examples for reasons of simplicity; other non-manual (question) markers are not glossed but they can be observed in the corresponding videos which can be found at https://doi.org/10.21942/uva.21666203. From the section *Sentence radical and tag(s)* onward, all relevant non-manuals in linguistic examples are glossed as precisely as possible.

### Sentence radical only

A total of 114 question forms in our dataset consist of a SR only. We indicated for such constructions whether or not they ended with the sign palm-up, as this marker has been observed to occur frequently in question contexts in various sign languages (e.g., [32,43,44]). Thirteen constructions (11.4%) were found to include a prosodically integrated palm-up in sentence-final position. From this we can conclude that palm-up does not systematically take on a question-marking function in this construction type (but see the section *Sentence radical and tag(s)* on the use of palm-up in tags). In the remainder of this section, we will discuss question forms with and without palm-up together.

It is interesting to consider polarity marking in questions made up of just a SR. Of the 114 constructions, 38 did not show any overt polarity marking, 43 displayed positive polarity marking, and 33 showed negative polarity marking. Representative examples are given in (7): (7-a) does not include any manual or non-manual marking of polarity, while (7-b) and (7-c) are non-manually marked for positive and negative polarity by means of (repeated) head nodding and headshaking. (7-c) is also manually marked for negative polarity by means of the sign not-a.



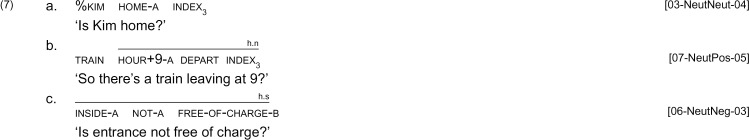



Table 8 shows the distributions of these different subtypes of question forms across experimental conditions. Darker shaded cells indicate that a particular type of polarity marking occurred in the *majority* of question forms within a condition.

**Table 8:** Distribution of question forms consisting of just a SR with different types of polarity marking.

No polarity marking					Positive polarity marking					Negative polarity marking			
	SB					SB					SB		
CE	*+*	*0*	*–*		CE	*+*	*0*	*–*		CE	*+*	*0*	*–*
*+*		4/21	8/18		*+*		17/21	10/18		*+*		-/21	-/18
*0*	8/14	7/10	8/15		*0*	6/14	3/10	3/15		*0*	-/14	-/10	4/15
*–*	2/17	1/20			*–*	4/17	1/20			*–*	11/17	18/20	

Darker shaded cells indicate that the majority of questions in the relevant experimental condition involve the type of polarity marking under consideration.

We can observe that the sort of polarity marking that is used tends to correspond with the polarity of the contextual evidence (CE) provided by the second confederate. A link with speaker bias (SB) as introduced by the first confederate is less clear. Still, the only question forms with negative polarity marking in the absence of negative CE were observed in the NegNeut condition, and ten out of the 17 question forms with positive polarity marking but without positive CE occurred in the two conditions with positive SB. For constructions without polarity marking, it is worth noting that a fair few of them (12/38) were uttered following positive CE. For comparison: none of the baseline questions consisting of a SR only (N = 14) involved overt polarity marking. Thus, polarity marking in the SR is clearly a hallmark of biased polar questions, where the type of polarity marking has the tendency to reflect the polarity of the provided CE.

Given that questions with negative polarity marking are rather common in our dataset, it is worth considering whether some of these question forms could be analyzed as high negation questions. Specifically, negative-polarity marked questions following positive SB (and neutral or negative CE) are interesting to consider here. As shown in Table 4, our data include a total of 11 question forms containing negative polarity marking that were uttered following positive SB and negative CE (and no cases following neutral CE). Inspection of these tokens reveals that about half of them cannot be high negation questions, as the non-manual marking patterns (specifically, eyebrow raising) and/or use of the mouthing ‘dus’ (‘so’) clearly show the signer prepared to accept the CE just provided by the second confederate. In other words, these questions clearly function to seek confirmation of the (negative) CE, making them incompatible with a high negation reading. The remaining cases have the potential to be high negation questions, although judgment data is needed in order to verify this. We provide an example in (8), where headshake is observed on the predicate. In this question form, the sentence structure itself does not provide any clues as to whether this might be a high negation question, but the non-manual marking pattern might (like in TİD and HKSL; see the section *Previous work on polar questions in sign languages*). The lowered eyebrows and squinted eyes could be interpreted as signaling that the signer has doubts about the CE provided by the second confederate (see the section *Factors associated with brow lowering* for more discussion), which is compatible with a high negation reading. However, it is also possible that these markers actually fulfill a different function – for instance, that the signer hopes the SR turns out to be false (again, see the section *Factors associated with brow lowering*) – which is compatible with a low negation reading of this question form. Further research is needed in this area.



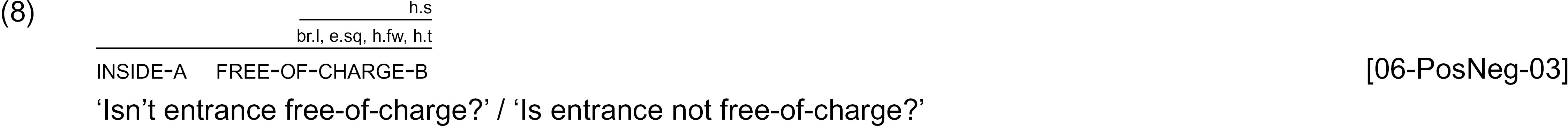



Finally, some question forms (either consisting of just a SR or a SR followed by a tag) include specific manual particles within the SR that are interesting to briefly mention here. Eight question forms included within the SR a sign we gloss as toch-a or toch-b (after the spoken Dutch particle ‘toch’); three forms included a sign we gloss as wel (after the spoken Dutch particle ‘wel’), and three forms included the sign hesitate-a. Both toch and wel can be analyzed as manual positive polarity markers. We provide an example for each type of particle in (9) below, where the English translation represents the closest approximation of the resulting meaning of the sentence the particle appears in. Interestingly, as will be shown in the section *Sentence radical and tag(s)*, all three particles illustrated in (9) can also occur as a tag following the SR.



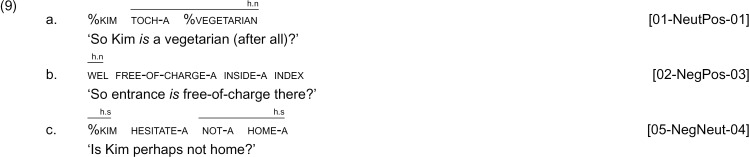



### Sentence radical and tag(s)

A total of 65 constructions consist of a SR and one (N = 63) or two (N = 2) tags. Tags are particular combinations of manual and non-manual markers that follow the SR, making up a separate prosodic unit. Constructions with tags typically display a marked shift in non-manuals between the SR and the tag, with usually multiple non-manual markers that are actively engaged in the SR changing their configuration in the tag. Sometimes, there is a small pause between the two sentence parts. We can distinguish five types of tags in our data, which we discuss below. Some of these tags were first described in [45].

#### Hesitation tag.

The most frequently occurring tag is what we will refer to as a hesitation tag (annotated as ‘HES’, N = 39). The precise realization of this tag may differ, but it always involves a combination of manual and non-manual markers, which together appear to signal hesitation or uncertainty about the truth of the SR on the part of the signer. Manual signs that may be used in a hesitation tag include palm-up, hesitate, and what-c (see https://signbank.cls.ru.nl/datasets/NGT for video illustrations of the signs corresponding to these glosses). In each of these signs, the palms face upward and the fingers of both hands are either fully extended or slightly curved, however the movement differs slightly. Non-manual markers that we observed frequently in hesitation tags include a forward body and/or head movement and a mouth configuration with the lip corners down (‘mouth shrug’); see the section *Results: Nonmanuals* for more in-depth discussion. Moreover, we often observed a headshake on the hesitation tag that clearly did not function to express negation. Headshakes are known to fulfill multiple functions beyond negation both in co-speech gesture [46,47] and sign languages [47–49]. In the cases we describe here, we argue that the headshake expresses uncertainty and possibly a request for a response from the addressee. We refer to such cases as *inquisitive headshakes*. We first described this function of headshake in NGT polar questions in [45], in which we analyzed a subset of the dataset discussed in the article; specifically, the 68 single-clause question forms that contain a headshake.

**Fig 4:**
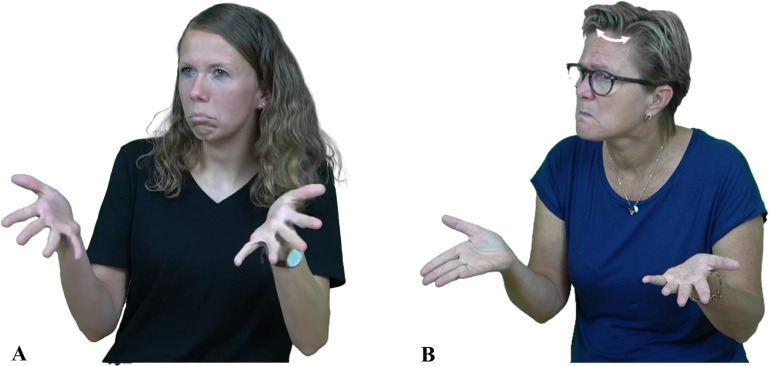
Two examples of hesitation tags. A: Hesitation tag as articulated in (10-a). B: Hesitation tag as articulated in (10-b).

Fig 4 shows two video stills illustrating hesitation tags; the corresponding glossed examples are provided in (10). In both examples, there is a marked change in non-manuals between the SR and the tag. Example (10-b) also includes an inquisitive headshake on the tag. In fact, there are two headshakes in (10-b): one on the SR and one on the tag, separated by a brief interruption. The headshake on the SR appears to have the dual function of expressing negation *and* hesitation. The amplitude (the head’s degree of rotation from side to side) of the second, inquisitive, headshake is smaller than the first, making the headshake less visually salient.



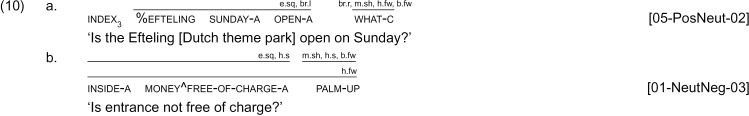



Table 9 displays the distribution of the hesitation tag across experimental conditions. The tag is quite common across all experimental conditions, but in particular when there is neutral CE. An inquisitive headshake occurs in about half of all the hesitation tags (N = 19/39) and is fairly evenly distributed across experimental conditions.

**Table 9:** Distribution of question forms with a hesitation tag.

	SB		
CE	*+*	*0*	*–*
*+*		5/28	4/28
*0*	6/29	10/30	6/29
*–*	5/28	3/25	

Hesitation tags may follow SRs with any type of polarity marking. There seems to be an interaction between the presence (or rather, absence) of an inquisitive headshake and the type of polarity marking. Of the 19 constructions *with* inquisitive headshake, positive polarity marking, negative polarity marking, and absence of polarity marking on the SR occur in approximately equally many cases (six, six, and seven cases, respectively). In contrast, of the 20 constructions with a hesitation tag but *without* inquisitive headshake, 15 involve a SR without overt polarity marking. Four cases involve positive polarity marking, and just one case involves negative polarity marking on the SR.

#### toch tag.

The second type of tag we discuss is what we refer to as the toch tag. In eleven constructions, the SR was followed by the sign toch-a (English gloss in Signbank: sure-a; two-handed sign) or toch-b (sure-b; one-handed); in two of these cases, the tag was followed by a second tag. The English ID-gloss sure stems from the fact that the sign toch can (also) be used to mean ‘certain(ly)’.

To understand the meaning of this particle, it is useful to discuss its apparent equivalent in Dutch, which has previously been investigated in various studies. Dutch ‘toch’ is a modal particle, used in biased polar questions, that may occur within the sentence or sentence-finally [50,51]. In sentence-final position, ‘toch’ roughly translates as ‘right’, as in, e.g., “Kim is a vegetarian, right?”. In sentence-medial position, ‘toch’ can have different functions, where there is also an interaction with word stress [52]. When stressed, ‘toch’ may function as a contrastive marker of concession, or to indicate inconsistency with the common ground, in which case it can be translated as ‘still’ or ‘after all’. When stress lies elsewhere, ‘toch’ typically functions as a reminder of the common ground, in which case its meaning approximates ‘surely’ [52]. On the face of it, the two variants of the NGT sign seem to function equivalently to Dutch ‘toch’, although their precise semantic and distributional properties require further investigation. The sign toch-a/b occurs both within the SR (see (9-a) and (13) for two examples of sentence-medial toch with different meanings) as well as sentence-finally, as a tag, in our NGT data. Here, we focus on the instances in which toch-a/b functions as a tag.

**Fig 5:**
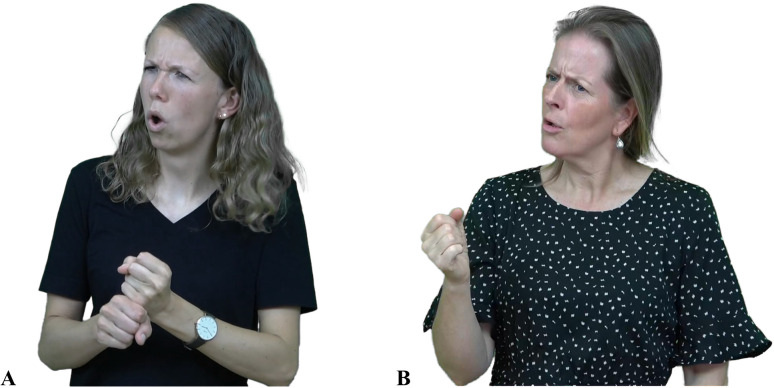
Two examples of toch tags. A: TOCH tag as articulated in (11-a). B: TOCH tag as articulated in (11-b).

Our data show that constructions with a toch tag typically involve eyebrow lowering and/or eye squint on both the SR and the tag. A head forward position is also very common, while eyebrow raise and mouth shrug are typically not observed. As such, sentences with a toch tag altogether involve a less pronounced shift in non-manuals than the other tag sentence types described in this section. Two representative examples from our data are illustrated in Fig 5 and glossed in (11).



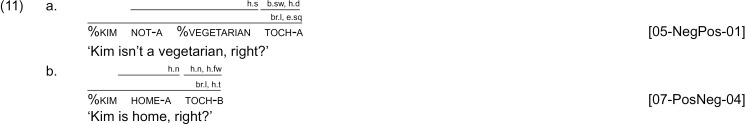



Distributionally, toch tags occur in conditions with non-neutral SB (Table 10). In all but two cases, polarity marking in the SR conforms to the polarity of the SB; that is, in the case of negative SB, there is negative polarity marking in the SR (as in (11-a)), and in the case of positive SB, there is positive polarity marking in the SR (as in (11-b)). The two exceptions both involve positive SB but no polarity marking on the sentence radical.

**Table 10:** Distribution of question forms with a toch tag.

	SB		
CE	*+*	*0*	*–*
*+*		-/28	3/28
*0*	4*/29	-/30	1/29
*–*	3/28	-/25	

*Two constructions in the PosNeut condition involve a double tag (toch + *or...*).

#### Or... tag.

**Fig 6:**
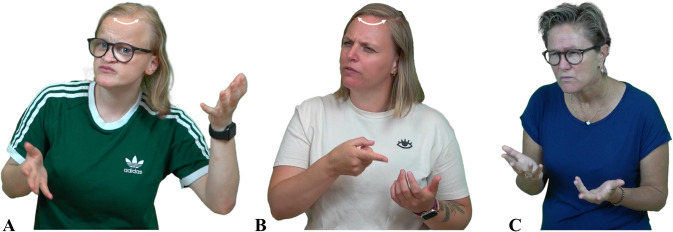
Three examples of *or...* tags. A: *Or...* tag as articulated in (12-a). B: *Or...* tag as articulated in (12-b). C: *Or...* tag as articulated in (12-c).

The third tag type we discuss is the *or...* tag, which occurred eleven times in our dataset. Examples of this tag type are illustrated in Fig 6 and are shown in context in the glossed examples in (12). In two of the eleven cases, the tag directly followed a toch tag; see (12-c) for an example. *Or...* tags look quite similar to hesitation tags, with the crucial difference that they involve a mouthing, which is either *of* (‘or’) or *of niet* (‘or not’). Like hesitation tags, *or...* tags typically involve manual signs that carry little lexical content but fulfill a more pragmatic or other (e.g., prosodic) function, such as hesitate-a (12-a), sentence-final pronoun copies (12-b), or palm-up (12-c). Regarding non-manual marking, the *or...* tag generally involves forward body and/or head movements, but headshakes and head nods also frequently occur. Headshakes may be used to express negation (‘or not’), uncertainty about the truth of the SR, inquisitiveness (a request for a response), or a combination. In example (12-a), for instance, we interpret the headshake on the tag as signaling uncertainty about the truth of the SR and/or inquisitiveness, while the headshake that is present in the preceding SR functions to mark negation. If the headshake (or head nod) on the *or...* tag functions to mark polarity, we find contrastive polarity marking on the SR. This is exemplified by the constructions in (12-b) and (12-c), where we can observe head nodding in the SR but headshaking on the tag, expressing negation (or not).



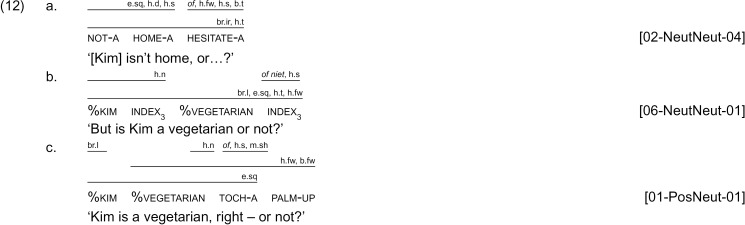



Distributionally, it is striking that all question forms with *or...* tags were uttered in contexts in which CE is neutral (Table 11). In cases where the tag was positive and followed a SR with negative polarity marking (12-a), we can furthermore observe that SB was either neutral or negative but not positive (Table 11, left), although note that the number of attested examples is very small here. In contrast, seven out of the eight sentences with a negative *or*... tag and positive polarity marking on the SR were attested in contexts with either positive or neutral SB (and neutral CE; Table 11, right). In other words, the polarity of the SR in constructions of this type tends to match SB.

**Table 11:** Distribution of constructions including an *or...* tag.

Negative polarity SR + *or*...				Positive polarity SR + *or*...			
	SB				SB		
CE	*+*	*0*	*–*	CE	*+*	*0*	*–*
*+*		-/28	-/28	*+*		-/28	-/28
*0*	-/29	1/30	2/29	*0*	4*/29	3/30	1/29
*–*	-/28	-/25		*–*	-/28	-/25	

#### Other tags.

**Fig 7:**
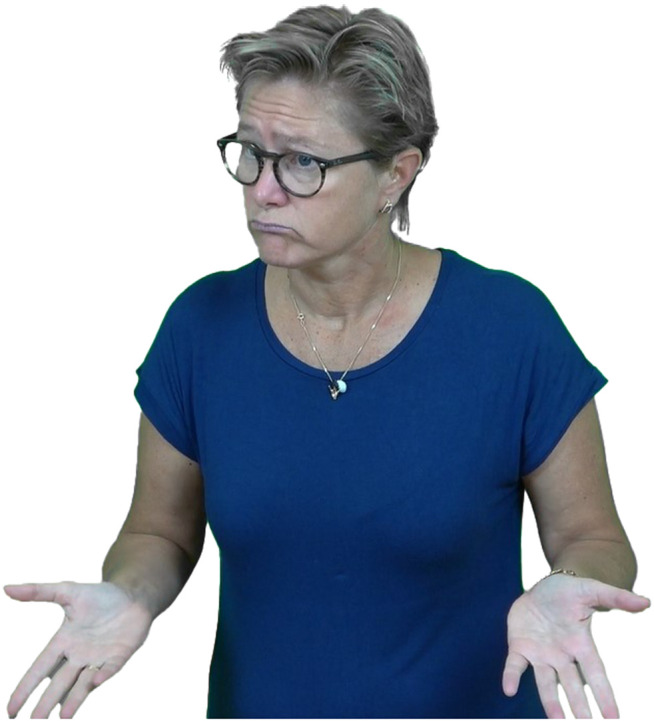
CONF tag as articulated in (13).

Finally, we found two other tag types in the data, which are exemplified by only a handful of tokens. Firstly, four elicited questions, produced by two different signers, included a tag type we gloss as CONF (for ‘confirmation’). The tag consists of a combination of palm-up or, in one instance, the sign wel, and a particular set of non-manuals. These include head nodding and raised (inner) eyebrows – both sustained from the SR – as well as pressed lips. The tag type occurred in three different conditions – NeutNeut (twice), NeutPos, and PosNeut – none of which involved negative SB or CE. We provide one example of the tag in Fig 7, with the corresponding example glossed in (13); also note the use of the sign toch-a in the SR.



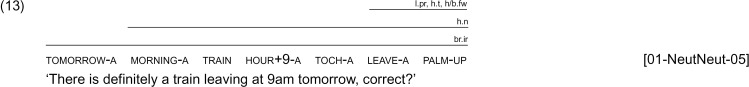



Interestingly, all four examples were uttered in the context of situation 5, in which participants had to ask a ticket seller at the train station about the availability of a 9am train to Paris the next day. Although we tried to control for differences in authority between the confederate roles as much as possible, this is the one scenario where the second confederate has a relatively high degree of authority. This tag type, and the non-manuals in particular, thus appears to function as a politeness strategy when asking a confirmation-seeking question.

**Fig 8:**
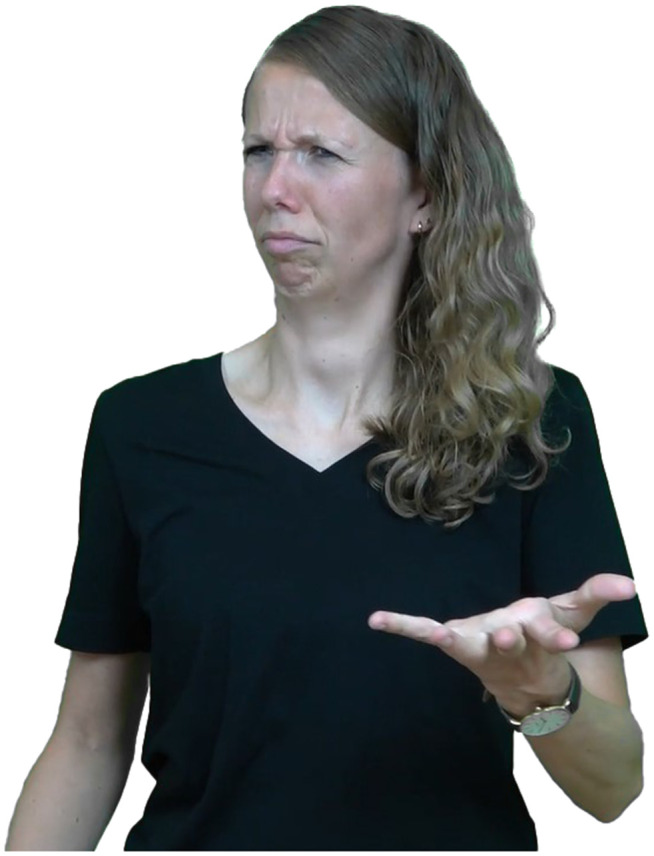
HUH tag as articulated in (14).

Finally, in two instances, both of which involved the same signer, the SR was followed by palm-up combined with non-manuals that seem to express a mixture of confusion and slight exasperation at the confederate’s previous response. As can be observed in Fig 8, these non-manuals include a backward head/body position, eye squint, brow lowering, and mouth shrug. We refer to this tag type as HUH. In contrast to all other tag types, the HUH tag involves backward rather than forward head and/or body movement. Both instances occurred in reaction to conflicting pieces of information offered by the two confederates (conditions PosNeg and NegPos). One example is given in (14). Various relevant non-manuals extend across the entire question form.



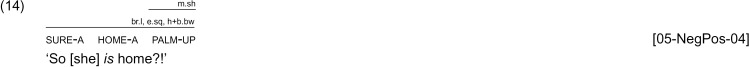



### Sentences with embedding

A total of 18 sentences in the dataset involve embedding, which we qualitatively describe here. The sentences can be divided into two categories based on the matrix clause predicate. In about two thirds of the examples (N = 11), the matrix clause includes the predicate know and the interlocutor is directly addressed (‘*Do you know...*’). In one of these cases, the predicate used is actually call-a, inflected for a first-person subject and a second-person object. The main clause can be roughly translated as ‘I want to ask you...’. Because we consider this a rough paraphrase of ‘do you know...’, we categorize it with the other tokens discussed here. In the remaining examples (N = 7), the matrix clause serves to introduce the signer’s own expectations about the matter of discussion (‘*I thought...*’; ‘*I heard that...*’). We discuss both construction types in greater detail below. The section will be concluded with a brief discussion about the distribution of the two types of embedded question forms across the five situations in the experiment.

#### Do you know....

The 11 question forms with the embedding predicate know come from four different signers. We observed two variations of this general construction type, illustrated by two representative examples in (15). A few examples also include a hesitation tag or an *or...* tag at the end of the sentence.



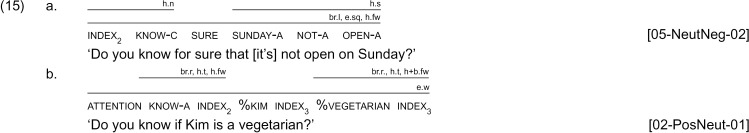



The two construction variations illustrated in (15) differ only in that the matrix clause in (15-a) additionally includes the sign glossed as sure. With the exception of the accompanying mouthing, this sign is formally identical to the sign used in toch tag constructions (see the section toch tag), but – as the mouthing (‘*ze(ker)*’; ‘sure’) indicates – the meaning is clearly different: the matrix clause roughly translates as ‘Are you certain that...”. This construction type was used twice, by the same signer. The utterance followed positive CE in one case, and negative CE in the other; in both instances, SB was neutral. In the case with negative CE, the polarity of the embedded SR mirrored the polarity of the CE; in the other case, polarity marking was neutral.

The other nine embedded clause constructions with know are of the type illustrated in (15-b), with a main clause that translates as ‘Do you know...’. In four cases, the main clause was reiterated at the end of the construction, which is a fairly common phenomenon in NGT. All nine tokens were uttered following neutral CE expressed by the second confederate. SB was positive in two cases, negative in three cases, and neutral in four cases. Thus, neutral CE appears to be a precondition for use of this construction type. This contrasts with ‘Do you know...’ constructions that also include the sign sure, which rather appear to require non-neutral CE (although the number of examples is, of course, low).

#### I thought....

Seven embedded clause constructions, uttered by three different signers, include a matrix clause that makes reference to the signer’s own expectations regarding the answer to the question at hand. Two representative examples, one with the predicate think and one with the predicate listen, are shown in (16).



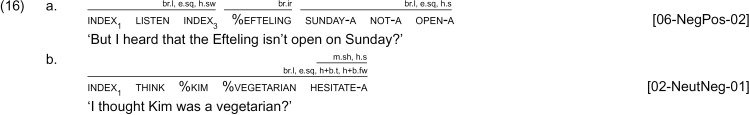



At first glance, the sentences in (16) may not have the appearance of interrogative constructions. However, the non-manuals that accompany the manual signs in these sentences, as well as the use of a hesitation tag in (16-b), indicate clearly that the signer is soliciting a response from the interlocutor. As such, we consider them to be questions both in function as well as in (primarily non-manual) form. Indeed, six of the seven constructions of the type discussed here include a hesitation tag. Of these seven constructions, four were uttered in one of the conditions in which the participant was presented with conflicting evidence from the two confederates (PosNeg or NegPos). Three of these instances involved the predicate hear/listen; the fourth case included the predicate think. The other three cases all involved think and were attested among the responses in the NegNeut (N = 1) and NeutNeg (N = 2) conditions.

#### Distribution of embedded question forms across situations.

Interestingly, 17 of the 18 interrogative constructions with embedding were uttered in the context of two of the five situations included in the experiment, namely situations 1 and 2 (see S1 File). Although the low number of examples prevents us from drawing strong conclusions from this, we may put forth a few testable generalizations by comparing the scripted exchanges with the second confederate that precede the target question in the five situations included in the experiment.

Regarding the 11 questions with the embedding predicate know, we think the disparity can be explained by the fact that the initial question the participant is instructed to ask the second confederate, and the confederate’s subsequent response, do not provide an obvious ‘hook’ for the target question. The participant may feel the need to signal this when posing their target question.

As for the complex constructions with listen or think, we hypothesize that the degree of familiarity between the signer and confederate roles may play a role, with politeness being an additional factor. In situations 1, 2, and 4, the confederates are presented as interlocutors who are known to the participant, which may make the participant more inclined to use a construction that centers on his/her own (unmet) expectations (“I thought...”), and less likely to go for a more polite question form. The reason we do not find the same type of construction in situation 4 may be that this situation involves a degree of urgency – the aversion of a small crisis hinges on a positive answer to the target question (“Is Kim home?”) – leading to more direct question forms being preferred by participants.

## Discussion: Polar question structures and their contexts of use

We now turn to a discussion of the main generalizations that can be drawn based on the results presented in the previous section. In each case, we compare the patterns found in NGT with related patterns in English (in which polar questions have been investigated most extensively in previous work) and/or Dutch (because NGT and Dutch are used in the same country, so there is a lot of contact between the two languages). Where possible, we also suggest possible explanations for the observed empirical generalizations.

### SR-only questions

The first generalization that can be drawn concerns the relationship between polarity marking on the SR of a polar question, on the one hand, and the type of contextual evidence that is available for the SR, on the other.

(17)  **Generalization**: In polar questions consisting only of an SR (i.e., without any tags) polarity marking correlates with the type of CE. More specifically:
  A. Questions with **negative** polarity marking (i.e., negation) mostly occurred in contexts with **negative** CE (29 out of 33 cases). The remaining four cases occurred in contexts with neutral CE and negative SB. CE was never positive.
  B. Questions with **non-negative** polarity marking (i.e., either positive polarity marking or no explicit polarity marking) mostly occurred in contexts where CE was **not negative** either (73 out of 81 cases).
  C. Questions with explicit **positive** polarity marking mostly occurred in contexts with **positive** CE (27 out of 43 cases) or **neutral** CE (12 out of 43). Of the remaining five cases, four occurred in contexts with negative CE but positive SB.

The first part of the generalization (A), concerning negative polarity marking, aligns with a generalization proposed by Büring and Gunlogson [16] for English: according to them, a polar question in English with low negation, i.e., a question of the form ¬p?, is felicitous only if there is compelling contextual evidence against ¬*p*?, i.e., in our terminology, if there is negative CE. Note that Büring and Gunlogson [16], like many other authors, make a distinction between *high* negation polar questions (e.g., *Isn’t there a vegetarian restaurant around here?*) and *low* negation polar questions (e.g., *Is there no vegetarian restaurant around here?*). Only the latter are relevant for our discussion here.

However, Van Rooij and Safárová [20] have argued that Büring and Gunlogson’s generalization for English is too strong. They point out that questions of the form ¬p? can sometimes be felicitous without any particular contextual evidence for or against *p*. One of their examples is given in (18).

(18) [A woman goes to the doctor with her son. The doctor asks the mother:]
  Has he not been eating properly?

Experimental evidence in line with this observation, i.e., evidence demonstrating that low negation polar questions in English can be felicitous in contexts without any particular contextual evidence for or against the SR, has been given in [18].

Turning back to NGT, our intuition is that a polar question with negative polarity marking on the SR would be felicitous in the equivalent of Van Rooij and Safárová’s example (18) in NGT as well. And indeed, our dataset also includes four instances of questions with negative polarity marking in contexts with neutral CE. So Büring and Gunlogson’s generalization seems to be too strong for NGT as well as for English.

For English, a weaker version of Büring and Gunlogson’s generalization has been proposed by Roelofsen and colleagues [53], who propose that a polar question with low negation of the form ¬p? in English is felicitous only if there is no compelling contextual evidence for *p*, i.e., in our terminology, if there is no positive CE. This weaker generalization for English is compatible with Van Rooij and Safárová’s observation and the experimental evidence in [18], and our data suggests that it also holds for NGT.

Before turning to a possible explanation of this pattern, let us consider part B of Generalization (17): our results indicate that in NGT, SR-only polar questions without negative polarity marking (i.e., either without any polarity marking at all or with positive polarity marking) mostly occur in contexts without negative CE. This pattern also aligns with what has been observed for English (see [16–18,53], among many others). For instance, the polar question in (19-b) is clearly infelicitous, while (19-a) is completely natural (example adapted from [16]).

(19) Scenario: A enters B’s windowless office wearing a dripping wet raincoat (so there is contextual evidence for it being rainy outside and against it being sunny). B asks:
  a. Is it raining outside?
  b. #Is it sunny outside?

Roelofsen et al. [53] formulate a generalization for polar questions in English that captures both cases with low negation and cases without negation in one go: namely, polar questions are felicitous only if there is no compelling contextual evidence that the SR is false. Our results suggest that this generalization also applies to NGT.

Finally, part C of Generalization (17) concerns polar questions in NGT with explicit *positive* polarity marking (in most cases head nodding). In this case, there is no clear correspondence with a polar question form in English (at least not one that has been investigated in depth so far). In particular, polar questions in English with an emphatic positive marker such as *really* (see, e.g., [19]) have rather different felicity conditions than polar questions with positive polarity marking in NGT. We leave open here whether there are any polar question forms in other languages, signed or spoken, which have the same felicity conditions as polar questions with explicit positive polarity marking in NGT. We are not aware that any such forms have been discussed in the literature, but of course this does not at all exclude the possibility that such forms do exist.

A possible explanation of the generalization that polar questions in English are felicitous only if there is no compelling contextual evidence that the SR is false has been proposed by Roelofsen et al. [53] in terms of a pragmatic principle which they term *Avoid Reverse*. This principle says that, other things being equal, when formulating a polar question, cooperative speakers formulate the question in such a way as to minimize the chance of eliciting a ‘reverse’ response from their interlocutor, where a ‘reverse’ response is one that rejects the SR of the question. This explains the generalization, because it disfavors the asking of a polar question if there is compelling contextual evidence that the SR is false. After all, in this case the question is very likely to elicit a ‘reverse’ response. Note that this explanation relies on a general pragmatic principle, not on anything specifically related to English. So, it plausibly applies beyond English as well, in particular to NGT.

### Questions with tags

We now turn to questions with tags. We will discuss hesitation tags, toch tags, and *or...* tags in turn.

#### Hesitation tags.

Polar questions with hesitation tags occurred quite frequently in our dataset, and they occurred across all experimental conditions (see Table 9 on p. 23). We hypothesize that hesitation tags express *uncertainty* on the part of the signer. This uncertainty could be of at least two kinds. First, it could be uncertainty about the truth of the SR. But, second, it could also be uncertainty as to whether the interlocutor will be able to resolve the issue expressed by the question – we will refer to this, in short, as uncertainty about *interlocutor knowledgeability*. Moreover, in the case of uncertainty about the SR, a further distinction can be made with respect to the *source* of the uncertainty: the signer could either have *too little* information to be sure about the truth of the SR, or alternatively, they could have *conflicting* information – in particular, when SB and CE contradict each other.

The mapping from experimental conditions to these different types of uncertainty is as follows. In the NeutNeut condition, the signer has no information whatsoever about the truth of the SR (no SB and no CE), so this is a condition in which the signer is likely to be uncertain about the SR due to a lack of information. On the other hand, in the PosNeg and the NegPos conditions, the signer has conflicting information. So in these two conditions, the signer is also particularly likely to be uncertain about the truth of the SR. Finally, in all conditions with neutral CE (that is, PosNeut, NeutNeut, and NegNeut), the signer could be uncertain about interlocutor knowledgeability, because the interlocutor has not yet provided any information which indicates that they will have an answer to the question. In conditions with positive or negative CE, this is not the case.

Based on our findings (see again Table 9), we provisionally conclude that hesitation tags can express uncertainty of all three types, since they occur in neutral CE conditions but also in conditions in which SB and CE contradict each other. In fact, they even occur in the two remaining conditions, NeutPos and NeutNeg. We suggest that in such cases, even though the interlocutor has already given an explicit hint about the truth of the SR, some uncertainty on the part of the signer still remains and is expressed by the hesitation tag. Note that hesitation tags are most frequent in the NeutNeut condition, in which the signer is likely to be uncertain *both* about the truth of the SR *and* about interlocutor knowledgeability. Further work is needed to establish what type(s) of uncertainty can be expressed by hesitation tags in a more systematic way, and whether such tags are articulated differently depending on the type of uncertainty that they are intended to convey.

#### toch tags.

Our results for questions with a toch tag give rise to the following generalization:

(20)  **Generalization**: toch tag questions are only felicitous if there is a prior signer belief that the SR is true.
  - toch tag questions of the form ‘*p*, toch?’ (without negation in the SR) only occurred in contexts with positive SB, i.e., a belief in *p*.
  - toch tag questions of the form ‘¬p?, toch?’ (with a negated SR) only occurred in contexts with negative SB, i.e., a belief in ¬p.

Gaasbeek [51] proposes that polar questions in Dutch with the tag ‘toch’ are subject to the same generalization. In English, questions with the tag ‘right’ also require that the speaker believes the SR to be true. It is likely that the NGT sign toch is influenced by Dutch, because the sign typically comes with mouthing ‘toch’.

#### *Or...* tags.

Our data give rise to the following generalization concerning questions with *or...* tags:

(21) **Generalization**: *Or...* tag questions are only felicitous if there is neutral CE.

Interestingly, one of the polar question forms in LSC discussed by Cañas Peña [28] has exactly these felicity conditions, namely those that include what Cañas Peña calls the yes-no Q-sign and lowered eyebrows. We are not aware of any polar question forms in Dutch, English, or other spoken languages with similar felicity conditions (which of course does not mean that such forms do not exist, as our knowledge of polar question forms across languages is inevitably limited). Note, in particular, that English *or not* questions, discussed by Biezma [54], have rather different felicity conditions. They have what Biezma calls a *cornering effect*, strongly forcing a yes/no answer from the addressee (rather than a more indecisive answer like ‘perhaps’).

## Results: Nonmanuals

In this section, we report on the non-manual marking patterns in the question forms in our dataset, specifically focusing on the distributional patterns in non-manual marker uses across experimental conditions. We made annotations for non-manual markers on twelve different tiers (see the section *Annotating non-manual markers* for details). However, we will discuss only a subset of these tiers (and labels) in this section. We selected non-manual labels that (i) occurred frequently in our dataset, (ii) yielded acceptable inter-annotator agreement scores, and (iii) we know from previous literature can play a role in question marking in sign languages. For instance, as mentioned before in the section *Previous work on polar questions in sign languages*, Zeshan [14] (p. 19) identified eyebrow raise, wide opened eyes, a forward head position and body posture, and eye contact with the addressee as typical markers of polar questions in sign languages, further noting that “non-manual signals marking polar questions tend to be very similar across signed languages”. Almost all these markers are considered in our analysis. The one exception is eye gaze, which we do not address even though we did annotate eye gaze direction. The picture is clear: signers virtually always look at the addressee when they ask a question, although we have no suitable data for comparison and thus we cannot conclude that sustained eye contact is a marker of polar questions, specifically. We did elicit baseline declarative sentences with a subset of our participants, but signers were asked to sign these sentences toward the camera rather than an interlocutor (which we annotated using the label *addressee*).

Thus, there are non-manual labels (e.g., *backward* on the ‘NMM.head-z’ tier) which are potentially of relevance but are not systematically analyzed here. The selected tiers and annotation labels are *raised* and *lowered* on the ‘NMM.eyebrows’ tier; *squint-full* and *wide-full* on the ‘NMM.eye-shape’ tier; *forward* on the ‘NMM.head-z’ tier; *forward* on the ‘NMM.body-position’ tier; *down* on the ‘NMM.lip-corners’ tier (see Table 7 in the section *Annotating non-manual markers* for the inter-annotator agreement scores for these labels). Note that the *κ*-score for the label *forward* on the ‘NMM.head-z’ tier was somewhat on the low side (0.38), so the results for this label should be interpreted with this in mind. The labels *nodding* and *shaking* on the ‘NMM.head-move’ tier, which are also listed in the table, will not be considered in this section, as they were already analyzed (as polarity markers/inquisitive headshakes) in the previous section on sentence structure.

More generally, not too much value should be attached to the *absolute* frequency counts presented in this section, given both the sometimes less-than-perfect agreement scores as well as the relatively small size of the dataset. Nevertheless, we believe it is still valuable to consider *relative* proportions, that is, we can compare the proportions of occurrence of particular non-manual markers to each other and across experimental conditions.

Two additional notes before we present the results. Firstly, in this section we only include in our analysis question forms that consist of (i) just a SR or (ii) a SR followed by a hesitation tag, leaving out of consideration constructions that include other tag types (as well as sentences with embedding). We have too few tokens for these latter construction types to perform any meaningful quantitative analysis on them. For some qualitative discussion on non-manual marking patterns in sentences with toch, *or...*, and other, less frequent, tags, see the relevant subsections in the section *Sentence radical and tag(s)*. Secondly, bear in mind that we do not take into account temporal information about the non-manuals under discussion in our quantitative analyses. That is, in our reporting of the proportions of occurrence of non-manuals, we count the non-manual whenever it is attested somewhere in the SR (or tag), without considering its onset or offset.

**Fig 9:**
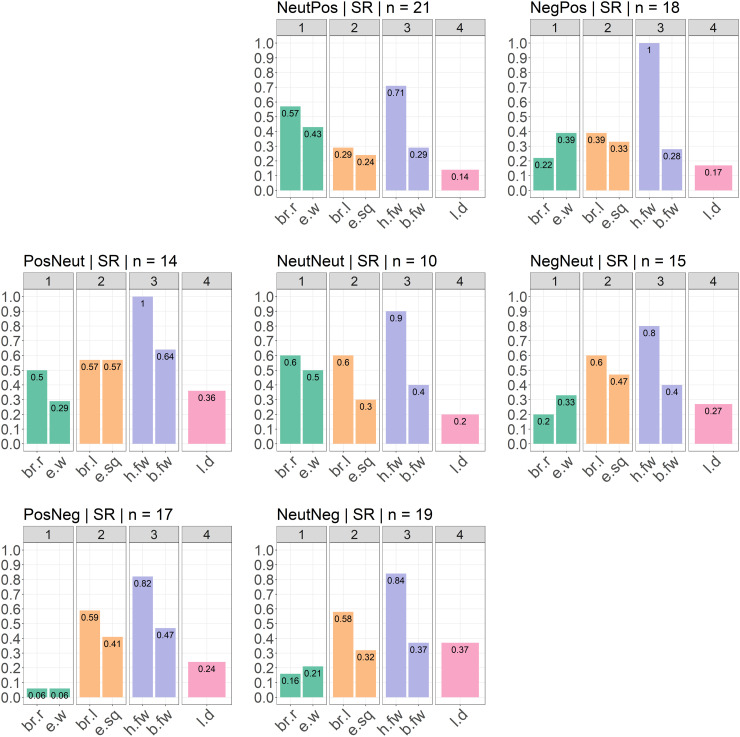
Proportions of non-manuals in question forms per experimental condition for questions consisting of just a SR. The number of tokens per condition, based on which the proportions are calculated, are reported in the subplot headers. Per condition, each plot consists of four subplots for different non-manual markers. Subplot 1 (green bars): raised eyebrows (‘br.r’) and widened eyes (‘e.w’); subplot 2 (orange bars): lowered eyebrows (‘br.l’) and squinted eyes (‘e.sq’); subplot 3 (purple bars): head forward (‘h.fw’) and body forward (‘b.fw’); subplot 4 (pink bars): lip corners down (‘l.d’; = mouth shrug).

**Fig 10:**
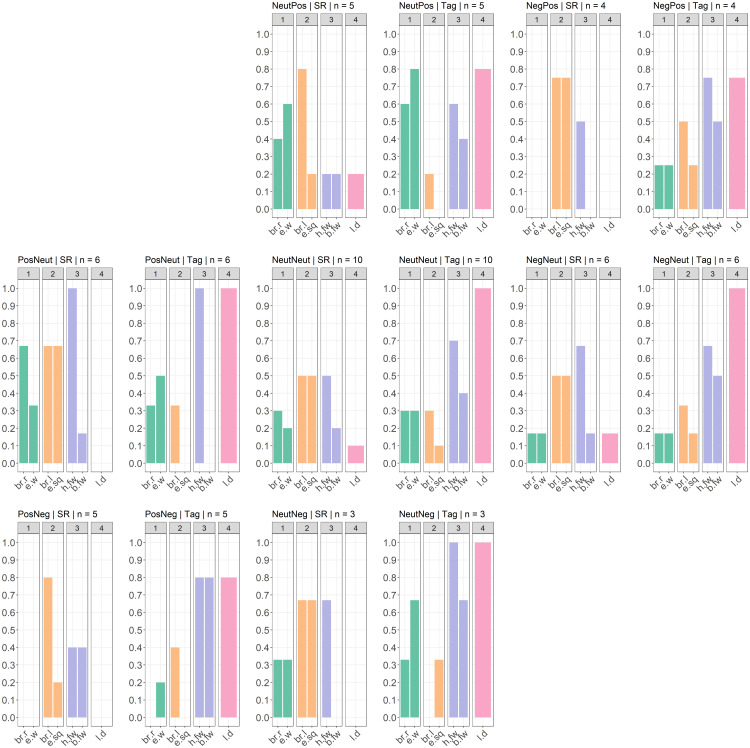
Proportions of non-manuals in question forms per experimental condition for questions consisting of a SR and a hesitation tag. The number of tokens per condition, based on which the proportions are calculated, are reported in the subplot headers. For each condition, proportions are represented for SR (left) and tag (right) separately, and both main plots consists of four subplots for different non-manual markers. Subplot 1 (green bars): raised eyebrows (‘br.r’) and widened eyes (‘e.w’); subplot 2 (orange bars): lowered eyebrows (‘br.l’) and squinted eyes (‘e.sq’); subplot 3 (purple bars): head forward (‘h.fw’) and body forward (‘b.fw’); subplot 4 (pink bars): lip corners down (‘l.d’; = mouth shrug).

Fig 9 shows the proportions of occurrence of all non-manuals under consideration in constructions consisting of just a SR for each experimental condition separately. Fig 10 shows the patterns for constructions made up of a SR and a hesitation tag, where the proportions are calculated and shown separately for each part of the question form.

### Head/body forward

In simple polar questions without a tag, ‘head forward’ is the most frequently used non-manual marker. The label *forward* on the ‘NMM.head-z’ tier was used in over 80% of SR-only polar questions in all experimental conditions except in NeutPos. In this condition, the proportion of question forms with head forward is slightly lower (0.71), but it is still the most commonly used non-manual marker. A comparable pattern can be observed in constructions ending with a hesitation tag, where it can additionally be noted that ‘head forward’ occurs more frequently on the tag than on the SR. Across all conditions and both question types, ‘body forward’ tends to occur less frequently, and in almost all questions in which a body forward position was observed, a head forward position occurred as well (but not necessarily vice versa).

### Eyebrow configuration and eye shape

Both eyebrow raising / eyes wide (green bars) and eyebrow lowering / eye squint (orange bars) are attested in all experimental conditions and for both question types under consideration (SR-only and SR + HES). However, there is variation between conditions with respect to the relative distribution of these markers. Brow raising and lowering sometimes occur in approximately equal amounts (e.g., PosNeut; NeutNeut), while in some conditions there is a preference for brow raising (NeutPos) or – more commonly – brow lowering (e.g., NegNeut; PosNeg). The patterns for eye shape (*squint-full* vs. *wide-full*) by-and-large mirror these patterns for brow configuration, with a general trend toward slightly lower proportions for these markers. This may be the result of the fact that the annotation labels *wide-half* and *squint-half* were not included in the analysis, because of the poor inter-annotator agreement scores for these labels. In other words, it is likely that we are looking at an underrepresentation of eyes wide/eye squint events compared to brow raising/lowering events.

### Mouth shrug

For SR-only questions, in all experimental conditions we find at least some uses of what we may refer to as a ‘mouth shrug’ (annotated as *down* on the ‘NMM.lip-corners’ tier). Proportions range between 14% and 37% across conditions. Strikingly, the use of mouth shrug is much more common in constructions made up of a SR and a hesitation tag, where this non-manual marker almost always accompanies the tag.

## Discussion: Nonmanuals

The results reported in the previous section reveal that eyebrow configuration and eye shape are highly variable in the polar questions in our dataset, while a head and/or body forward position occurs much more consistently. This is in line with observations we made previously in [45]. This suggests that ‘forward’ is the main polar question marker, or perhaps more generally, the main marker of inquisitiveness in NGT; an insight we discuss in more depth in the section *What is the main non-manual marker of polar questions / inquisitiveness*. In contrast, we found that eyebrow raise/eyes wide is much less frequently attested, and in some conditions even hardly attested at all, despite the fact that eyebrow raise is considered a typical polar question marker. In fact, brow lowering (and eye squint) is more common in our dataset overall. In the section *Factors associated with raised and lowered eyebrows*, we will argue that this is the result of the plurifunctionality of both brow raising and brow lowering, which leads to a competition between them in polar question forms, triggering different outcomes in different situations. Finally, mouth shrug also occurs in the polar questions in our dataset with quite some regularity, in particular when there is a hesitation tag. In the section *Function of mouth shrug*, we suggest that sentence-final mouth shrug expresses uncertainty in NGT, a function that it has also been argued to fulfill in several other languages.

### What is the main non-manual marker of polar questions / inquisitiveness?

We have seen that a head and/or body forward position is common across all experimental conditions. This leads us to argue in this section that a head/body forward position is the main marker of polar questions in NGT. Of course, this is not to say that a head/body forward position is *not* plurifunctional, as we argue is the case for eyebrow raising (and lowering), but we believe it is so to a lesser extent. Moreover, in contrast to what we observe for eyebrow raising vs. lowering, we do not find a competition between a forward vs. backward head or body position in NGT polar question forms.

**Fig 11:**
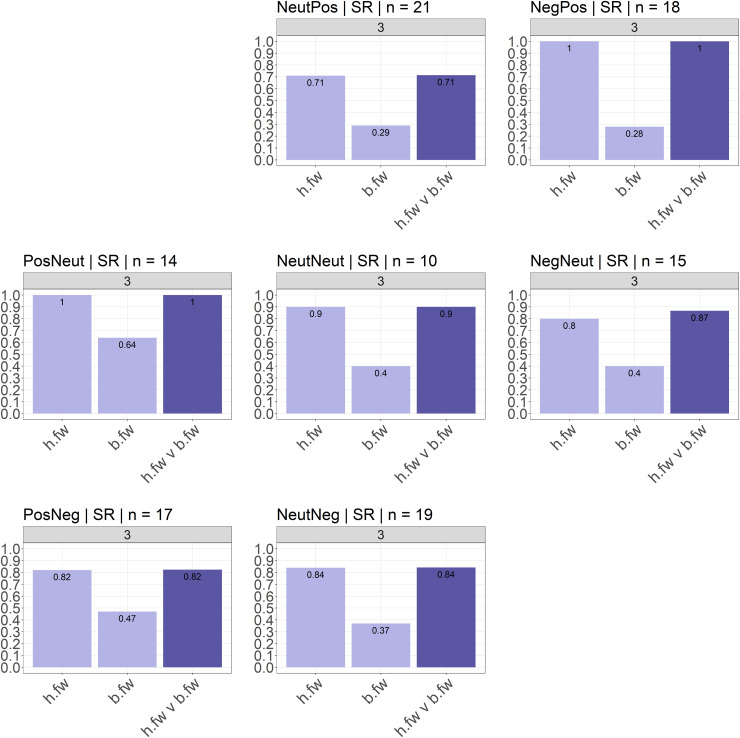
Proportions of head forward (‘h.fw’) and body forward (‘b.fw’) non-manuals in question forms per experimental condition, for questions consisting of just a SR. The third bar in each subplot indicates the proportion of question forms with either head forward or body forward, or both (indicated by the disjunction symbol ‘∨’). The number of tokens per condition based on which the proportions are calculated are reported in the upper right corner of each bar chart.

First, let us comment on the relationship between ‘head forward’ and ‘body forward’. Figs 11 (SR-only constructions) and 12 (SR + HES constructions) present the same information about the proportions of head/body forward per experimental condition as Figs 9-10 previously, but additionally include a third bar in each subplot which indicates the proportion of question forms that include a *forward* label on either the ‘NMM.head-z’ tier, the ‘NMM.body-position’ tier, or both. From this additional information, we can infer that when a particular question form has a *forward* annotation on the ‘NMM.body-position’ tier, it almost always has a *forward* annotation on the ‘NMM.head-z’ tier, too (but not necessarily vice versa), since in almost all experimental conditions, the third bar in the subplot is equal in length to that of the first bar (head forward). This suggests that head and body forward are likely expressions of the same underlying non-manual feature, which we will refer to as ‘forward’. The fact that we observe a forward head position more frequently than a forward body position is probably best explained by articulatory considerations: it is easier to only move the head forward than to move the body forward without also moving the head.

The proportion of ‘forward’ we observe in our dataset is in line with Coerts [12], who reported that 77.9% of the 95 polar questions in her NGT dataset include the feature ‘head forward’, making it the most common non-manual marker of polar questions in her data, too. In our dataset, 77.8% of SR + HES constructions involve a ‘forward’ feature on the tag (and 61.1% on the SR; see further discussion below). In SR-only polar questions, the proportion of instances involving a head and/or body forward position is even higher: 86.8%. We therefore suggest that a core function of ‘forward’ in NGT is to mark polar questions – or possibly *inquisitiveness* (i.e., a request for information from the interlocutor) more generally.

**Fig 12:**
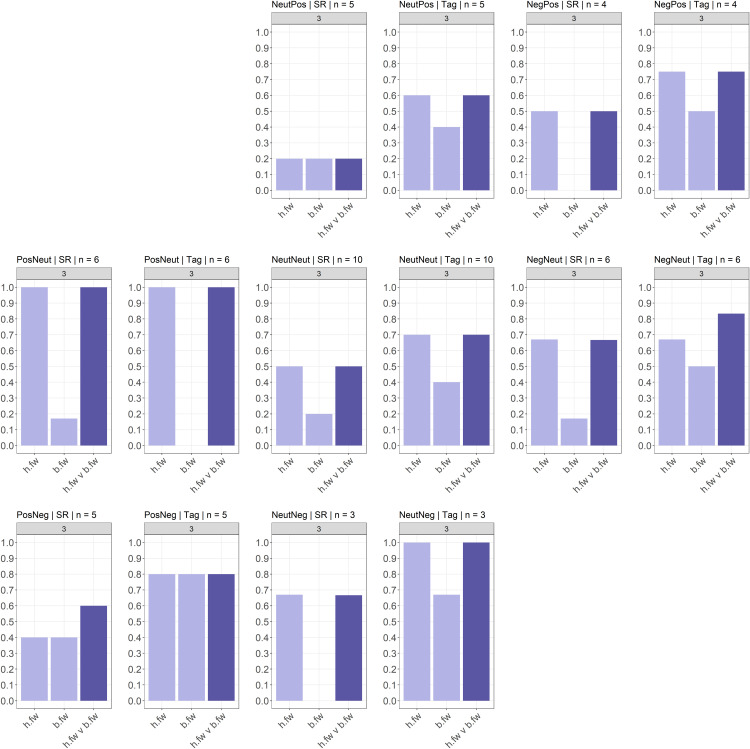
Proportions of head forward (‘h.fw’) and body forward (‘b.fw’) non-manuals in questions forms consisting of a SR and a hesitation tag, shown per experimental condition and separately for each part (SR or tag). The third bar in each subplot indicates the proportion of question forms with either head forward or body forward, or both (indicated by the disjunction symbol ‘∨’). The number of tokens per condition based on which the proportions are calculated are reported in the upper right corner of each bar chart.

Some support for this latter hypothesis comes from the temporal properties of this feature in our dataset. Based on qualitative inspection of the data, we observe that when ‘forward’ accompanies only a part of the question form, it is most likely to occur toward the end of it. Indeed, this observation is supported by Fig 12 for SR + HES constructions, where ‘forward’ accompanies the tag part at the end of SR + HES constructions at least as frequently as the SR part across all experimental conditions. This is significant, because this position – at the end of the signer’s turn – is expected under the assumption that ‘forward’ signals a request for information from the addressee.

However, if ‘forward’ is a general marker of inquisitiveness, it should not only occur frequently in polar questions but also in *content questions*. Coerts [12] reports that 70.6% of the content questions in her dataset (24 out of 34 in total) involve the feature ‘chin up’, of which seven are combined with ‘head forward’. She does not report an overall percentage for ‘head forward’ in content questions, which means that it must be less than the 50% that she takes as a lower limit for reporting features. Coerts’s work thus provides some indication that ‘forward’ is substantially less frequent in content questions than in polar questions. This would support the view that it is a marker of polar questions rather than of inquisitiveness more generally, though further data is required to verify this conclusion.

A possible alternative explanation is that the use of other markers of inquisitiveness in content questions (e.g., wh-signs) preempt the need for a forward position of the head and/or body. Indeed, in a recent corpus study on British Sign Language, Hodge et al. [55] show that wh-signs and wh-mouthings most strongly distinguish content questions from polar questions. Just 15% of polar questions were signaled manually by means of one or more question signs, as opposed to 76% of all content questions. However, the authors did not observe marked differences between polar and content questions with regard to how they are marked *non-manually*. Almost all question forms in their dataset were produced with at least one (and generally more) non-manual signals. Eyebrow movements and head movements occurred approximately equally often in both polar and content questions, although there were some distributional differences regarding the specific type of signals that were preferred. While almost all questions involved some form of eyebrow movement, polar questions involved proportionally more upward eyebrow movements, while content questions more often involved downward eyebrow movements. Still, raised and lowered eyebrows clearly occurred with both question types. With regard to head movements, 70% of polar questions and 58% of content questions included one or multiple head movements, where it is notable that polar questions involved proportionally many more head nods than content questions. Content questions, on the other hand, were more likely to include a downward head movement, and somewhat more likely to include a sideward head movement. Strikingly, and in contrast with the NGT data presented in this article, both question types involved a forward head movement relatively infrequently (15% for polar questions and 23% for content questions). Thus, it appears that ‘forward’ is not the main marker of polar questions in BSL, let alone of inquisitiveness more generally, while we have shown that it is a marker of polar questions in NGT (note that Hodge et al. [55] do not discuss body movements). At the same time, it is evident that – like in NGT – raised eyebrows are not a reliable marker of polar questions in BSL either.

It is further relevant to draw a connection with Göksel and Kelepir [56], who propose that ‘head forward’ is the main indicator of polar questions in Turkish Sign Language. In addition, they argue that ‘head tilt’, which for them encompasses both ‘head forward’ and ‘head backward’, functions to signal inquisitiveness (a “response seeking utterance”), while the direction of the tilt signals question type: a forward head tilt for polar questions and a backward head tilt for content questions. In NGT, one may similarly hypothesize that ‘forward’ and ‘chin up’ both articulate the feature *inquisitiveness*, the former being used when the issue that is raised concerns just two alternatives (yes/no) and the latter when there are multiple alternatives. Indeed, in a corpus-based study on wh-questions in NGT, Legeland [57] reports that ‘chin up’ was fairly common in her data; however, she did not annotate forward head/body positions. Thus, further study is needed here.

As an aside, Göksel and Kelepir also associate head nods and headshakes with polar and content questions, respectively. In NGT, we have seen that headshake can function as a marker of inquisitiveness and/or uncertainty about the truth of the SR, besides its function as marking negative polarity. Head nods do not seem to be involved in marking inquisitiveness in NGT.

A final connection relevant to draw here is with work on co-speech gesture which has found that head/body forward often occurs with inquisitive constructions in spoken languages as well, in particular in clarification questions [58–61]. It has been suggested that the relation between leaning forward and inquisitiveness potentially has a functional explanation: leaning forward reduces the space between interlocutors so it makes it easier for the speaker to pick up a response. For this reason, speakers may have a natural tendency to lean forward when asking questions. We should note that this explanation is more plausible for speech than for signing: in many contexts, moving closer to the interlocutor is likely to make it easier to hear what they say, but it will not necessarily make it easier to see their signed response.

Returning to the present study, if ‘forward’ indeed marks polar questions in NGT, then one may wonder why it is not attested in *all* polar question forms in our dataset. After all, the experimental design was such that participants were prompted to always request information from their interlocutors. We can think of at least three possible lines of explanation to account for this disparity.

Firstly, it is possible that a formally competing non-manual, like a backward head and/or body movement, blocks the use of ‘forward’ in some cases. One could imagine this happening, for instance, when a participant wishes to express strong surprise about or disagreement with the CE provided by the second confederate. However, our data do not offer much support for this explanation, as ‘forward’ is quite common in the contrastive conditions PosNeg and NegPos. Arguably, it is in these conditions that the likelihood that a signer will convey surprise or disagreement is the greatest. Possibly – and informal observation of the data supports this view – any competition between ‘forward’ and ‘backward’ may be resolved temporally, with the backward movement occurring earlier in the utterance, followed by a forward movement later on.

Secondly, it is possible that in some cases, participants may have felt a less pressing need to obtain information from the interlocutor because they already had some idea about the interlocutor’s answer. This could be the case in experimental conditions with positive or negative CE (some information already provided by the interlocutor), especially in combination with neutral SB (no conflicting prior information). The primary function of the target question may then subtly shift from a request for *information* to a request for *confirmation*, which could be less strongly associated with a forward head/body position. This explanation fits well with the observation that the proportion of SR-only constructions with ‘forward’ in the condition NeutPos is relatively low (0.71). However, it holds up less well against the fact that the proportion of SR-only question forms with ‘forward’ in NeutNeg is relatively high (0.84).

A third possible explanation is that the use of other markers related to inquisitiveness could make ‘forward’ superfluous. This may explain the difference between SR and SR + HES. A hesitation tag, as we will argue in the section *Function of mouth shrug*, signals uncertainty about the truth of the SR on the part of the signer, thus implicitly inviting a response from the addressee in order to resolve this uncertainty. This may obviate the need for using ‘forward’. Other potential markers that can make the use of ‘forward’ superfluous could be, for instance, prosodically integrated palm-up or inquisitive headshake on the SR.

Altogether, the data clearly show that ‘forward’ is the most consistently used non-manual marker in polar questions across experimental conditions. We have interpreted this as an indication that it is the main marker of polar questions, or possibly even inquisitiveness in general, rather than any particular eyebrow configuration. In the next section, we address the polyfunctionality of eyebrow raising and lowering.

### Factors associated with raised and lowered eyebrows

A striking pattern emerging from our data is that eyebrow raise is not as common as one might have expected given both previous work on NGT [10,12] and the fact that brow raising is widely regarded as prototypical of polar question marking across sign languages. Cecchetto [62], for instance, remarks in an overview chapter that “the importance of the eyebrow raise feature should be stressed, since it also discriminates polar questions from content (*wh*) questions in the many sign languages in which [...] content questions are marked by eyebrow lowering” (p. 294). Our data show that, at least in NGT, eyebrow raise is *not* a general marker of polar questions, underscoring previous experimental findings by De Vos et al. [33]. In fact, we found remarkably many instances of eyebrow *lowering* in our data.

Our general proposal, therefore, is that brow raising and lowering are both associated with a range of pragma-semantic factors that may or may not come into play depending on the context in which a polar question is uttered and the particular intentions that the signer wants to convey beyond just requesting a response that either affirms or denies the SR. Our objective in this section is to identify the factors we think play a role in order to account for the distributional patterns of eyebrow raising vs. lowering in our data. In the discussion, we will also make connections with relevant studies on both sign languages and co-speech gesture which suggest similar associations between particular brow configurations and the factors we discuss.

Before we begin our discussion, a couple of notes are in order. Firstly, because our primary aim here is to generate rather than test hypotheses about the functions of eyebrow raise and lowering, this section is of a more exploratory nature. Further study, e.g., in the form of a perception or acceptability judgment task, will be needed to gather additional empirical support for our suggestions. Secondly, we shall focus primarily on the set of question forms consisting of a SR only. Where possible or useful, we will also consider or compare constructions involving a SR followed by a tag, or the set of 30 baseline question sentences that we annotated. Thirdly, similar to what we observed for head forward and body forward, the occurrence of eyebrow raising and eyes wide as well as eyebrow lowering and eye squint are strongly correlated with one another, where the proportions for eye shape are typically slightly lower than the proportions for brow configuration. As such, it does not seem possible to associate one (but not the other) of the two markers in each pair with an independent function. In what follows, we will therefore use ‘eyebrow raising’ as a container term for brow raise and/or eyes wide, and ‘eyebrow lowering’ as a container term for brow lowering and/or eye squint.

**Fig 13:**
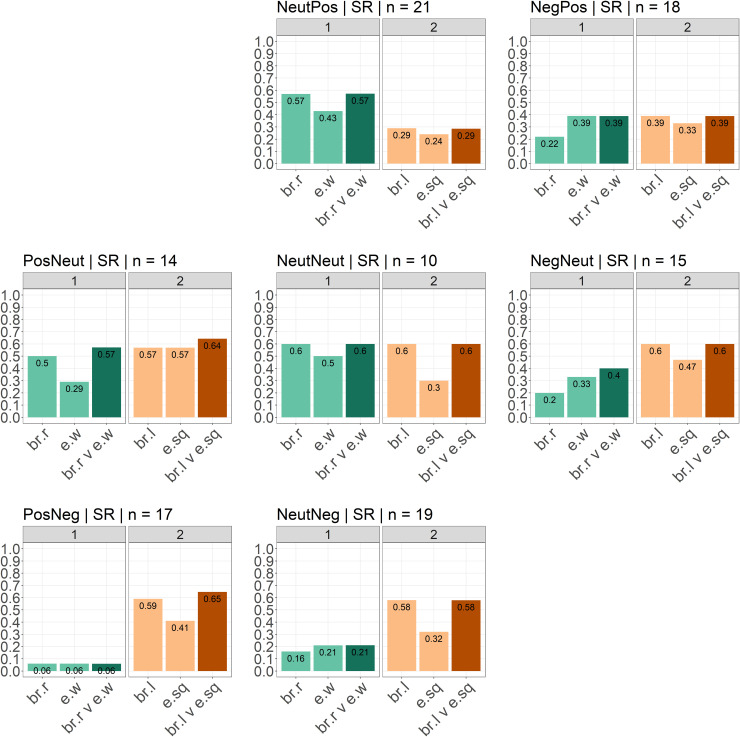
Proportions of brow position and eye shape non-manuals in question forms per experimental condition, for questions consisting of only a SR. Brow raising (‘br.r’) and eyes wide (‘e.w’) are displayed on the left in green, and brow lowering (‘br.l’) and eye squint (‘e.sq’) on the right in orange. The third bar in each subplot indicates the proportion of question forms with either brow raising/lowering or eye widening/squinting, or both (indicated by the disjunction symbol ‘∨’). The number of tokens per condition based on which the proportions are calculated are reported in the upper right corner of each bar chart.

To support the discussion, Fig 13 displays the distribution of eyebrow raising and lowering, respectively, in each experimental condition in the set of sentences consisting of a SR only.

**Fig 14:**
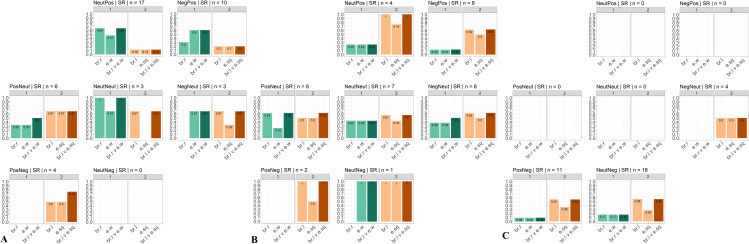
Proportions of brow raising vs. lowering in SR constructions with different polarity marking, for each experimental condition. A: Constructions with positive polarity marking. B: Constructions without polarity marking. C: Constructions with negative polarity marking.

Fig 14 shows the distribution of brow raise vs. brow lowering across experimental conditions separately for each type of polarity marking (positive, none, negative) on the SR. This figure reveals the following interesting three-way interactions between polarity marking, CE, and brow configuration:

1. When both CE and polarity marking on the SR are positive, brow raise is quite common while brow lowering is infrequent. The opposite pattern (brow raise infrequent; brow lowering frequent) is observed when CE is positive but there is no polarity marking.
2. This contrast between the forms with positive vs. no polarity marking is not observed when CE is neutral. There, we see that both eyebrow raising and lowering are quite frequent across conditions.
3. Finally, when there is negative polarity marking on the SR, brow raising is much less frequent than when there is positive or no polarity marking. On the other hand, brow lowering is still quite frequent when there is negative polarity marking.

Below we discuss a number of factors which we believe may be responsible for these three patterns.

#### Factors associated with brow raising.

We suggest that the following factors are associated with brow raising:

- Positive bouletic bias (the signer hopes that the SR is true)
- Seeking confirmation
- Introducing a new conversational topic
- Politeness
- Surprise

#### Bouletic bias.

First of all, one function that raised brows seem to fulfill is the marking of positive bouletic bias (cf. [20]), i.e., signaling that the signer *hopes* that the SR is true. A representative example is given in (22). In the given situation, the signer wants there to be a train at 9, so she hopes that the SR is true. The eyebrows are raised across the entire sentence.



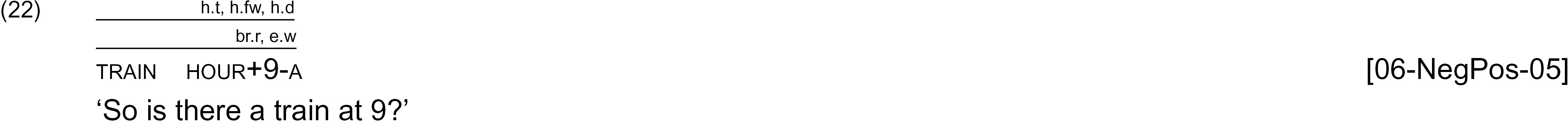



If eyebrow raise is indeed associated with positive bouletic bias, then we would expect to find differences in the occurrence of eyebrow raise across the five different situations in the production task. Situations 2–5 were such that the signer would naturally have a positive bouletic bias, hoping for an affirmative answer, in case they asked a question with non-negative polarity marking (that there be a train at 9, that entrance be free of charge, etc., see again S1 File for a description of all situations). Situation 1 is different: here, the signer cannot be assumed to have a positive (or negative) bouletic bias toward the SR (that Kim is a vegetarian). In setting up our experiment, we did not purposely control for bouletic bias. Unfortunately, we did not include any situation in which the signer would have a negative bouletic bias toward the SR when uttering a question with non-negative polarity marking (e.g., “So I need a[n expensive] ticket?”).

Now, of the 11 target questions consisting of only a SR with non-negative polarity marking that were elicited for situation 1, only one (9.1%) includes eyebrow raising. This compares to 51.4% eyebrow raises among the 70 SR-only questions with non-negative marking in situations 2–5 (for the plots, see Fig. A and Fig. B in S2 File). Of course, the number of question forms that these statistics are based on, especially for situation 1, is small, so any conclusions need to be drawn with caution at this stage. Nevertheless, these patterns are in line with what we would expect when positive bouletic bias is one of the factors associated with raised eyebrows.

Returning to the three main patterns observed based on Fig 14, the hypothesized association between positive bouletic bias (hoping that the SR is true) and brow raising can account in particular for pattern 3, i.e., the fact that brow raising is much less frequent when there is negative polarity marking on the SR than when there is positive or no polarity marking on the SR. This is because in case of positive or no polarity marking on the SR, we can assume that there is (in situations 2–5) a positive bouletic bias, and so we expect more brow raising. In contrast, if there is negative polarity marking on the SR, then we can assume that there is (again in situations 2–5) a negative bouletic bias (the signer hopes that the SR is false), and so we expect less brow raising. This is exactly what we see.

We are not aware of any previous studies on sign languages and/or co-speech gesture that make a similar connection between bouletic bias and eyebrow raise. However, the link between eyebrow raise and positive emotions such as hope are well-reported (e.g., [63]; see [64] for a recent multimodal study on participants’ use of eyebrow raises when talking about future hopes).

Bouletic bias cannot account for patterns 1 and 2, where we believe other factors are at play.

#### Seeking confirmation.

A second function that raised brows seem to fulfill in some cases, especially when combined with positive polarity marking (head nodding), is to signal a *request for confirmation* of the SR. This function is exemplified in (23), repeated from (7-b) with the addition of non-manual markers. The entire utterance is marked by raised eyebrows (plus widening of the eyes) and head nodding accompanies part of the sentence. The sense one gets when inspecting the utterance – as is also reflected in the English translation (‘So...’) – is that the signer is seeking confirmation of the proposition that the train leaves at 9. In this sense, the utterance differs from more neutral *information*-seeking polar questions.



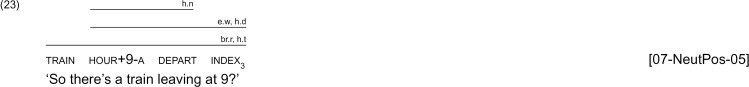



A number of previous studies on sign languages as well as co-speech gesture have also argued that eyebrow raising can be associated with requests for confirmation. Analyzing a set of dyadic conversations in spoken English between university students of different European nationalities, de Souza [65] reports a relatively high proportion of brow raises in confirmation-seeking polar questions as compared to information-seeking ones. Several other studies focus specifically on the use of eyebrow raising in other-initiated repair questions. For instance, in a qualitative study on Argentinian Sign Language, Manrique [66] reports that brow raise is used in ‘offer type’ other-initiated repairs, in which the signer offers their interlocutor a candidate understanding to request for confirmation. Similarly, in a corpus-based study on native Dutch speakers, Homke et al. [67] show that what they call ‘restricted offer’ other-initiated repair questions co-occur more often with eyebrow raises than eyebrow lowerings. And as mentioned before, Cañas Peña [28] claims for LSC that a combination of eyebrow raise with body/head forward in question forms may be used in contexts where epistemic bias (equivalent to SB) is positive and evidential bias (equivalent to CE) is not negative. Although implicit, it seems that she attributes a confirmation-seeking function to this non-manual pattern, as she notes that “[i]f the signer is expected to be answered with a negation, this combination of non-manual markers is not licensed” (p. 12).

Returning to the patterns observable in Fig 14, the association between seeking confirmation and brow raising in combination with positive polarity marking partly accounts for pattern 1, namely that the combination of raised brows and positive polarity marking is relatively frequent when CE is positive. The fact that there is positive CE in this case (which moreover has been provided by the interlocutor) makes it very likely that the answer to the question will be affirmative, therefore we expect more confirmation-seeking questions.

An interesting open issue is whether, when CE is *negative* rather than positive, raised brows can also be used in combination with *negative* polarity marking to signal that the question is confirmation-seeking. In our dataset, we hardly ever observe eyebrow raising in such cases. One conclusion we could draw from this is that brow raise is not associated with confirmation-seeking questions *in general* but only operates in tandem with positive polarity marking to signal confirmation-seeking in the presence of positive CE. There is, however, an alternative explanation. It could be that the broader association between brow raising and confirmation seeking does hold, but there are factors associated with brow lowering that weigh so heavily that brow lowering typically wins out over brow raising in questions with negative polarity marking – even if the question is confirmation-seeking (see the section *Factors associated with brow lowering* for further discussion).

It is important to highlight here that we are *not* suggesting that, when there is positive CE, a polar question should always be regarded as a confirmation-seeking one. Rather, we believe that this only happens if the person asking the question is willing to accept the positive CE. On the other hand, if she is not immediately willing to accept the positive CE, either because she has reason to doubt it (epistemic bias) or to disprefer it (bouletic bias) she would not seek confirmation but rather express her uncertainty/doubt or dispreference. In this case, we suggest, the signer does not use positive polarity marking on the SR. This would explain the contrast in Fig 14 between questions with positive polarity marking and questions without polarity marking in the presence of positive CE. In the former, raised brows are frequent and signal confirmation-seeking, while in the latter, lowered eyebrows are more common here. We will propose in the section *Factors associated with brow lowering* that lowered eyebrows, among other things, can signal uncertainty/doubt and negative bouletic bias.

#### Introducing a new conversational topic.

A third function that we suggest raised brows can fulfill is to signal that the signer introduces a *new conversational topic*. This would account for two of our findings. Firstly, it would partially account for the relatively high proportions of eyebrow raise in questions following neutral CE, independent of polarity marking (pattern 2). Recall that in our experiment, the information provided by the second confederate always followed a question by the participant which inquired about a different matter than the target question (e.g., ‘Do you know where the entrance is?’; target question: ‘Is entrance free of charge?’). In case of positive or negative CE, the confederate’s response indirectly offers evidence for (e.g., ‘It’s there by the white flag. You don’t need a ticket’) or against (e.g., ‘It’s there by the white flag, but you need to get a ticket first’) the SR. In contrast, in the neutral CE conditions, there is simply an absence of evidence concerning the SR (‘The entrance is there by the white flag’). This means that in the case of neutral CE, the signer’s target question opens up a new topic in the exchange between the participant and the second confederate. As such, these target questions are similar to out-of-the-blue polar questions, which we know from the literature are typically associated with raised eyebrows (e.g., [15], among many others). A representative example, uttered by a participant in situation 3 with both neutral SB and neutral CE, is shown in (24). Note that we analyze this example as a question form without polarity marking. The single head nod accompanying the lexical sign free-of-charge-a is analyzed as a prosodic boundary marker, a known function of single head nod in NGT [10].



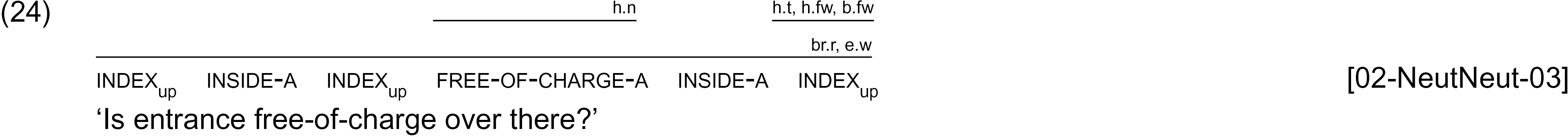



Thus, the suggested association between eyebrow raise and marking a new conversational topic contributes to the relatively high proportions of eyebrow raise in conditions with *neutral* CE. Secondly, it also predicts relatively high proportions of eyebrow raising in the baseline questions that participants were instructed to ask the first confederate in each role play. These questions also introduce a new conversational topic (rather than, e.g., asking for confirmation or clarification). Indeed, we find a similar proportion of eyebrow raising among these baseline questions (57%) as we observe among target questions without polarity marking following neutral CE.

A caveat here is that eyebrow lowering is very common in the set of baseline questions we annotated, too. This is surprising, because these questions, which participants were instructed to ask to the first confederate, introduce a new conversational topic. We suspect that the frequent use of eyebrow lowering here is an artifact of the experimental set-up: within the experimental context, the baseline questions were not uttered entirely out of the blue by participants, because each situation was repeated multiple times. It is possible that this unintentionally affected signers, leading to more frequent use of eyebrow lowering because they felt less of a need to signal that they were initiating a new conversational topic with each iteration.

Several previous studies on co-speech gesture have similarly identified an association between eyebrow raising and the introduction of a new conversational topic (e.g., [68–70]). We do not know of any such previous findings concerning sign languages, but it *has* been observed by many authors that brow raising is often used to mark grammatical topics (rather than new conversational topics) in sign languages, including in NGT [71].

The three functions of eyebrow raising discussed so far – signalling a positive bouletic bias, confirmation-seeking, and the introduction of a new conversational topic – together explain the three patterns found in Fig 14 concerning eyebrow raising. There are, however, two more relevant functions which we think eyebrow raising can fulfill in NGT, and which explain some of our data though this is not directly reflected by Fig 14. We now turn to these two additional functions: marking politeness and surprise.

#### Politeness.

We suggest that there is an association between eyebrow raising and politeness in NGT. We think that this factor particularly played a role in one of the five situations considered in our experimental setup, namely situation 5, in which the participants addressed a ticket seller at a train station. It is likely that the participants felt more pressure in this situation than in others, which were more informal, to formulate their questions in a polite way. This may have affected the data elicited in this situation in various ways. Firstly, as discussed previously in the section *Other tags*, all four instances of the confirmation-seeking tag CONF – non-manually characterized by a combination of head nodding, pressed lips, and raised (inner) eyebrows sustained from the SR – were elicited in this particular situation. We already suggested there that the CONF tag in combination with the use of these particular non-manuals signal politeness. Secondly, looking at all questions elicited in situation 5 that consist of a SR only (for the plot, see Fig. C in S2 File), we observe a relatively high proportion of eyebrow raising across conditions, while eyebrow lowering occurs in only a few instances – most of which following negative CE. Thus, we suggests that eyebrow raising functions to express politeness here. For a representative example, see (23).

Conversely, there is one situation in which eyebrow raising is relatively infrequent and eyebrow lowering is much more common. This is situation 4, in which the participant asks whether their roommate Kim is home to let them in. We suspect that the infrequent occurrence of eyebrow raising in this situation is in part due to the sense of urgency behind the target question (the participant would not be able to enter their home if it turned out their roommate was not there). As a consequence, and combined with the fact that the second confederate takes the role of another roommate, politeness considerations become peripheral, resulting in markedly less frequent use of eyebrow raise in this situation. An illustrative example is provided in (25); also note the use of nose wrinkle in this example.



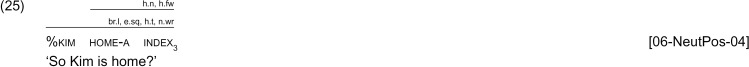



Some previous studies have also associated eyebrow raising with politeness marking. For instance, Mapson [72] reports for British Sign Language that eyebrow raise is the most consistently used non-manual feature by all participants in an experiment in which polite expressions (requests and apologies) were elicited. In a multimodal study, eyebrow raising was shown to positively influence participants’ perception of the politeness of verbal statements with speakers of American English, while eyebrow lowering had the opposite effect [73].

#### Surprise.

Finally, we propose that signaling *surprise* is also one of the functions that eyebrow raising can fulfill in NGT, though, importantly, only when we define *surprise* in a specific, rather narrow way. We will refer to this notion of surprise as *accepting, hopeful surprise*. An illustrative example is given in (26).



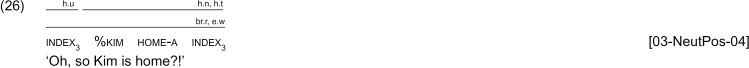



This example is an instance of ‘accepting’ surprise in the sense that the signer, being confronted with new, unexpected information, accepts this new information as true. It is also an instance of ‘hopeful’ surprise in the sense that the signer has a preference for the new, unexpected information to indeed be true. Another possible reaction to new, unexpected information, especially if the signer was previously told exactly the opposite, would be to enter into a state of uncertainty about the truth of this new information, to doubt it, or even to express their dismay. In that case, we may also say that the signer is surprised by the new information, but it is a case of ‘non-accepting’ surprise. This is more likely to be signaled by eyebrow *lowering*, as in example (27).



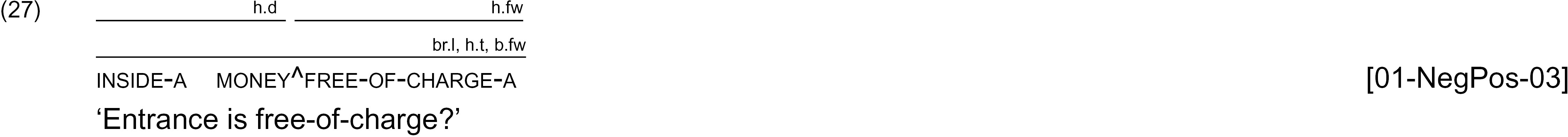



We have suggested before that, in case of positive CE, the way in which a signer marks her question depends on whether she is willing to accept this positive CE or not. If she is, she is likely to use eyebrow raise in combination with head nodding; if she is not, she is likely to use eyebrow lowering to signal uncertainty/doubt. Now, what we are suggesting is that in the first case, i.e., if the signer is willing to accept the positive CE – if she is furthermore surprised by this new positive CE because she had a different expectation (SB), and if she is hopeful that the new positive CE is indeed true, then she is even more likely to use eyebrow raising to signal ‘accepting, hopeful’ surprise. In Fig 14, this concerns the NegPos condition in the leftmost diagram (for question forms involving positive polarity marking on the SR). While the frequency of brow raising in this condition is not higher than in some other conditions, including NeutPos, we do believe that signaling surprise (of the relevant specific kind) is one of the factors promoting the use of brow raising in this condition, in addition to the other factors discussed above. Based on qualitative inspection of our data, it seems that if multiple factors including surprise-signaling independently promote the use of brow raising, this typically leads to brow raising with greater intensity. However, we did not annotate the intensity of non-manual markers in this study, and thus we cannot make any quantitative claims on a potential link between particular (combinations of) factors and the intensity of eyebrow raising or lowering. In future work, we think there is much potential for Computer Vision tools to be utilized for a more precise quantitative analysis than manual annotations can offer (see [74,75] for examples of such studies).

In previous work on sign languages, co-speech gesture, and the expression of emotions, eyebrow raising has often been associated with signaling surprise (see, e.g., [63]). The discussion above also resonates with previous experimental findings on NGT [33]. In this study, the authors show that polar questions in NGT uttered in a surprised affective state involved somewhat more frequent use of eyebrow raise compared to questions asked in a neutral affective state. Moreover, De Vos et al. [33] also report a higher *intensity* of brow raises for polar questions asked in a surprised affective state. Note here that De Vos et al. worked with a general, underspecified notion of surprise. They just asked their participants to imagine themselves in a ‘surprised’ affective state, not distinguishing between accepting/non-accepting or hopeful/fearful/neutral surprise. It is plausible, however, that participants imagined themselves in an accepting, hopeful surprise state, as this seems to be a relatively salient sub-category of surprise.

To conclude this section, we have put forth five factors which we believe promote the use of eyebrow raising in polar questions in NGT, and we have argued that each of these factors only comes into play under certain circumstances (e.g., depending on whether the signer wants the SR of the question to be true or not in that situation), in the presence of a certain type of CE, and/or in the presence of a certain type of polarity marking on the SR. Table 12 summarizes the discussion.

**Table 12:** Factors promoting the use of brow raising in polar questions in NGT.

Factor	Situation	CE	Polarity marking
*1. Positive bouletic bias*	2-5	positive; neutral	positive; no
*2. Seeking confirmation*	all	positive	positive
*3. Introducing a new conversational topic*	all	neutral	positive; no
*4. Politeness*	5 (and 4 opp.)	all	all
*5. Accepting, hopeful surprise*	2-5	positive	positive

#### Factors associated with brow lowering.

We suggest that three factors are associated with brow lowering in polar questions in NGT, of which negation and uncertainty about the SR were previously suggested as possible factors associated with brow lowering in [45]. Furthermore, some of the factors we identify are the direct opposites of the factors associated with brow raising that we discussed in the previous section.

- Negation
- Negative bouletic bias
- Uncertainty/doubt, of three subtypes:
  - Uncertainty/doubt about the SR
  - Uncertainty/doubt about the addressee’s ability to respond
  - Uncertainty/doubt about the contextual evidence

#### Negation.

Recall pattern 3 emerging from Fig 14: eyebrow lowering is overwhelmingly preferred over eyebrow raising in polar questions with negative polarity marking, to the extent that brow raising hardly occurs at all (also see [45]). One possible explanation for this pattern is that there is simply a general association between brow lowering and the expression of negation in NGT. This would be in line with Zeshan [76], who points out that eyebrow lowering and eye squint are non-manual markers that regularly occur in negative clauses across many sign languages. It also aligns well with Benitez et al. [77], who consider lowered eyebrows to be part of what they call the ‘not face’. They claim that this is a universal facial expression, used as a co-articulator in negative sentences in spoken languages and as a grammatical negative marker in American Sign Language. In this context, it is worth noting that for some sign languages, such as Turkish Sign Language, negation has (also) been associated with brow *raising* [78,79].

In light of this hypothesis, it is worth checking what happens in question forms that include an *or...* tag, specifically those where the SR is positive and the tag translates as ‘or not’, of which we had eight instances. Eyebrow lowering is indeed very common on these tags. We additionally observe that in some of these cases, lowered eyebrows are sustained from the positive SR (see, e.g., (12-b) in the section *Or... tag*). It is clear that lowered eyebrows must have a different function on that part of the sentence, since negation is not involved there. This serves as indication that negation cannot account for all instances of brow lowering in the dataset.

### Negative bouletic bias

The other factor we believe weighs into the preferred use of brow lowering over brow raising in polar questions with a negative SR is *negative bouletic bias*. The question in (28) illustrates this.



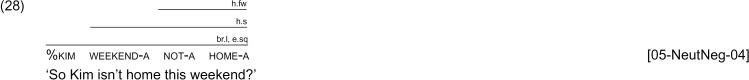



In the given situation, the participant wants Kim to be home, but the SR is the negative clause ‘Kim isn’t home.’ So, the participant has a negative bouletic bias: they hope that the SR turns out to be false.

In general, we suggest that the polarity of the signer’s bouletic bias, i.e., whether or not the signer hopes the SR to be true, is associated with contrasting brow configurations: brow raising signals a positive bouletic bias, while brow lowering signals a negative bouletic bias. This makes sense, because where positive bouletic bias may be associated with positive emotions like hope and happiness, which in turn are typically associated with eyebrow raise, negative bouletic bias can be connected to negative emotions like fear, sadness, anger and contempt, which are typically associated with eyebrow lowering (e.g., [63]).

Unfortunately, our current dataset does not allow us to determine the extent to which the expression of negation and negative bouletic bias independently promote the use of brow lowering, since in almost all cases in which a polar question with a negated SR is asked, there is also a negative bouletic bias (the signer hopes that the negated SR is false), and vice versa. This means that our data do not provide evidence as to what happens, for instance, in polar questions with a negated SR but positive bouletic bias (e.g., ‘So I don’t need to pay to get in?’). Our hypothesis is that both brow raising and brow lowering are possible in such cases, but brow lowering would only be used to signal uncertainty/doubt (a factor discussed below). In other cases, in particular in confirmation-seeking questions with a negated SR but positive bouletic bias, we expect that brow raising will be used. If this is indeed the case, negative bouletic bias would be a better predictor of brow lowering in polar questions than the presence of negation. However, further research is needed to settle this issue.

In any case, while the hypothesized associations between eyebrow lowering and negation and negative bouletic bias account straightforwardly for the fact that eyebrow lowering frequently occurs in polar questions with negative polarity marking in our dataset, eyebrow lowering is also rather common in cases where there is no polarity marking, as well as, to a lesser extent, positive polarity marking on the SR. These occurrences of brow lowering are not accounted for by the associations discussed so far. We propose that in these cases, eyebrow lowering generally has a different function, namely to signal uncertainty/doubt. We distinguish three types of uncertainty/doubt here: uncertainty about the truth of the SR, uncertainty about the interlocutor’s ability to answer the question (in short, about ‘addressee competence’, cf. [80]), and uncertainty about the CE that has been provided by the interlocutor right before the question is asked.

#### Uncertainty/doubt about the SR.

Uncertainty about the SR can be expected to play a particularly important role in polar questions that do not involve polarity marking, as in such cases it appears that the signer does not have a particularly strong bias toward either answer. Indeed, eyebrow lowering is rather common in questions without polarity marking across all experimental conditions. A representative example from the NeutNeut condition, where one could expect participants to be especially uncertain about the SR given the lack of contextual cues, is given in (29).



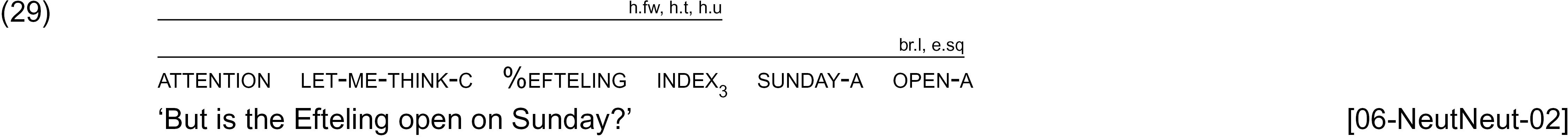



Of course, questions with hesitation tags (see the section *Hesitation tag*) are also interesting to consider in this context. In the section *Discussion: Polar question structures and their contexts of use*, we proposed that this type of tag functions as an uncertainty marker. We thus expect eyebrow lowering to commonly accompany this tag type, and as Fig. 10 shows, this turns out to be the case. See (30) for an example.



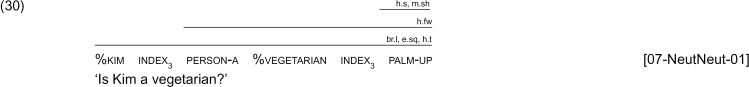



However, brow *raising* is also fairly common in questions with a hesitation tag. In part, this can be explained by the fact that some questions of this type involve both eyebrow raising and lowering in different parts of the sentence. In example (10-a) in the section *Hesitation tag*, for instance, there is brow lowering on the SR but brow raising on the tag. This suggests that hesitation tags and eyebrow lowering are not inextricably linked – in fact, brow raising is slightly more common on the tag than on the SR in questions with a hesitation tag. Perhaps the use of a hesitation tag obviates the need for brow lowering for the purposes of uncertainty marking, such that brow raising may be used instead, e.g., to signal that the question is confirmation-seeking.

Similarly, we may expect to see common use of brow lowering in questions that include an *or...* tag, as the use of such a tag may signal uncertainty on the part of the signer, for whom both answer options are still viable. We observe that brow lowering is indeed common across these questions, often accompanying both the SR and the tag (see, e.g., (12-b) in the section *Or...* *tag*). Eyebrow raising, on the other hand, is rarely attested.

#### Uncertainty/doubt about the interlocutor’s ability to respond.

Uncertainty about the interlocutor’s ability to respond (in short, about *addressee competence*) can be expected to surface in cases where the second confederate provided neutral CE. This may lead participants to suspect that the confederate might know the answer to the target question. If the first confederate provided positive or negative SB, then the participant might have a bias for one of the possible answers (resulting in less uncertainty about the SR), but they may nevertheless be uncertain about whether the second confederate can provide confirmation.

We indeed observe that eyebrow lowering is fairly common across conditions with neutral CE; however, other factors (such as the above-mentioned *uncertainty about the truth of the SR*) may also partially account for this pattern. As such, it becomes interesting to take another look at the questions with embedding of the *‘Do you know...’* type, which we discussed previously in the section *Do you know...*. Inspection of these eleven complex question forms shows that eyebrow lowering is indeed attested in such forms, as in (31), which was uttered in a context with negative SB and neutral CE (however, so is eyebrow raising; see (15-b) for an example). Since SB is not neutral, it seems plausible that eyebrow lowering, which accompanies the sentence-initial matrix clause and extends somewhat into the embedded clause, marks uncertainty about addressee competence rather than uncertainty about the SR. Moreover, the construction does not involve negation or negative bouletic bias. We also observe mouth shrug on the matrix clause (in the beginning of the utterance) as well as the matrix clause double (at the end of the utterance), which also appears to contribute to signaling uncertainty about whether the interlocutor knows the answer to the question expressed by the embedded clause.



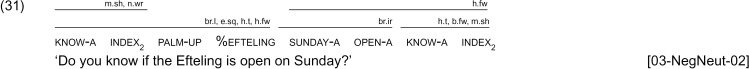



#### Uncertainty/doubt about the contextual evidence.

Recall from Fig 14 that eyebrow lowering is generally strongly preferred over eyebrow raising in the two contrastive conditions PosNeg and NegPos; an example is given in (32). Note that there is one notable exception, namely questions with positive polarity marking asked in a context with negative SB and positive CE. In such cases eyebrow raising is more common. We suggest that positive bouletic bias, confirmation-seeking, and (accepting, hopeful) surprise play a crucial role here, all promoting the use of eyebrow raising; see the section *Factors associated with brow raising*.



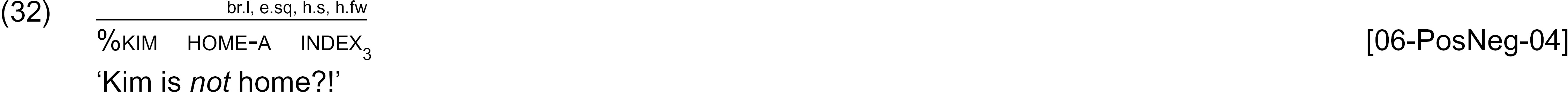



We suggest that uncertainty or doubt about the CE provided by the second confederate (which conflicts with the signer’s prior belief) is the most important trigger of eyebrow lowering in such cases. If this is true, then we should expect to see less brow lowering in contrastive conditions if the signer has relatively high confidence in the second confederate, i.e., brow lowering may be overruled by politeness/authority considerations. This is the case in situation 5, where the second confederate (in the role of a ticket seller at the train station) has a comparatively high degree of authority. Indeed, we observe relatively little use of brow lowering in the contrastive conditions here as compared to the other four situations (see Fig. C and Fig. D in S2 File for the plots); however, numbers are small so this issue warrants further study.

There are two additional question forms that are interesting to consider here. Firstly, we may expect questions with a toch tag, when uttered in contrastive conditions, to involve eyebrow lowering to signal doubt about the second confederate’s utterance. As previously discussed in the section *toch tag*, six of the eleven questions with a toch tag occur in one of the two contrastive conditions (the remaining five occurred after non-neutral SB and neutral CE), where the polarity of the SR aligns with SB. As such, this construction signals that the signer believes the information provided by the first confederate is true, thus leading the signer to doubt the CE offered by the second confederate. As expected, all of these instances involve either brow lowering and/or inner brow raising, but never brow raising (for two examples, see (11) earlier). In most of the cases, the brow position is sustained from the SR onto the tag. Secondly, four of the seven ‘I thought...’-type question forms with embedding, discussed in the section *I thought...*, occur in one of the contrastive conditions. Like toch-type questions, these forms foreground the signer’s own expectation with respect to the SR while signaling that it conflicts with the information provided by the interlocutor. Again, all of these instances involve eyebrow lowering; see (16) earlier for two examples.

The three factors just discussed all make a connection between eyebrow lowering and uncertainty/doubt. This can again be linked to previous work on other languages: several studies on nonverbal communicative cues have similarly claimed that brow lowering may signal uncertainty (e.g., [81,82]), while Haviland [83] has described the use of brow lowering (in combination with mouth shrug) as a signal of “doubt, uncertainty, lack of knowledge or understanding, typically directed toward an interlocutor” in Zinacantec Family Homesign (p. 545). Furthermore, in a set of multimodal studies on Catalan and Dutch listeners’ perception of incredulity questions, i.e., questions that convey disbelief about a situation which contradicts the speaker’s own expectations, it is shown that facial expressions play an important role in the interpretation of such questions [84,85]. Importantly, the facial expression associated with incredulity questions is described as “a furrowing of the brows and a squinting of the eyes, often accompanied by a head shake” (p.360). The contrastive NegPos and PosNeg conditions in our experiment may, too, trigger question forms that express a sense of disbelief, i.e., incredulity questions. As we have shown, such forms indeed frequently involve eyebrow lowering.

Table 13 lists all the factors we have argued to be associated with brow lowering, indicating in which situations, which contexts, and for which question forms they play a role.

**Table 13:** Factors promoting the use of brow lowering in polar questions in NGT.

Factor	Situation	CE	Polarity marking	Tag type
*1. Negation*	all	all	negative	*or...*
*2. Negative bouletic bias*	2-5	negative; neutral	negative	–
*3. Uncertainty/doubt about the SR*	all	neutral (especially)	no (especially)	HES; *or...*
*4. Uncertainty/doubt about addressee competence*	all	neutral	all	HES
*5. Uncertainty/doubt about the CE*	1-4	opposite of SB	all	toch

#### Competition between brow raising and lowering.

In the previous subsections, we have proposed that eyebrow raising and lowering are each promoted by a number of factors. Since some of these factors can obtain at the same time (e.g., a positive bouletic bias, promoting brow raising, and uncertainty/doubt about the SR, promoting brow lowering), and since the eyebrows cannot be raised and lowered at the same time, there is bound to be competition between these two configurations in some cases.

Interestingly, overall, brow *lowering* is attested most frequently in our data, even though brow *raising* has generally been regarded as prototypically occurring in polar questions. As such, our data provides evidence that eyebrow raising is **not** actually a marker of polar-questionhood. After all, all of the question forms we analyzed in this study are polar question forms. But if this is the case, then why is eyebrow raising so often cited as *the* non-manual marker of polar questions in sign languages? We believe this can be explained by considering how data has typically been elicited in previous studies on polar questions in sign languages. Often, questions are elicited in out-of-the-blue or otherwise limited contexts (in particular, without explicitly controlling for SB, CE, and bouletic bias). Now, considering the list of factors that we argued to be associated with eyebrow raising in the section Factors associated with brow raising, one of these – namely *introducing a new conversational topic* – is clearly of particular relevance in out-of-the-blue polar questions. Most of the factors we argued to be associated with brow lowering in the section *Factors associated with brow lowering*, on the other hand, do not apply to such forms, perhaps with the exception of uncertainty about the SR and uncertainty about addressee competence in some cases. We suggest, however, that such uncertainty is typically not assumed by default in an out-of-the-blue elicitation context, and thus the likelihood of eyebrow lowering is very low. Only when a conversation further develops, uncertainty can increase (e.g., because of conflicting information). Thus, an out-of-the-blue polar question is likely to involve raised eyebrows – not because eyebrow raising marks the utterance as a polar question, but because there is at least one factor (*introducing a new conversational topic*) strongly promoting the use of eyebrow raising while there are typically no factors strongly promoting the use of eyebrow lowering. Once more contextual factors are introduced, for instance in the form of SB and CE, the likelihood that lowered eyebrows are used increases overall, since such contextual factors may trigger particular expectations or doubts on the part of the signer; some of which could be associated with eyebrow lowering (and some with eyebrow raising). Another exception could in principle be *negation*. However, we have found that polar questions with a negated SR are only used in the presence of negative CE or SB. So they are very unlikely to occur in out-of-the-blue elicitation contexts.

An unanswered question is how, if a competition between brow raising and lowering arises, a ‘winner’ is determined. Indeed, it is striking that brow lowering occurs so frequently across most of our experimental conditions. While we have gone into much depth about the factors we believe can help explain the distributional patterns we observed in the data, we have not really commented on how – within a specific question form – the interaction between these factors leads to a particular outcome, both in terms of the nature (raising versus lowering) and intensity (degree to which the eyebrows are raised/lowered) of the eyebrow configuration. There are different possibilities here. One is that the outcome is determined additively, based on the number of factors involved as well as their relative weight. It is striking, for instance, that in question forms consisting of a SR and a hesitation tag, we *never* observe eyebrow raising in either of the contrastive conditions. This could simply be an additive effect: there are multiple factors associated with brow lowering involved here (e.g., uncertainty/doubt about the SR; uncertainty/doubt about the CE, in some cases negative bouletic bias, in some cases negation), as opposed to perhaps just one or two associated with brow raising (in some cases positive bouletic bias, in some cases surprise). Alternatively, the presence of one or more of these factors (for instance, uncertainty about the SR) could weigh particularly heavily here, such that eyebrow raising is entirely overruled. Another possibility is that sometimes a particular brow configuration is required because it is part of a specific combination of non-manuals connected to a fixed meaning. That is, perhaps there are combinations of non-manuals including the eyebrows that are interpreted non-additively in a Gestalt-like fashion (see [86] for some general discussion on multimodal Gestalt recognition, and [87] for some evidence for the Gestalt-like perception of multimodal communicative signals).

The above discussion also relates to another research question, namely whether affective facial expressions are likely to overrule grammatical facial expressions. In the literature, this is sometimes referred to as the Affect over Grammar hypothesis. Previous experimental studies on eyebrow position in polar questions and other sentence types in different sign languages provide mixed evidence for this hypothesis. For NGT, De Vos et al. [33] conclude that their data do not support the Affect over Grammar Hypothesis. Instead, they argue that eyebrow lowering is generally phonetically stronger than eyebrow raising, independent of linguistic or affective status. This seems to fit with the observation by Nota et al. [88] that eyebrow lowering – but not eyebrow raising – facilitates question identification in question perception in spoken Dutch, both in terms of speed and accuracy. In other words, eyebrow lowering appears to not just be phonetically but also perceptually stronger.

Weast [74], on the other hand, finds some evidence that differences in eyebrow height do not reliably distinguish between polar questions, wh-questions, and declaratives in emotionally marked sentences in ASL, thus providing some support for the Affect over Grammar hypothesis. Finally, Kimmelman et al. [75] find only indirect evidence for the hypothesis for Kazakh-Russian Sign Language, in that they “do not observe that emotional expressions completely override grammatical marking” (p. 13), but they do observe some interactions between sentence type, emotion, and eyebrow position.

Of course, our study was not specifically designed to provide further evidence for or against the Affect over Grammar hypothesis in NGT. However, we do wish to add another consideration to the discussion here. Altogether, our data clearly shows that brow lowering regularly wins out over brow raising. We think that the fact that *uncertainty/doubt* plays a role in many of our experimental conditions may be a major contributor to this general tendency. This introduces another dimension to the Affect over Grammar discussion, as the studies cited above focus specifically on emotional states (anger; surprise) but not on doxastic states such as uncertainty and doubt. In our data, we have seen that uncertainty can be marked by conventionalized linguistic elements in NGT, in the form of hesitation tags and inquisitive headshakes. This raises interesting questions about what this might mean for the predictions that the Affect over Grammar hypothesis would make regarding the use of eyebrow lowering in question forms where uncertainty plays a role. We leave a deeper exploration of this matter to future work.

A final issue we wish to briefly address here concerns inter-participant variation. Since the number of data points per participant is small (max. five question forms per condition, but generally less when we only take SR-only constructions into consideration), it is difficult to draw firm conclusions, but it is nonetheless evident that different participants have different personal preferences for the use of eyebrow lowering or raising (see Fig. E through Fig. J in S2 File for plots). Participant 02, for instance, has a clear preference for eyebrow raise, in particular in conditions with positive and neutral CE, while participants 01 and 06 use very little brow raising, instead showing a preference for brow lowering in most experimental conditions. We see the investigation of inter-participant variation as an important area for further study.

### Function of mouth shrug

We found that a mouth shrug (lipcorners down) very frequently occurred on hesitation tags across all conditions (36 out of 39 cases). In SR-only questions, mouth shrugs are much less common, but still occur in all conditions (proportions range between 14% and 37% across conditions). We propose that a mouth shrug expresses uncertainty in NGT. More specifically, we propose that it can signal uncertainty about the SR, as well as uncertainty about addressee competence (the addressee’s ability to answer the question that is asked). This proposal is supported by two facts. First, in our own data, mouth shrugs very frequently co-occur with hesitation tags, and we have argued that such tags also signal uncertainty (either about the SR or about addressee competence). And second, expressing uncertainty, doubt, or ignorance is one of the functions that has been ascribed to mouth shrug in previous studies on several other sign languages (e.g., [89] on Italian Sign Language; [90] on Danish Sign Language) and (co-speech) gesture (e.g., [82,91]. It is interesting to note here that Debras [91], building on earlier work by [92] and [93], speculates that mouth shrugs may be used in so many spoken languages to express uncertainty or ignorance because they iconically involve a configuration of the mouth which literally makes it impossible to speak, indicating that the speaker ‘has nothing to say’ on the topic at hand. Of course, in sign languages, the mouth is not the only articulator, and not even the most essential one. So for sign languages, the idea that a mouth shrug would iconically convey that the signer has nothing to contribute to the topic at hand does not fly. It *is* possible, in principle, that the mouth shrug first conventionalized in many spoken languages, and then made its way into sign languages through contact. We must leave further investigation of this issue for another occasion.

To return to our proposal, for uses of mouth shrug in SR-only questions, the hypothesis that this marker functions to signal uncertainty is still viable, even if there is no co-occurring hesitation tag, but with an interesting twist. About two-thirds of the instances of mouth shrug in SR-only constructions are compatible with an uncertainty function. In most of these cases, the mouth shrug occurs at the end of the SR, typically accompanying a prosodically light sign such as a pointing sign or a prosodically integrated palm-up. Other instances of mouth shrug, most of which appear at the beginning of the SR, seem to express other types of speaker attitudes, such as disappointment, certainty(!), or surprise. Based on these observations, we suggest that *sentence-final* mouth shrugs express uncertainty (about the truth of the SR or about addressee competence), while mouth shrugs that do not appear sentence-finally fulfill other functions.

In our experimental setting there was always some degree of uncertainty on the part of the signer when asking a polar question. Why, then, did mouth shrug not always occur? In particular, why did it occur much less often in SR-only questions than in questions with a hesitation tag? We suggest that this can be explained by the fact that in SR-only questions the mouth was frequently used to articulate mouthings or mouth actions accompanying lexical signs. Since hesitation tags involve manual signs such as palm-up, which are not lexically associated with a particular mouth action or with mouthing, the mouth and lips were freed up in this case to articulate a mouth shrug.

## Limitations and future work

In this article, we presented a production experiment in which we elicited polar questions in different contexts from six deaf NGT signers. The results show a considerable degree of variation both in terms of the sentence structure of question forms as well as non-manual marking patterns, in particular concerning the position of the eyebrows. We were able to connect some of this variation to differences in the type of SB and CE that was provided in the context in which these questions were asked, as we systematically manipulated these factors in the experiment.

Although we attempted to control for other factors that could potentially have an effect on question form, we did not quite manage to do so in all cases, as pointed out at various places in this article. For instance, we realized only as we were analyzing the data that the five situations in our experiment differed in whether or not they were likely to trigger a particular bouletic bias, which we have shown to have an effect on eyebrow position. We also did not realize that in one of the situations, the second confederate had a higher degree of authority, which sometimes led to the use of politeness markers in question forms. To clarify: what we mean here is that, in one of the situations, the second confederate had a higher degree of authority in the role they enacted in that particular situation. The degree to which participants were familiar with the person who acted as our second confederate may also have influenced the way in which they phrased their questions. Since the Dutch deaf community is quite tight-knit, all participants had some degree of familiarity with both our confederates. Although further information concerning the exact degree of familiarity could indeed be relevant, we did not collect such information and believe it would be difficult to quantify.

While unintentional, failure to fully control for these factors allowed us to uncover more diversity in question forms, and provide clear avenues for further study. We believe that the impact of bouletic bias on question form, both in terms of sentence structure and non-manual marking, forms a particularly interesting area for future study.

There are further factors that future studies on (biased) polar questions in sign languages could take into account. For instance, in our experimental situations, CE was always directly provided by the second confederate. It would be interesting to investigate whether different question forms are used when the source of CE is situational (e.g., seeing it raining outside, or someone entering the room). Indeed, a previous study on biased polar questions in spoken Dutch provides indication that the source of CE is relevant for the acceptability of certain question forms in this language, including Dutch *toch*-questions [51]. It would be interesting to investigate if NGT questions with a toch tag are acceptable in the same situations.

This brings us to another point: given that we did a production study, we were not able to collect any negative evidence. That is, our data do not tell us anything about which question forms are *not* acceptable in a given situation. For instance, we have shown that questions with a toch tag can occur following positive or negative SB, but we did not observe any toch questions after neutral SB. However, we cannot conclude from this that such toch questions are therefore unacceptable in such cases. To do so, we need judgment data from a perception/comprehension study, which we see as a natural area for follow-up research. Such a judgment task may include (some of) the question forms described in the present study and ask native signers of NGT to evaluate their acceptability in different contexts of use.

Another limitation of the present study is that it only involved a small number of signers. Still, even among this group of signers, we observed a high amount of inter-participant variation, both when considering sentence structure as well as non-manual marking patterns. Further research is needed to get a better view on the full extent of variation in this domain. A naturalistic corpus study, e.g., based on the Corpus NGT [31] could be suitable for these purposes, although there is a possibility that such data actually features *less* variation in question forms (and thus more apparent uniformity across participants) depending on whether the data include many non-canonical (as opposed to canonical) polar questions or not. We believe that evidence from a combination of elicited and corpus data is needed to get the best understanding of inter- as well as intra-participant variation in this domain of inquiry.

The complexity of the non-manual marking patterns in our dataset, as scrutinized in the section *Competition between brow raising and lowering*, calls attention to a major empirical question in the study of non-manual behavior in (spoken and signed) language, namely whether non-manual elements have additive or compositional meaning. We distinguish two types of additivity here. First, multiple non-manuals may combine such that each contributes its own meaning component to yield complex (but decomposable) meaning overall. Second, a single non-manual can be used to express multiple meanings simultaneously, which may be reflected in the non-manual being articulated with greater intensity. Compositional, Gestalt-like, meaning arises when a fixed combination of non-manuals gets associated with a meaning which is more than (or different from) the sum of its parts (again, see [86] and [87] for some discussion and evidence for compositional perception of multimodal communicative signals). Evidently, the non-manual markers used in NGT polar questions can be associated with multiple functions; as we discussed at length in the section *Factors associated with raised and lowered eyebrows*, this especially holds for raised and lowered eyebrows. It is certainly also imaginable that there are specific clusters of non-manuals that are attached to a certain meaning in a Gestalt-like fashion. An example could for instance be the combination of eyebrow lowering, (intense) eye squinting, and mouth shrug to express uncertainty. Now, if there is true compositionality here, then one would not expect it to be possible to express uncertainty without using all three of these non-manuals in combination. Whether or not this is possible is an empirical question that could be investigated, e.g., by eliciting polar questions with uncertainty marking in situations where there may also be a need to express, for instance, politeness and/or positive bouletic bias (associated with eyebrow raising). If these latter considerations can override the use of eyebrow lowering, or weaken its intensity, then this weakens the argument for compositionality, rather providing more support for an additive perspective. Such studies would be similar in spirit to the sign language studies we have mentioned where the effect of contrasting affective states and question types on eyebrow position were investigated [33,74,75]. We look forward to more empirical work on this matter in the future.

Finally, throughout this article, we have drawn comparisons wherever possible between our findings and those reported in previous studies on both sign languages and (co-speech) gesture. However, major differences in scope, approach, and methods between studies make a more systematic comparison across languages challenging. Such cross-linguistic, cross-modal comparison may be realized in future work by replicating the production experiment described in this paper for other languages. Indeed, a recent master’s thesis used our experimental design in a study on polar questions involving eleven speakers of Dutch [94]. We shall briefly highlight some notable findings here. Firstly, sentence-structure patterns in the dataset were analyzed similarly to those found in the present study, with subject-verb inversion taken into account as an additional factor. Like the NGT data, the Dutch data include a mixture of question forms with positive, negative, or no polarity marking, where an interesting finding is that the *presence* of polarity marking was shown to correlate with the *absence* of subject-verb inversion, which was otherwise quite common among the question forms in the dataset. Secondly, whereas in our NGT data, approximately one third of all question forms included a tag, this was the case for just 32 out of 252 questions (about 13%) in the Dutch data. We suggest that the frequent use of hesitation tags in the NGT dataset, for which there is no spoken Dutch equivalent, largely explains this difference. Thirdly, different from the present study, Weijland [94] analyzed non-manuals using 3D facial capturing and machine-learning clustering techniques (and see [95] for a similar type of analysis for the NGT data discussed in the present article). Like in our data, variation was attested in the use of brow raise vs. brow lowering across experimental conditions, although the precise distributional patterns differ somewhat from those we reported for NGT. Moreover, both the frequency of occurrence and the intensity of non-neutral brow configurations in the Dutch data are relatively low. Finally, the analysis shows that non-neutral brow positions tend to occur at the beginning of the spoken utterance and decline toward the end, which seems to be different from what we observe in NGT, where brow raising and/or lowering is often sustained for the entire sentence. Thus, Weijland [94] provides some interesting multimodal insight into polar question forms in spoken Dutch. Of course, some of the structural commonalities between Dutch and NGT can probably be explained by the fact that these languages are used by overlapping communities of users, as well as the fact that most NGT users are also (L2) users of Dutch. Adding more, and more diverse, languages to the picture would give us further insight into the typological similarities and differences between canonical and non-canonical polar questions across languages and modalities. We see this as an important avenue for future investigation.

## Conclusion

In this paper, we have provided a rich description and analysis of NGT polar questions, elicited from six NGT signers in a role-play setting in which we systematically manipulated original signer belief and contextual evidence, to yield a variegated set of polar question forms.

With regard to sentence structure, we have shown that the polar questions in our dataset can be categorized into three main types. Some consist of a SR only, others involve a SR followed by one or two ‘tags’, while yet others are complex constructions involving embedding. Moreover, we have shown that (positive or negative) polarity marking often plays a role of importance, where the polarity marking tends to correspond with the polarity of the provided CE. Question forms with tags, too, show characteristic distributional patterns across experimental conditions. Questions with an *or...* tag, for instance, are common after neutral CE, while there were no questions with toch tags in conditions with neutral SB. Hesitation tags, on the other hand, were attested in all experimental conditions. Future work is needed to provide further evidence for the (un)acceptability of particular question forms in specific contexts; the generalizations we formulated in the section Discussion: Polar question structures and their contexts of use may help inform such follow-up work.

We also investigated non-manual marking patterns, on the basis of which we argued that raised eyebrows cannot be the main marker of polar questions in NGT. The most consistently used non-manual marker across all polar question forms was a head/body forward position (‘forward’), which we proposed is actually the main polar question marker in NGT. It is possible that ‘forward’ is even a more general marker of inquisitiveness, although the currently available evidence suggests that it has the narrower function of marking *polar* questions, specifically. Regarding the eyebrows, we connected both raised and lowered eyebrows to a wide range of functions, which we have argued creates a competition between these contrasting brow positions, which in turn accounts for the specific distributional patterns across conditions. An open question remains exactly how a ‘winner’ between eyebrow raising and lowering is decided when there are multiple factors involved. Further research is needed to shed more light on this matter. Finally, we have suggested that mouth shrug serves to indicate uncertainty. That it occurs highly frequently on hesitation tags, but much less so in questions consisting of a SR only, is explained by the fact that the mouth is often engaged otherwise (e.g., to articulate mouthings) in the latter construction type.

To conclude, the findings presented in this paper provide us with significant insight into the polar question forms that exists in NGT and the contexts in which they are used. It is our hope that the experimental design we developed for the present study can be utilized fruitfully in future work on both signed and spoken languages, in order to allow for more direct cross-linguistic (and cross-modal) comparison in this research domain in the future.

## Supporting information

S1 File Situations. This supplementary file includes written representations of the five situations (plus one practice situation) used in the production task.
(PDF)

S2 File Supplementary plots. This supplementary file includes a number of finer-grained plots showing the distribution of eyebrow raising vs. lowering in specific (sets of) situations or for specific participants.
(PDF)

## References

[1] Dryer M. Polar questions. The World Atlas of Language Structures Online. http://wals.info/chapter/116 2013. 2026 February 19.

[2] Polar questions. The Atlas of Pidgin and Creole Language Structures. https://apics-online.info/parameters/103.chapter.html 2013. 2026 February 19.

[3] Ladd RD. A first look at the semantics and pragmatics of negative questions and tag questions. Proceedings of Chicago Linguistic Society. 1981. p. 164–71.

[4] Romero M, Han C-H. On Negative Yes/No Questions. Linguistics and Philosophy. 2004;27(5):609–58. doi: 10.1023/b:ling.0000033850.15705.94

[5] Krifka M. Bias in commitment space semantics: Declarative questions, negated quetions, and question tags. SALT. 2015;25:328-345. doi: 10.3765/salt.v25i0.3078

[6] Farkas DF, Roelofsen F. Division of Labor in the Interpretation of Declaratives and Interrogatives. J Semantics. 2017;34(2):237-289. doi: 10.1093/jos/ffw012

[7] Goodhue D. Isn’t there more than one way to bias a polar question?. Natural Language Semantics. 2022;30(4):379–413. doi: 10.1007/s11050-022-09198-2

[8] Romero M. Biased Polar Questions. Annu Rev Linguist. 2024;10(1):279–302. doi: 10.1146/annurev-linguistics-022421-064837

[9] Gilchrist S. Standardisation and lexical preferences in the deaf community of the Netherlands [MA thesis]. University of Amsterdam. 2024. https://scripties.uba.uva.nl/search?id=c11101761

[10] Klomp U. A descriptive grammar of Sign Language of the Netherlands [PhD thesis]. University of Amsterdam. 2021. https://hdl.handle.net/11245.1/d2951072-74d6-417f-8891-3759e35951af

[11] Parks E, Williams H. Sociolinguistic profiles of twenty-four deaf communities in the Americas. 2011–036. 2011. https://www.sil.org/resources/publications/entry/41653

[12] Coerts J. Nonmanual grammatical markers: An analysis of interrogatives, negations and topicalisations in Sign Language of the Netherlands [PhD thesis]. University of Amsterdam. 1992.

[13] Schermer T, Koolhof C. Basiswoordenboek Nederlandse Gebarentaal. Utrecht: Van Dale. 2009.

[14] Zeshan U. Interrogative Constructions in Signed Languages: Crosslinguistic Perspectives. Language. 2004;80(1):7–39. doi: 10.1353/lan.2004.0050

[15] Zeshan U. Interrogative and negative constructions in sign language. Nijmegen: Ishara Press. 2006. doi: 10.26530/oapen_453832

[16] Büring D, Gunlogson C. Aren’t positive and negative polar questions the same? [Unpublished manuscript]. 2000. http://hdl.handle.net/1802/1432

[17] AnderBois S. Issues and alternatives [PhD thesis]. University of California, Santa Cruz. 2011.

[18] Roelofsen F, Venhuizen N, Sassoon GW. Positive and negative questions in discourse. In: Proceedings of Sinn und Bedeutung, 2013. 455–72.

[19] Domaneschi F, Romero M, Braun B. Bias in polar questions: Evidence from English and German production experiments. Glossa: a journal of general linguistics. 2017;2(1). doi: 10.5334/gjgl.27

[20] van Rooij R, Safárová M. On polar questions. UMass Occasional Papers in Linguistics. Ithaca, NY: CLC Publications. 2003. p. 292–309. doi: 10.3765/salt.v13i0.2887

[21] Tabatowski M. Preferring to learn: an attitudinal approach to polar questions [PhD thesis]. The University of Chicago. 2022.

[22] Moody B. La langue des signes. Vincennes, France: International Visual Theatre - Centre Socio-Culturel des Sourds. 1983.

[23] Parini A. Indexical signs (IX) in clause-final position in Italian Sign Language (LIS): A preliminary study [MA thesis]. Università Ca’Foscari Venezia. 2022. https://hdl.handle.net/20.500.14247/14533

[24] Yang JH, Fischer S. Expressing negation in Chinese Sign Language. Sign Language & Linguistics. 2002;5(2):167–202. doi: 10.1075/sll.5.2.05yan

[25] Arnold B. Discourse functions of palm-up in British Sign Language (MSc thesis]. University College London. 2019.

[26] Gökgöz K, Wilbur RB. Positive bias in negative yes/no questions: Evidence for Neg-to-C in TİD. Different faces of sign language research. Warsaw: Faculty of Polish Studies, University of Warsaw. 2017. p. 15–41.

[27] Bergman B. Non-manual components of signed language: Some sentence types in Swedish Sign Language. Recent research on European sign languages. Lisse: Swets & Zeitlinger. 1984. p. 49–59.

[28] Cañas Peña S. The marking of polar interrogatives in Catalan Sign Language. Proceedings of the Conference of the Student Organization of Linguistics in Europe. Leiden University Centre for Linguistics. 2019. p. 1–16.

[29] Sze F, Lee H. Negative polar questions in Hong Kong Sign Language. East Asian Sign Linguistics. De Gruyter. 2022. p. 203–40. doi: 10.1515/9781501510243-008

[30] Gonzalez A, Henninger K, Davidson K. Answering negative questions in American Sign Language. In: Proceedings of North East Linguistic Society (NELS). GLSA, University of Massachusetts at Amherst; 2019. p. 31–44.

[31] Crasborn O, Zwitserlood I, Ros J. Collection “Corpus NGT”. The Language Archive. http://hdl.handle.net/hdl:1839/00-0000-0000-0004-DF8E-6. 2006. 2026 February 19.

[32] van Loon E. What’s in the palm of your hand? Discourse functions of the palm-up in Sign Language of the Netherlands [MA thesis]. University of Amsterdam. 2012.

[33] de Vos C, van der Kooij E, Crasborn O. Mixed signals: combining linguistic and affective functions of eyebrows in questions in sign language of the Netherlands. Lang Speech. 2009;52(2-3):315–39. doi: 10.1177/0023830909103177 19624034

[34] Ekman P, Friesen WV. Facial Action Coding System (FACS). doi: 10.1037/t27734-000 1978.

[35] Schermer T, Harder R. Lexical variation in Dutch Sign Language: Some implications for language planning. Signs of life: Proceedings of the Second European congress of sign language research. Amsterdam: NSDSK. 1986. p. 134–41.

[36] Schermer GM. From Variant to Standard: An Overview of the Standardization Process of the Lexicon of Sign Language of the Netherlands over Two Decades. Sign Language Studies. 2003;3(4):469–86. doi: 10.1353/sls.2003.0017

[37] Oomen M, Roelofsen F. Biased polar questions in Sign Language of the Netherlands - Methods description. University of Amsterdam / Amsterdam University of Applied Sciences. 2022.

[38] Cooperrider K, Abner N, Goldin-Meadow S. The palm-up puzzle: Meanings and origins of a widespread form in gesture and sign. Frontiers in Communication. 2018;3. doi: 10.3389/fcomm.2018.00023

[39] van Boven C. The marking of imperatives in Sign Language of the Netherlands [MA thesis]. University of Amsterdam. 2019.

[40] Oomen M, Pfau R. Signing not (or not): A typological perspective on standard negation in Sign Language of the Netherlands. Linguistic Typology. 2017;21(1):1–51. doi: 10.1515/lingty-2017-0001

[41] Hogeweg L. The meaning and interpretation of the Dutch particle wel. Journal of Pragmatics. 2009;41(3):519–39. doi: 10.1016/j.pragma.2008.06.012

[42] Oomen M, Esselink L, de Ronde T, Roelofsen F. First steps towards a procedure for annotating non-manual markers in sign languages. Proceedings of North East Linguistic Society (NELS); 2023. p. 257-66.

[43] McKee RL, Wallingford S. So, well, whatever: Discourse functions of palm-up in New Zealand Sign Language. Sign Language & Linguistics. 2011;14:213–47. doi: 10.1075/sll.14.2.01mck

[44] Arnold B, Ferrara L. “Your Turn!”: Using Finger Pointing and palm-up Actions to Ask Questions in Norwegian Sign Language. Sign Language Studies. 2024;24(3):621–51. doi: 10.1353/sls.2024.a928058

[45] Oomen M, Roelofsen F. Biased polar question forms in Sign Language of the Netherlands (NGT). FEAST. 2023;5:156–68. doi: 10.31009/feast.i5.13

[46] Kendon A. Some uses of the head shake. Gesture. 2002;2(2):147–82. doi: 10.1075/gest.2.2.03ken

[47] McClave EZ. Linguistic functions of head movements in the context of speech. Journal of Pragmatics. 2000;32(7):855–78. doi: 10.1016/s0378-2166(99)00079-x

[48] Johnston T. A corpus-based study of the role of headshaking in negation in Auslan (Australian Sign Language): Implications for signed language typology. Linguistic Typology. 2018;22(2):185–231. doi: 10.1515/lingty-2018-0008

[49] Pfau R. The grammaticalization of headshakes: From head movement to negative head. New Directions in Grammaticalization Research. Amsterdam: John Benjamins. 2015. p. 9–50. doi: 10.1075/slcs.166.02pfa

[50] Englert C. Questions and responses in Dutch conversations. Journal of Pragmatics. 2010;42(10):2666–84. doi: 10.1016/j.pragma.2010.04.005

[51] Gaasbeek AM. Polar question types in Sign Language of the Netherlands and Dutch [MSc thesis]. University of Amsterdam. 2023. https://eprints.illc.uva.nl/id/eprint/2249

[52] Buysse L. Counterexpectational Translations: The Dutch Markers Toch and Eigenlijk Contrasted with Their English Correspondents. Contrastive Pragmatics. 2022;4(2):178–212. doi: 10.1163/26660393-bja10050

[53] Roelofsen F, Farkas DF. Polarity particle responses as a window onto the interpretation of questions and assertions. Language. 2015;91(2):359–414. doi: 10.1353/lan.2015.0017

[54] Biezma M. Alternative vs Polar Questions: the cornering effect. SALT. 2015;19:37-54. doi: 10.3765/salt.v0i0.2519

[55] Hodge G, Manrique E, Winter B, Cormier K. Manual wh-signs and English wh-mouthings differentiate BSL content and polar questions. Linguistics. 2025;63(5):1339–81. doi: 10.1515/ling-2023-0238

[56] Göksel A, Kelepir M. The phonological and semantic bifurcation of the functions of an articulator: HEAD in questions in Turkish Sign Language. Sign Language & Linguistics. 2013;16:1–30. doi: 10.1075/sll.16.1.01gok

[57] Legeland I. Syntactic and prosodic properties of wh-questions in Sign Language of the Netherlands: A corpus-based investigation [MA thesis]. University of Amsterdam. 2018.

[58] Li X. Leaning and recipient intervening questions in Mandarin conversation. Journal of Pragmatics. 2014;67:34–60. doi: 10.1016/j.pragma.2014.03.011

[59] Rasmussen G. Inclined to better understanding—The coordination of talk and ‘leaning forward’ in doing repair. Journal of Pragmatics. 2014;65:30–45. doi: 10.1016/j.pragma.2013.10.001

[60] Kendrick KH. The intersection of turn-taking and repair: the timing of other-initiations of repair in conversation. Front Psychol. 2015;6:250. doi: 10.3389/fpsyg.2015.00250 25814968 PMC4357221

[61] Trujillo JP, Holler J. The Kinematics of Social Action: Visual Signals Provide Cues for What Interlocutors Do in Conversation. Brain Sci. 2021;11(8):996. doi: 10.3390/brainsci11080996 34439615 PMC8393665

[62] Cecchetto C. Sentence types. Sign Language: An International Handbook. De Gruyter Mouton. 2012. p. 292–315. doi: 10.1515/9783110261325.292

[63] Ekman P. About brows: Emotional and conversational signals. Human Ethology. Cambridge: Cambridge University Press. 1979. p. 163–202.

[64] Khatin-Zadeh O, Farsani D, Hu J, Farina M, Banaruee H, Marmolejo-Ramos F. Distributed embodiment of metaphorical hope in hand, head, and eyebrow gestures. Front Psychol. 2023;14:1139881. doi: 10.3389/fpsyg.2023.1139881 37034906 PMC10075202

[65] de Souza MR. Multimodal responses to questions: turn-initial oh coordinating with raised eyebrows in video-mediated interaction. Gesprächsforschung - Online-Zeitschrift zur verbalen Interaktion. 2024;25:54–74.

[66] Manrique E. Other-initiated Repair in Argentine Sign Language. Open Linguistics. 2016;2(1). doi: 10.1515/opli-2016-0001

[67] Hömke P, Levinson SC, Emmendorfer AK, Holler J. Eyebrow movements as signals of communicative problems in human face-to-face interaction. R Soc Open Sci. 2025;12(3):241632. doi: 10.1098/rsos.241632 40078914 PMC11896710

[68] Chovil N. Discourse‐oriented facial displays in conversation. Research on Language & Social Interaction. 1991;25(1–4):163–94. doi: 10.1080/08351819109389361

[69] Cassell J, Nakano YI, Bickmore TW, Sidner CL, Rich C. Non-verbal cues for discourse structure. In: Proceedings of the 39th Annual Meeting on Association for Computational Linguistics - ACL ’01, 2001. 114–23. 10.3115/1073012.1073028

[70] Flecha-García ML. Eyebrow raises in dialogue and their relation to discourse structure, utterance function and pitch accents in English. Speech Communication. 2010;52(6):542–54. doi: 10.1016/j.specom.2009.12.003

[71] Kimmelman V. Topics and topic prominence in two sign languages. Journal of Pragmatics. 2015;87:156–70. doi: 10.1016/j.pragma.2015.08.004

[72] Mapson R. Polite appearances: How non-manual features convey politeness in British Sign Language. Journal of Politeness Research. 2014;10(2):157–84. doi: 10.1515/pr-2014-0008

[73] Trees AR, Manusov V. Managing Face Concerns in Criticism Integrating Nonverbal Behaviors as a Dimension of Politeness in Female Friendship Dyads. Human Comm Res. 1998;24(4):564–83. doi: 10.1111/j.1468-2958.1998.tb00431.x

[74] Weast T. American Sign Language tone and intonation: A phonetic analysis of eyebrow properties. Formational Units in Sign Languages. Berlin, Boston: De Gruyter. 2011. p. 203–26. doi: 10.1515/9781614510680.203

[75] Kimmelman V, Imashev A, Mukushev M, Sandygulova A. Eyebrow position in grammatical and emotional expressions in Kazakh-Russian Sign Language: A quantitative study. PLoS One. 2020;15(6):e0233731. doi: 10.1371/journal.pone.0233731 32484837 PMC7266324

[76] Zeshan U. Hand, head, and face: Negative constructions in sign languages. Linguistic Typology. 2004;8:1–58. doi: 10.1515/lity.2004.003

[77] Benitez-Quiroz CF, Wilbur RB, Martinez AM. The not face: A grammaticalization of facial expressions of emotion. Cognition. 2016;150:77–84. doi: 10.1016/j.cognition.2016.02.004 26872248 PMC4832078

[78] Gökgöz K. Topics in Turkish Sign Language (Türk İşaret Dili – TİD) Syntax: Verb movement, negation and clausal architecture [MA Thesis]. Boğaziçi University; 2009.

[79] Dikyuva H, Makaroğlu B, Ar ık E. Turkish Sign Language Grammar. Ankara: Ministry of Family and Social Policies Press. 2017.

[80] Farkas DF. Non-Intrusive Questions as a Special Type of Non-Canonical Questions. Journal of Semantics. 2022;39(2):295–337. doi: 10.1093/jos/ffac001

[81] Givens DB, White J. The nonverbal dictionary of gestures, signs & body language cues. Spokane, WA: Center for Nonverbal Studies Press. 2002.

[82] Ricci Bitti PE, Bonfiglioli L, Melani P, Caterina R, Garotti P. Expression and communication of doubt/uncertainty through facial expression. Journal of Theories and Research in Education. 2014;9:159–77. doi: 10.6092/issn.1970-2221/4296

[83] Haviland JB. Grammaticalizing the face in a first generation sign language: The case of “Z”. Historical linguistics 2015: Selected papers from the 22nd International Conference on Historical Linguistics. Amsterdam: John Benjamins. 2019. doi: 10.1075/cilt.348.25hav

[84] Borràs-Comes J, Prieto P. ‘Seeing tunes.’ The role of visual gestures in tune interpretation. Laboratory Phonology. 2011;2(2). doi: 10.1515/labphon.2011.013

[85] Crespo Sendra V, Kaland C, Swerts M, Prieto P. Perceiving incredulity: The role of intonation and facial gestures. Journal of Pragmatics. 2013;47(1):1–13. doi: 10.1016/j.pragma.2012.08.008

[86] Holler J, Levinson SC. Multimodal Language Processing in Human Communication. Trends in Cognitive Sciences. 2019;23:639–52. doi: 10.1016/j.tics.2019.05.006 31235320

[87] Trujillo JP, Holler J. Conversational facial signals combine into compositional meanings that change the interpretation of speaker intentions. Sci Rep. 2024;14(1):2286. doi: 10.1038/s41598-024-52589-0 38280963 PMC10821935

[88] Nota N, Trujillo JP, Holler J. Conversational Eyebrow Frowns Facilitate Question Identification: An Online Study Using Virtual Avatars. Cogn Sci. 2023;47(12):e13392. doi: 10.1111/cogs.13392 38058215

[89] Gianfreda G, Volterra V, Zuczkowski A. L’espressione dell’incertezza nella Lingua dei Segni Italiana (LIS). Journal of Theories and Research in Education. 2014;9:199–234.

[90] Engberg-Pedersen E. The mouth shrug and facial consent in Danish Sign Language. Signed Language and Gesture Research in Cognitive Linguistics. De Gruyter. 2023. p. 329–56. doi: 10.1515/9783110703788-013

[91] Debras C. The shrug: Forms and meanings of a compound enactment. Gesture. 2017;16:1–34. doi: 10.1075/gest.16.1.01deb

[92] Streeck J. Gesturecraft: The manufacture of meaning. Amsterdam: John Benjamins. 2009. doi: 10.1075/gs.2

[93] Müller C. Forms and uses of the palm up open hand: A case of a gesture family. The semantics and pragmatics of everyday gestures. Berlin: Weidler. 2004. p. 233–56.

[94] Weijland EL. An analysis of visual and morphosyntactic cues in biased polar questions in Dutch [MSc thesis]. University of Amsterdam. 2024. https://eprints.illc.uva.nl/id/eprint/2303/

[95] Esselink L, Oomen M, Roelofsen F. Exploring new methods for measuring, analyzing, and visualizing facial expressions. FEAST. 2023;5:35–48. doi: 10.31009/feast.i5.04

