# Supplementary material for: Polar questions in Dutch Sign Language (NGT): A production experiment
Source: PLoS One. 2026 Jul 29;21(7):e0354015. doi: 10.1371/journal.pone.0354015 (PMC13421764; doi:10.1371/journal.pone.0354015)
Supplement: S1 File — This supplementary file includes written representations of the five situations (plus one practice situation) used in the production task. (PDF) [file pone.0354015.s001.pdf]

Supplementary File 1 to
Polar questions in Dutch Sign Language (NGT): A production experiment

Marloes Oomen, Lyke Esselink and Floris Roelofsen
University of Amsterdam

Situations

For the production experiment, we created five situations and one practice situation. Video recordings of the contexts (two per situation) in NGT, as they were also shown to the participants, are available at https://doi.org/10.21942/uva.21695150. Confederate responses provide positive ('+'), neutral ('0'), or negative ('–') evidence for the target question (final participant utterance). The picture prompt images are reprinted from [1] under a CC BY license, with permission from University of Amsterdam / Amsterdam University of Applied Sciences, original copyright 2022.

Practice situation: Is there a metro station nearby?

1. ***Original speaker bias***

Context 1: You recently moved to the center of Amsterdam. You would like to take the metro to Artis [zoo in Amsterdam]. You don't know if there's a metro station nearby. You meet Ria, who lives close to Artis. Ask her.

Participant: "Is there a metro station nearby Artis?"

Confederate A:
+ "Yes, there is a metro station close to Artis."
0 "I don't know, I never take the metro."
– "No, there's no metro station near Artis."

2. ***Contextual evidence***

Context 2: You're meeting your new neighbor Tom for the first time. Ask him whether he knows the way to Artis.

Participant: "Do you know the way to Artis?"

Confederate B:
+ "There's a metro station here around the corner. You should take line 51 to Weesperplein, which is close to Artis."
0 "It's best to go by public transport."
– "You can't take the metro, because there's no metro station near Artis. You should take tram 17."

3. ***Target question***

Picture prompt: 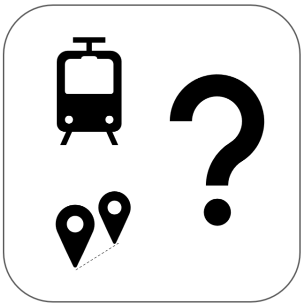

Participant: Variation on "Is there a metro station nearby?"

Situation 1: Is Kim a vegetarian?

1. *Original speaker bias*

Context 1: You're organizing a dinner. You've also invited Kim, but you don't know if Kim is a vegetarian. Ria knows Kim well. Ask her.

Participant: "Is Kim a vegetarian?"

Confederate A:
+ "Yes, Kim is a vegetarian."
0 "I don't know if Kim is a vegetarian."
– "No, Kim is not a vegetarian."

2. *Contextual evidence*

Context 2: You and Tom are cooking dinner together. You're making meatballs. Ask Tom how many meatballs you should make.

Participant: "How many meatballs should we make?"

Confederate B:
+ "You don't have to make any for Kim, she is a vegetarian"
0 "Let's make two for everyone, except for the vegetarians."
– "We should definitely make enough for Kim, she loves them!"

3. *Target question*

Picture prompt:

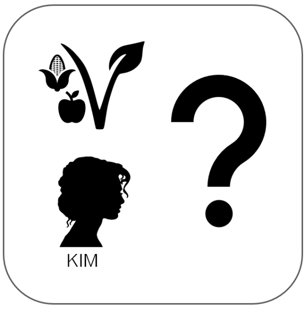

(Version 1)

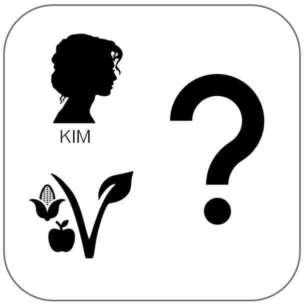

(Version 2)

Participant: Variation on "Is Kim a vegetarian?"

Situation 2: Is the park open?

1. *Original speaker bias*

Context 1: You want to go to the Efteling [Dutch theme park] this weekend, but youre not sure its open. You meet Ria, who has a subscription to the park. Ask her.

Participant: “Is the Efteling open this weekend?”

Confederate A:

+ “Yes, the Efteling is open this weekend.”
0 “Its open on Saturday but I dont know about Sunday. I never go on Sunday.”
– “Its open on Saturday but I think I read in the newspaper that its not open on Sunday.”

2. *Contextual evidence*

Context 2: Later that day, you meet Tom. He works at the Efteling. You know he has the weekend off. Ask him if hed like to come to the Efteling with you this weekend.

Participant: “Do you want to go to the Efteling with me?”

Confederate B:

+ “Fun! Shall we go on Sunday?”
0 “I can’t this weekend.”
– “The Efteling is only open on Saturday. Im available then.”

3. *Target question*

Picture prompt:

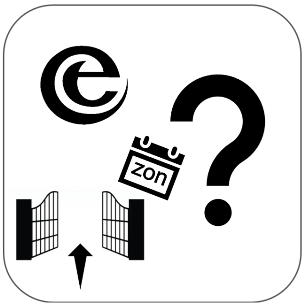

(Version 1)

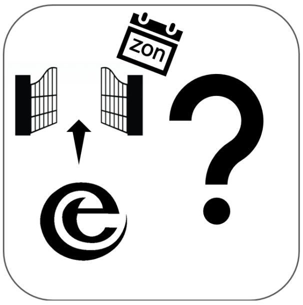

(Version 2)

Participant: Variation on “Is the Efteling open this weekend?”

Situation 3: Is entrance free of charge?

1. ***Original speaker bias***

Context 1:		You would like to visit the Veluwe [Dutch national park] tomorrow. You don't know if entrance is free of charge. Ria is a volunteer at the park. Ask her.
Participant:		"Is entrance to the Veluwe free of charge?"
Confederate A:	+	"Yes, you don't have to pay a fee."
	0	"I don't know."
	–	"No, a ticket costs 10 euros."

2. ***Contextual evidence***

Context 2:		A day later, you're at the Veluwe parking lot. You can't find the entrance to the park. At the parking lot, you meet Tom, another visitor to the park. Ask him.
Participant:		"Do you know where the entrance is?"
Confederate B:	+	"The entrance is there by the white flag. You don't need a ticket."
	0	"The entrance is there by the white flag."
	–	"The entrance is there by the white flag, but you need to get a ticket at the ticket counter over there first."

3. ***Target question***

Picture prompt:

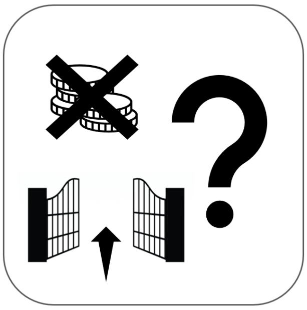

(Version 1)

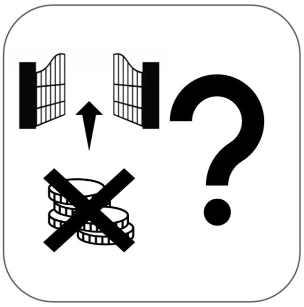

(Version 2)

Participant: Variation on "Is entrance free of charge?"

Situation 4: Is Kim home?

1. ***Original speaker bias***

Context 1: You're a student and you're living together with Ria, Tom, and Kim. You're planning to visit your parents this weekend. You know that Ria and Tom will also be away. You don't know if Kim will stay at home. Ask Ria.

Participant: "Will Kim stay at home?"

Confederate A:
+ "Yes, she needs to study all weekend."
0 "I don't know if she'll stay at home."
– "I thought Kim said she going to spend a weekend at sea."

2. ***Contextual evidence***

Context 2: On Saturday morning, you unexpectedly have to return home early, but you forgot your keys. On the way home, you call Tom; you can't get a hold of Kim. Ask Tom if Kim could open the door for you.

Participant: "Can Kim open the door for me?"

Confederate B:
+ "Yes, I just talked to her and she's there."
0 "I don't know. You should send her a text."
– "Kim is away for the weekend."

3. ***Target question***

Picture prompt:

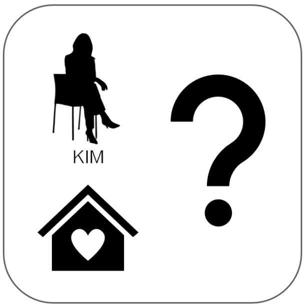

(Version 1)

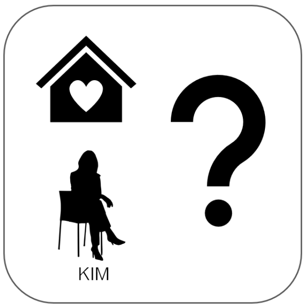

(Version 2)

Participant: Variation on "Is Kim home?"

Situation 5: Is there a train at 9am?

1. *Original speaker bias*

Context 1: Tomorrow morning, you'd like to take the train from Amsterdam to Paris. You'd prefer to leave at 9am. But you don't know if there's a train at 9. Ria has a public transportation travel planner app on her phone. Ask her.

Participant: "Is there a train from Amsterdam to Paris at 9am tomorrow?"

Confederate A:
+ "Let me check. Yes, there's a train at 9am"
0 "Oh, the app doesn't work, so I don't know."
– "Let me check the app. No, I don't see a train at 9am."

2. *Contextual evidence*

Context 2: You live close to the train station, so you decide to walk to the ticket counter to buy a ticket. Ask the ticket seller how much a ticket costs for the train to Paris tomorrow.

Participant: "How much does a ticket for the train to Paris tomorrow cost?"

Confederate B:
+ "For the 9 o'clock train, a ticket costs 100 euros."
0 "It depends on what time you'd like to leave. There are multiple trains going tomorrow."
– "There's only one train tomorrow, which leaves at 10am. A ticket costs 100 euros."

3. *Target question*

Picture prompt:

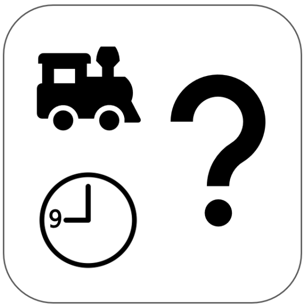

(Version 1)

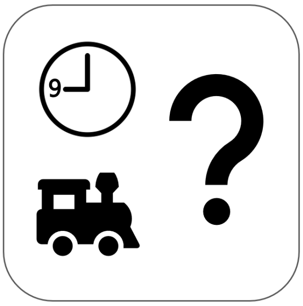

(Version 2)

Participant: Variation on "Is there a train at 9am?"

References

1. Oomen M, Roelofsen F. Biased polar questions in Sign Language of the Netherlands - Methods description. University of Amsterdam / Amsterdam University of Applied Sciences; 2022. doi:10.21942/uva.21701954.v2.
