# Supplementary material for: Polar questions in Dutch Sign Language (NGT): A production experiment
Source: PLoS One. 2026 Jul 29;21(7):e0354015. doi: 10.1371/journal.pone.0354015 (PMC13421764; doi:10.1371/journal.pone.0354015)
Supplement: S2 File — This supplementary file includes a number of finer-grained plots showing the distribution of eyebrow raising vs. lowering in specific (sets of) situations or for specific participants. (PDF) [file pone.0354015.s002.pdf]

Supplementary File 2 to
Polar questions in Dutch Sign Language (NGT): A production experiment

Marloes Oomen, Lyke Esselink and Floris Roelofsen
University of Amsterdam

Supplementary plots

In this supplementary file, we present a number of finer-grained plots showing the distribution of brow raising vs. lowering in specific (sets of) situations or for specific participants.

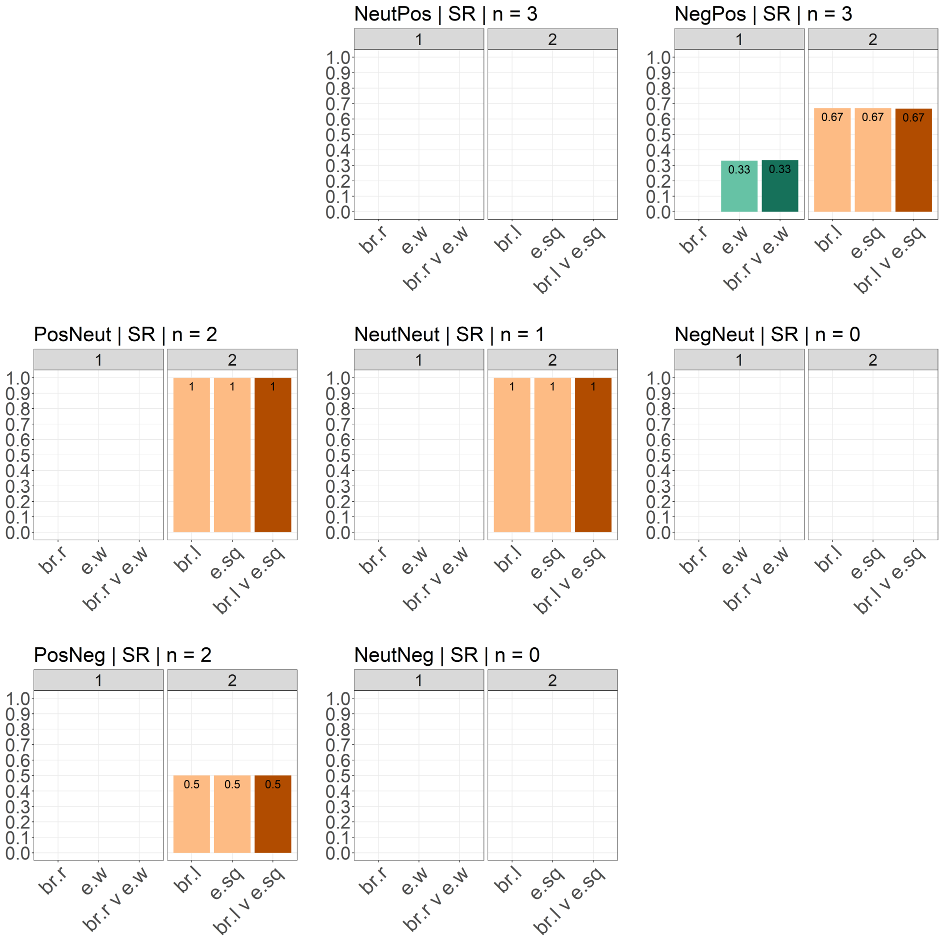

Figure A: Proportions of brow position and eye shape non-manuals per experimental condition in question forms consisting of only a SR, with positive or no polarity marking, uttered in the context of **situation 1** (see Supplementary File 1). Brow raising and eyes wide are presented in green on the left, and brow lowering and eye squint are presented in orange on the right of each condition subplot. The third bar in each subplot indicates the proportion of question forms with either brow raising/lowering or eye widening/squinting, or both. The number of tokens per condition based on which the proportions are calculated are reported in the upper right corner of each bar chart.

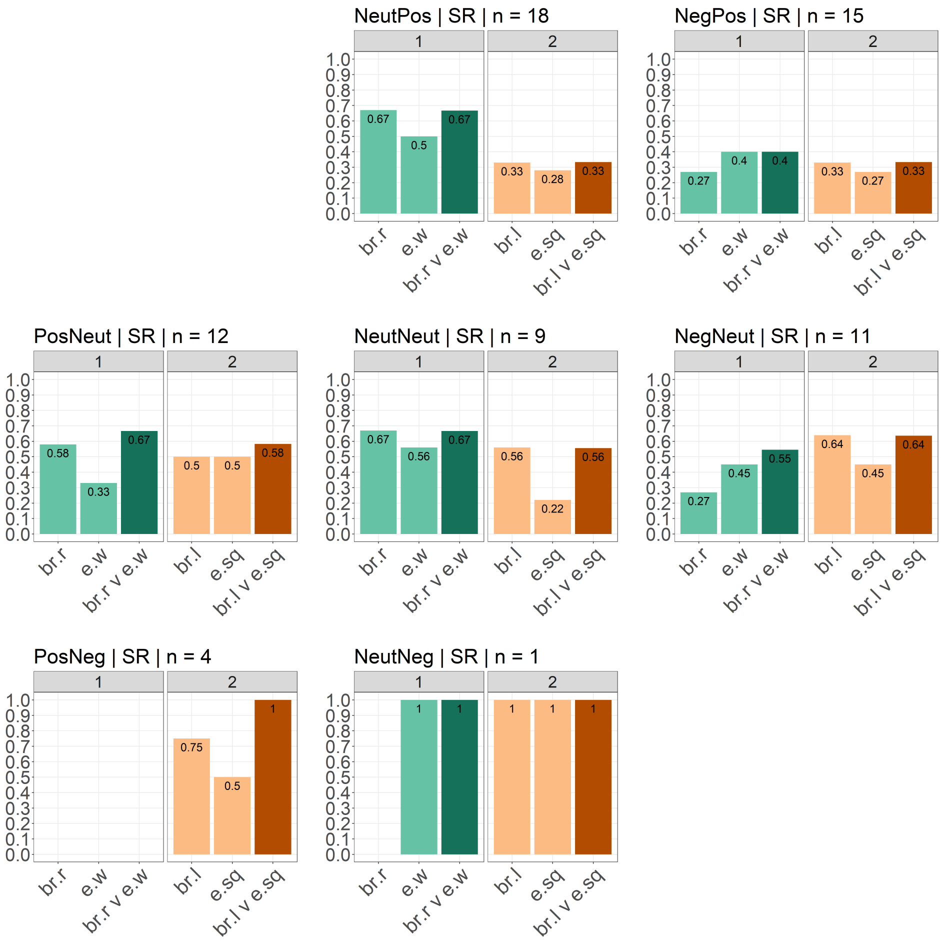

Figure B: Proportions of brow position and eye shape non-manuals per experimental condition in question forms consisting of only a SR, with positive or no polarity marking, uttered in the context of **situations 2-5** (see Supplementary File 1). Brow raising and eyes wide are presented in green on the left, and brow lowering and eye squint are presented in orange on the right of each condition subplot. The third bar in each subplot indicates the proportion of question forms with either brow raising/lowering or eye widening/squinting, or both. The number of tokens per condition based on which the proportions are calculated are reported in the upper right corner of each bar chart.

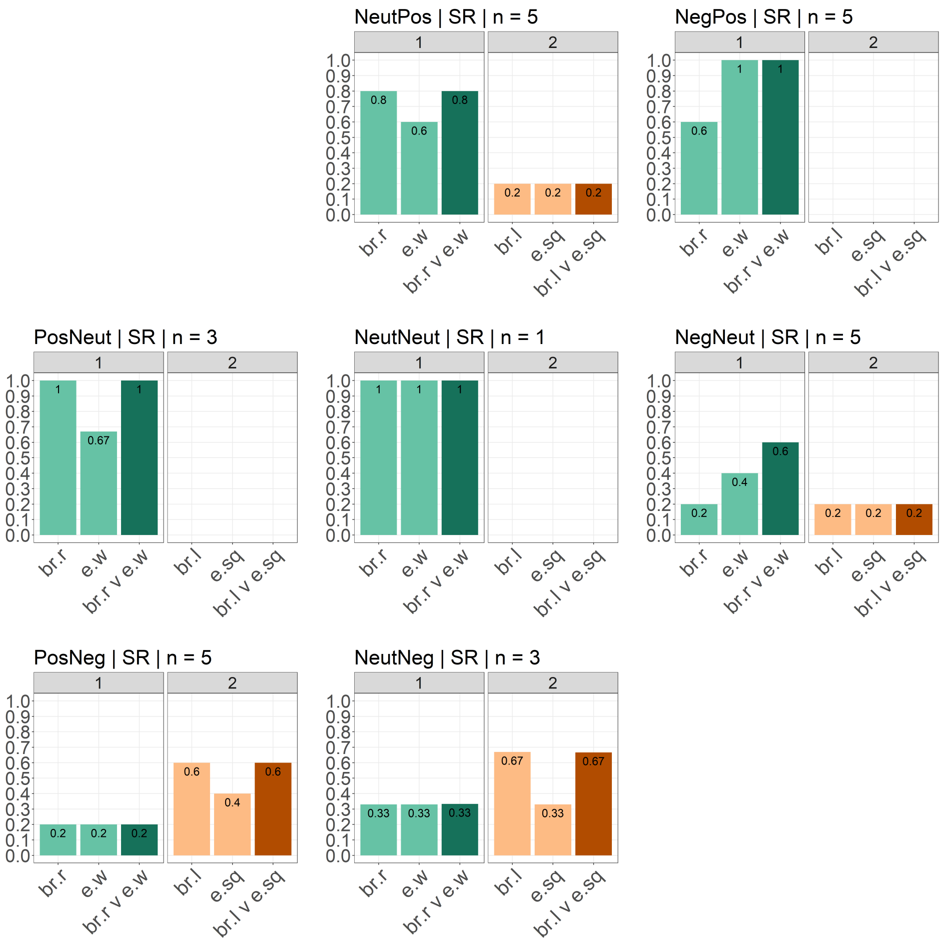

Figure C: Proportions of brow position and eye shape non-manuals per experimental condition in question forms consisting of only a SR, uttered in the context of **situation 5** (see Supplementary File 1). Brow raising and eyes wide are presented in green on the left, and brow lowering and eye squint are presented in orange on the right of each condition subplot. The third bar in each subplot indicates the proportion of question forms with either brow raising/lowering or eye widening/squinting, or both. The number of tokens per condition based on which the proportions are calculated are reported in the upper right corner of each bar chart.

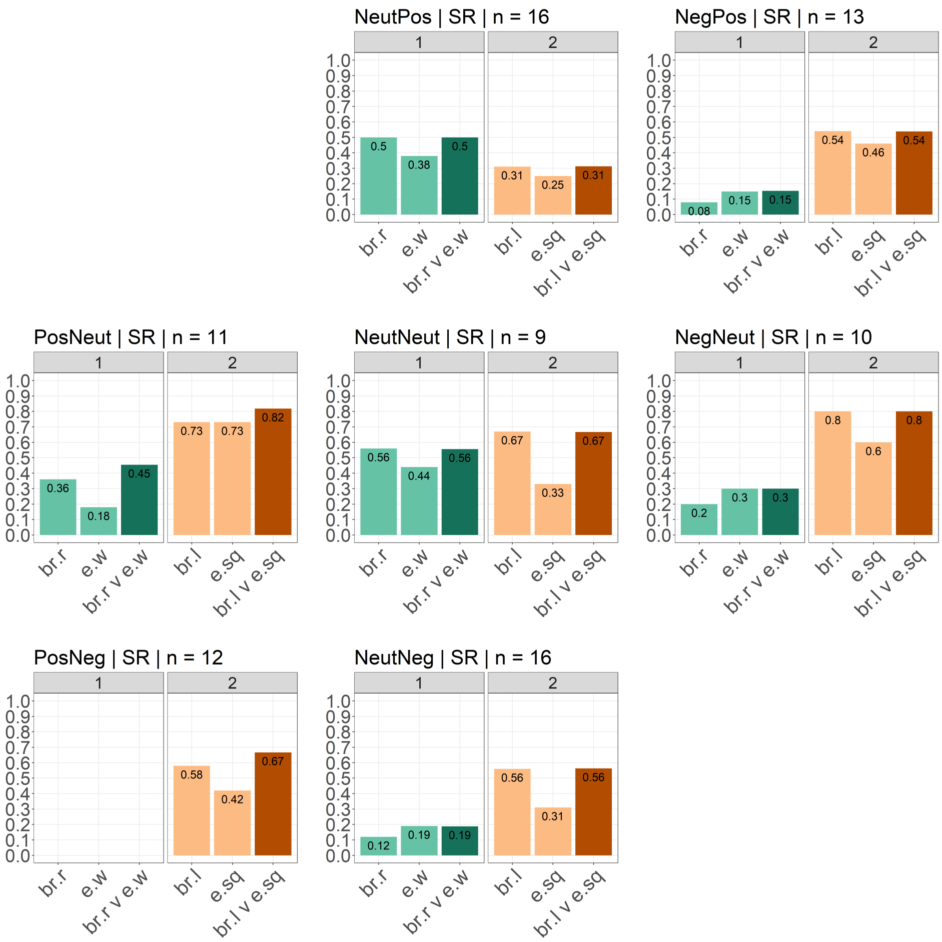

Figure D: Proportions of brow position and eye shape non-manuals per experimental condition in question forms consisting of only a SR, uttered in the context of **situations 1-4** (see Supplementary File 1). Brow raising and eyes wide are presented in green on the left, and brow lowering and eye squint are presented in orange on the right of each condition subplot. The third bar in each subplot indicates the proportion of question forms with either brow raising/lowering or eye widening/squinting, or both. The number of tokens per condition based on which the proportions are calculated are reported in the upper right corner of each bar chart.

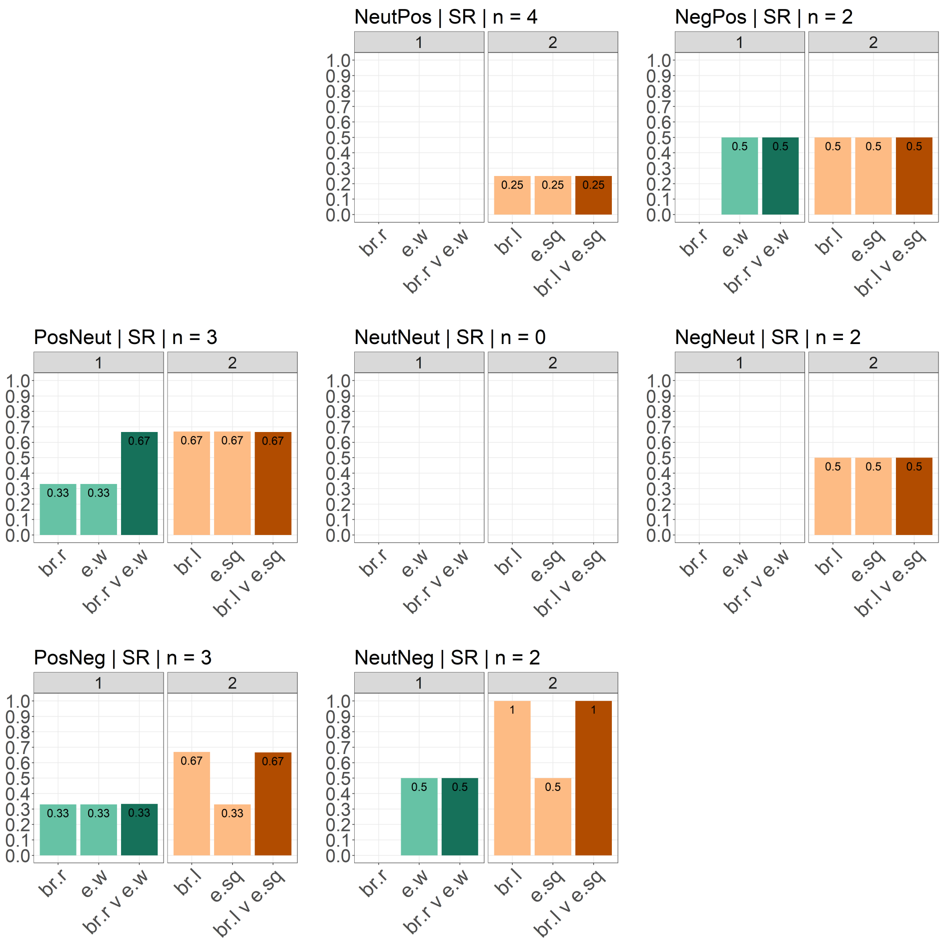

Figure E: Proportions of brow position and eye shape non-manuals per experimental condition in question forms consisting of only a SR, uttered by **participant 01**. Brow raising and eyes wide are presented in green on the left, and brow lowering and eye squint are presented in orange on the right of each condition subplot. The third bar in each subplot indicates the proportion of question forms with either brow raising/lowering or eye widening/squinting, or both. The number of tokens per condition based on which the proportions are calculated are reported in the upper right corner of each bar chart.

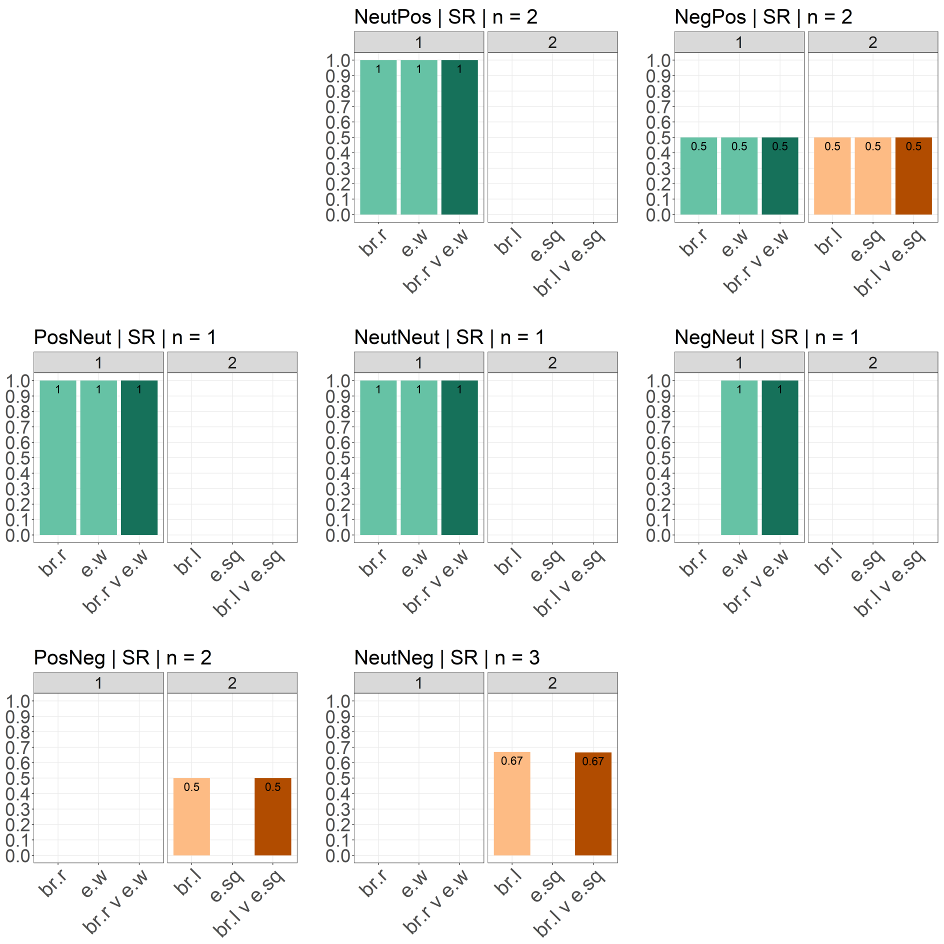

Figure F: Proportions of brow position and eye shape non-manuals per experimental condition in question forms consisting of only a SR, uttered by **participant 02**. Brow raising and eyes wide are presented in green on the left, and brow lowering and eye squint are presented in orange on the right of each condition subplot. The third bar in each subplot indicates the proportion of question forms with either brow raising/lowering or eye widening/squinting, or both. The number of tokens per condition based on which the proportions are calculated are reported in the upper right corner of each bar chart.

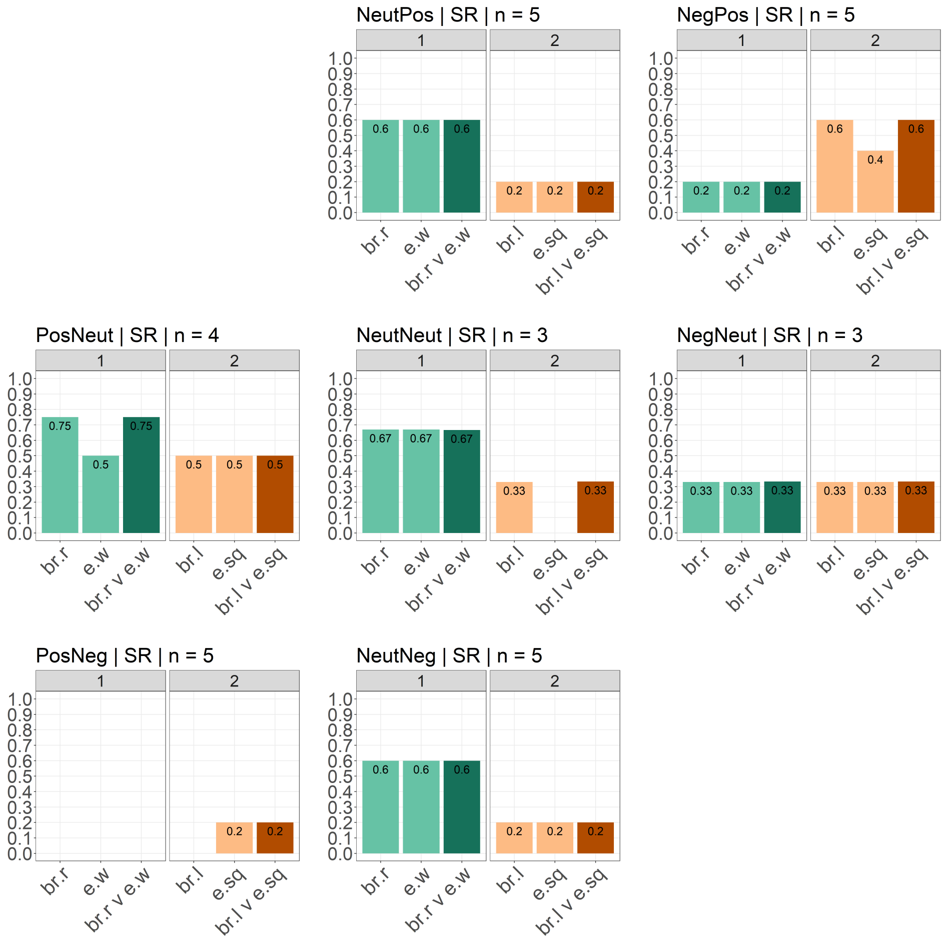

Figure G: Proportions of brow position and eye shape non-manuals per experimental condition in question forms consisting of only a SR, uttered by **participant 03**. Brow raising and eyes wide are presented in green on the left, and brow lowering and eye squint are presented in orange on the right of each condition subplot. The third bar in each subplot indicates the proportion of question forms with either brow raising/lowering or eye widening/squinting, or both. The number of tokens per condition based on which the proportions are calculated are reported in the upper right corner of each bar chart.

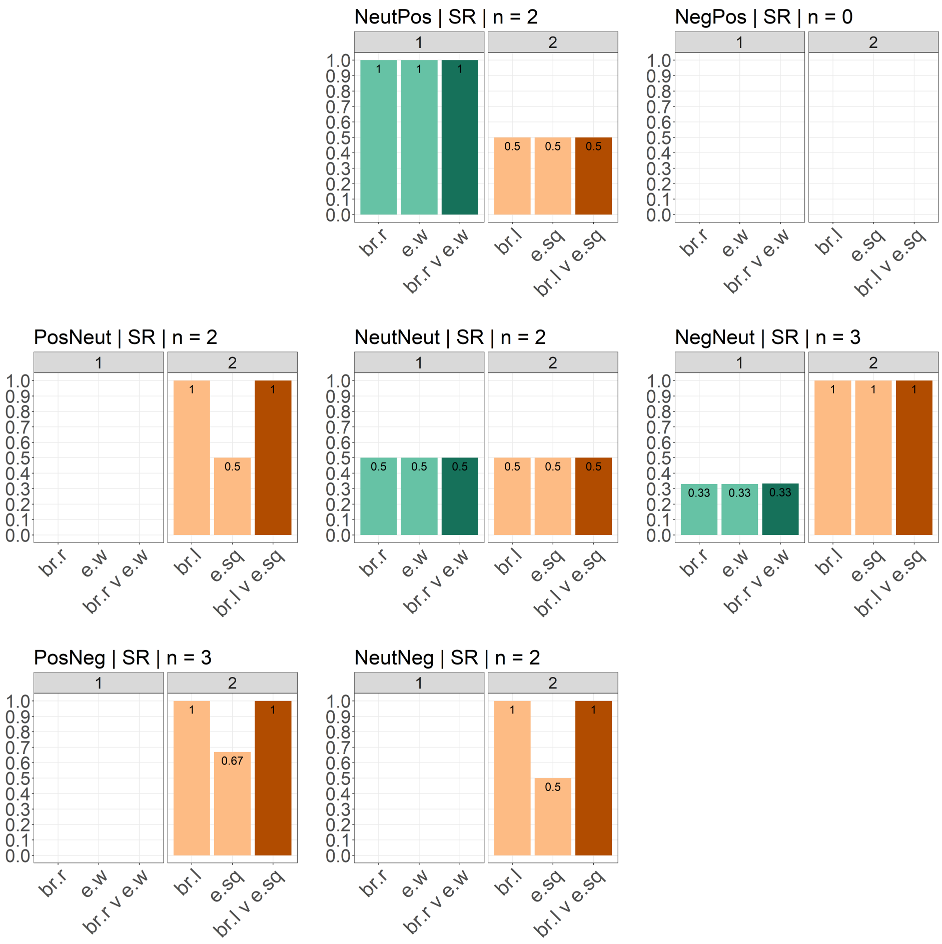

Figure H: Proportions of brow position and eye shape non-manuals per experimental condition in question forms consisting of only a SR, uttered by **participant 05**. Brow raising and eyes wide are presented in green on the left, and brow lowering and eye squint are presented in orange on the right of each condition subplot. The third bar in each subplot indicates the proportion of question forms with either brow raising/lowering or eye widening/squinting, or both. The number of tokens per condition based on which the proportions are calculated are reported in the upper right corner of each bar chart.

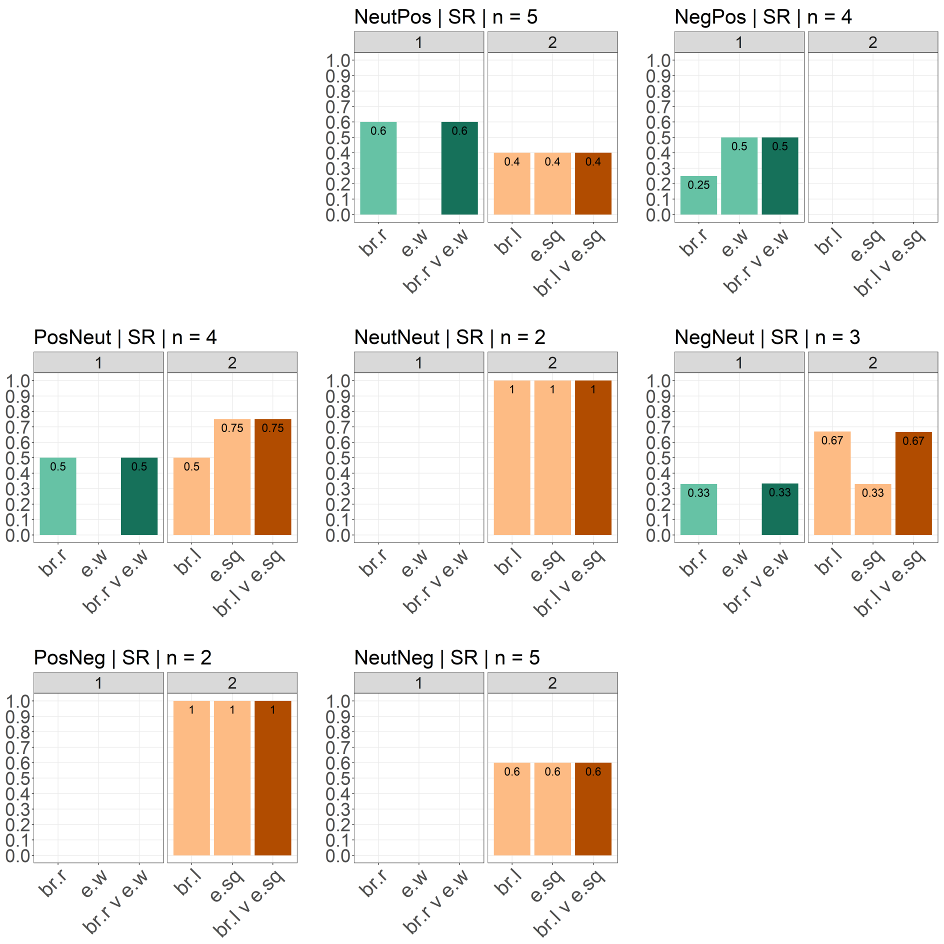

Figure I: Proportions of brow position and eye shape non-manuals per experimental condition in question forms consisting of only a SR, uttered by **participant 06**. Brow raising and eyes wide are presented in green on the left, and brow lowering and eye squint are presented in orange on the right of each condition subplot. The third bar in each subplot indicates the proportion of question forms with either brow raising/lowering or eye widening/squinting, or both. The number of tokens per condition based on which the proportions are calculated are reported in the upper right corner of each bar chart.

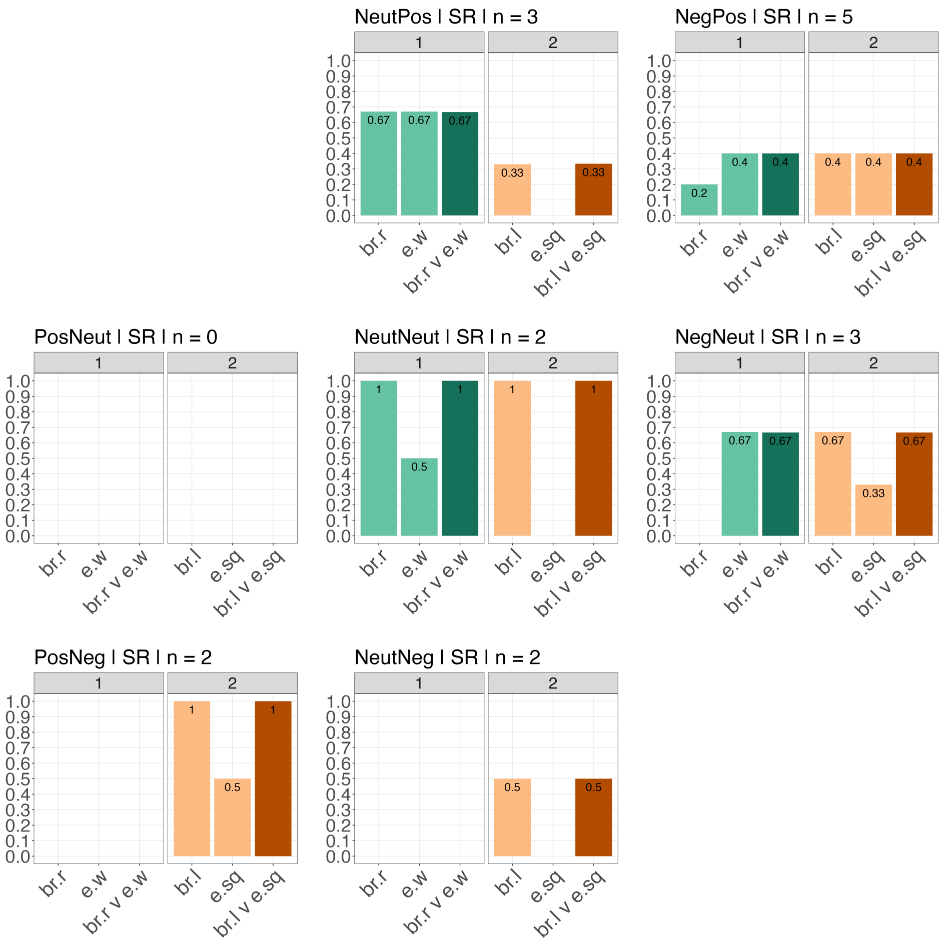

Figure J: Proportions of brow position and eye shape non-manuals per experimental condition in question forms consisting of only a SR, uttered by **participant 07**. Brow raising and eyes wide are presented in green on the left, and brow lowering and eye squint are presented in orange on the right of each condition subplot. The third bar in each subplot indicates the proportion of question forms with either brow raising/lowering or eye widening/squinting, or both. The number of tokens per condition based on which the proportions are calculated are reported in the upper right corner of each bar chart.
