# Supplementary material for: Polar questions in Dutch Sign Language (NGT): A production experiment
Source: PLoS One. 2026 Jul 29;21(7):e0354015. doi: 10.1371/journal.pone.0354015 (PMC13421764; doi:10.1371/journal.pone.0354015)
Supplement: Attachment — Submitted filename: Polar questions in NGT - Response to reviewers.pdf [file pone.0354015.s004.pdf]

Polar questions in Dutch Sign Language: A production study

Response to reviewers

We are grateful to both reviewers for their time considering our manuscript. We thank reviewer 1 for the positive evaluation and valuable and constructive feedback. Below, we list all the points raised by this reviewer and specify how we addressed them.

We are thankful to reviewer 2 for their compliments about our manuscript, which we appreciate very much. Reviewer 2 did not offer any concrete suggestions for improvement of our manuscript, so we do not further address this review below.

In the final part of this document, we also detail how we have ensured that the revised version of our manuscript meets all the journal requirements as mentioned in the editor's letter.

Review 1

Because the review process cannot fully check this, I ask the authors to please ensure that all your earlier publications on polar questions are properly cited, and that any ideas, examples, or content drawn from your previous work are appropriately referenced.

We previously published a short proceedings paper (Oomen & Roelofsen 2023) based on the analysis of a subset of the dataset discussed in the present study (the 68 question forms that involve a headshake). We already referred to this paper in the discussion of inquisitive headshake in the previous version of the manuscript, but we have now added footnotes in the sections 'Sentence radical and tag(s)' and 'Discussion: Non-manuals' to refer to this paper too.

The first version of the manuscript included a figure (3b) that was previously published in Oomen & Roelofsen (2023). We replaced the figure by a different one.

A brief comment on the uploaded examples: they are beautifully done.

Thank you!

Given the length of the manuscript, I recommend considering the removal of information that is repeated in both the results and discussion (e.g., identical statements about numbers or percentages of mouth shrugs and similar).

We have edited the entire paper for conciseness.

In the discussion, you mention multimodal gestalts (Holler & Levinson, 2019). Would it be possible to add what you find to be the most common or frequent multimodal gestalt—i.e.,

combinations of non-manuals—associated with polar questions in each experimental condition? I think this would be very interesting and would improve the study.

We agree that this would be very interesting and we plan to explore this question in future work using machine-learning techniques. At present, based on the analytical techniques we used here, we cannot reliably do this.

One remaining point concerns mouthings. They are annotated in a somewhat unusual way—not as non-manuals but as "special interest"—yet no analysis or results are provided. Please consider either explaining this choice as a limitation or removing the mouthings component entirely from the manuscript.

We have added a note at the end of the section 'Annotating additional elements of interest' to say that we did not systematically analyze these elements and that this can be considered a limitation. However, as we additionally note, we show in the section on questions with an or… tag later on that mouthings do play an important role in this particular question form. Mouthings are thus not entirely ignored in our analyses.

Minor comments:
p. 2 – Research questions:
I suggest revising the formulation of the research questions. As currently phrased, "Which polar question forms exist in NGT and which non-manual markers are used to mark them?" appears to combine two genuinely different questions, and the second cannot naturally be embedded within the first. In addition, the distribution and function of non-manual markers constitute an important empirical contribution of the study (as highlighted in the abstract), so it would be appropriate to present this as a distinct research question.
One possible restructuring would be to keep together those questions that deal with the forms and contexts of use of polar questions in NGT—e.g., "Which polar question forms exist in NGT, and in which contexts can each form be felicitously used?"—and then formulate a separate research question focusing specifically on non-manual marking: for example, "Which non-manual markers co-occur with the different polar question forms, and how do they contribute to their interpretation?" This separation would clarify the analytical aims and better reflect the contributions of the paper.

That is a very good suggestion. We have rephrased the Research Questions as follows in the revised manuscript:

1. Sentence structure

1a. Which sentence structures are used to express polar questions in NGT?

1b. In which kinds of contexts can each sentence structure be felicitously

used? In particular: How do signer expectations and contextual evidence affect the

felicity of the different polar question forms?

1c. What are the communicative functions of different structural elements that are used in polar questions, such as polarity marking and question tags?

2. Non-manuals

2a. Which non-manual markers co-occur with the different sentence structures?

2b. In which kinds of contexts are these non-manual markers used?

2c. What are the communicative functions of these non-manual markers when used in polar questions?

p. 3: You may consider revising the term “prior speaker belief” to “prior belief” (or a similar formulation) so that the terminology is inclusive of signers and not restricted to spoken-language contexts.

Where applicable, we changed it to ‘prior signer belief’, so that we can continue using the abbreviation SB.

p. 5 – Summary of previous literature:
This section provides a very clear and useful overview of earlier work, especially the explanation of Coerts (1992). The discussion effectively motivates your focus on prior speaker belief and contextual evidence, and it convincingly establishes why these aspects are central to your analysis.

Thank you.

p. 6 – “overall, while based on a relatively small dataset …”:
It would be helpful to clarify what exactly is meant by “a relatively small dataset” here. Do you refer to the total number of polar questions, the number of signers, or the amount of contextual material surrounding each question? Since you analyze 197 occurrences of polar question forms (see Table 8), specifying which aspect you consider “small” would make the statement more precise and prevent potential misunderstandings.

We removed this clause. To clarify: the statement was not about our own dataset but about De Vos et al.’s.

p.6. not quite clear here what you mean by “early signers of NGT”. Those who acquired NGT early? When? And why mentioning it here without further explaining it?

We changed “adult native or early signers of NGT” to the more descriptive “adult signers who acquired NGT by the age of four”.

In the sentence, “The situations were loosely based on selected scenarios from a study on biased polar questions in spoken German and English (Domaneschi et al., 2017),” it would be helpful to provide additional detail on the extent to which elements from Domaneschi et al. were adopted or adapted for your stimuli. I also recommend briefly summarizing how the

original study was designed, particularly with respect to whether it considered any visual or co-speech cues accompanying polar questions in spoken languages. Did Domaneschi et al investigate any visual cues accompanying polar questions in spoken languages?

We now clarify in a footnote that Domaneschi et al.'s study was a forced-choice task (picking the best option from a number of sentence forms, given a context description), which used written stimuli. So unlike our study, it was not a production task and no visual cues were involved. Given these major differences, a more in-depth discussion of this study in the ms would take us too far afield.

p.9. "The confederate recordings made during the experimental sessions are not publicly available, but ... (available at https://doi.org/10.21942/uva.26870170)." – this link does not work

Thank you for flagging this. We replaced the link by the correct one.

p. 9 – Description of participant welcoming:
The passage beginning with "Participants were welcomed …" seems unnecessary for the purposes of the methods section, and given the overall length of the paper, this information could be omitted without loss. It can generally be taken for granted that participants were welcomed, so this level of detail feels superfluous. However, this may partly be a matter of stylistic preference.

We have removed the relevant phrase.

p. 9 – Relationship between participants and experimenter/confederates:
It would be helpful to clarify whether the participants and the experimenter or confederates knew each other prior to the study. This information may be relevant, as familiarity can influence communicative behaviour. Including a brief note on this point would strengthen the methodological transparency. ...As I read later in your limitation section "the second confederate had a higher degree of authority, which sometimes led to the use of politeness markers in question forms" – the reader would have appreciated this information earlier...

We added a footnote clarifying that:

"What we mean here is that, in one of the situations, the second confederate had a higher degree of authority in the role they enacted in that particular situation. The degree to which participants were familiar with the person who acted as our second confederate may also have influenced the way in which they phrased their questions. Since the Dutch deaf community is quite tight-knit, all participants had some degree of familiarity with both our confederates. Although further information concerning the exact degree of familiarity could indeed be relevant, we did not collect such information and believe it would be difficult to quantify."

p. 9 – Hearing L2 signer as experimenter:
Consider adding information about the proficiency of the hearing L2 signer in NGT. Many readers remain cautious about the involvement of hearing experimenters in sign-language data collection, and a brief clarification of the signer's competence would help pre-empt concerns and increase confidence in your methodology.

Information about the lead experimenter's signing experience has been added in a footnote.

p. 9 specify what you told the participants while asking "to sign them as naturally as possible." I think it is tricky for some native speakers or signers in front of the camera. And did the participants know aobut the research questions and the goal of the study? Why did you decide to tell or not to tell them this?

We did not explicitly tell participants about the research questions or the goal of the study because this may have influenced their behavior. In our experience, participants felt at ease and interacted in a natural way with the confederates.

p. 10: The sentence, "Participants were instructed to adapt their questions depending on the confederates' utterances, meaning that they might want to ask the same question slightly differently for each variation within a situation," is somewhat unclear. Please clarify what is meant by "adapt" in this context. Specifically, does it refer to adjusting the content, phrasing, stance, mood, or use of non-manual markers? How were participants instructed to interpret the confederates' utterances, and was this adaptation clearly understood by them?

This is clarified in the following sentence:

"To help them get a sense of what we were after, the participants were then shown three examples of variations of the same base question (e.g., ``Is Kim home?''), but with different use of facial expressions and other non-manual markers (e.g., headshake for negation)."

which we now moved from earlier in the paragraph to right after the quoted sentence.

p. 10 The paragraph thanking the participants feels out of place in this section and adds little methodological value. Given the already considerable length of the paper, I recommend deleting or substantially shortening it. While this is a minor issue in itself, its presence—combined with remaining unanswered questions about the actual procedure—creates an imbalance: space is spent on acknowledgments rather than on essential methodological details. Please revise this section to make it more concise and informative. In particular, it would be helpful to provide clearer information about how the sessions unfolded, including the role and necessity of having multiple people present in the room. This will allow readers to fully understand what was done and why.

We removed the paragraph mentioned. As mentioned in the ms, the roles of the two experimenters were:

- *Experimenter 1 (main): provide instructions, control the stimulus presentation, control camera 1.*
- *Experimenter 2: control camera 2 and clapperboard for synchronization between cameras, and keep track of progression through the trials on a whiteboard visible to all.*

It was not possible for one person to carry out all of the tasks above at the same time.

p. 10 It is commendable that the authors have already included the videos of all six participants whose productions were analyzed (https://doi.org/10.21942/uva.21666203), and the link works well. However, it may be helpful to include translations or glosses in these videos for readers who do not understand NGT, in order to make the data more accessible and interpretable for a wider audience of PLOS ONE.

All sentences have been annotated and glossed. These data are available as eaf files here: https://doi.org/10.21942/uva.22737074. The link is included in the paper.

p. 11: It appears that a considerable number of cases were subsequently excluded from the analysis. Could the authors comment on the possible reasons for this? For example, did participants have difficulty understanding the experimental instructions, experience fatigue, or find the productions themselves challenging? Including some discussion or speculation on why these exclusions occurred, as well as how they may have influenced the overall results and interpretation of the study, would strengthen the methodological transparency. Were such exclusions anticipated, or did they exceed expectations?

This is explained in paragraphs 4-5 of the section 'Dataset and data processing'.

p. 11 error here – "For each video file in the dataset, we added annotations in ELAN across a set of 21 newly created tiers tiers."

Error has been fixed.

p. 11 I have never heard of "daughter tiers" :) – but of "children tiers"

Error has been fixed.

p. 12 "We define tags as particular combinations of manual and non-manual markers following the sentence radical (SR), fulfilling a specific semantic function (see Section 5.2 for examples and discussion)." Am I correct in assuming then that non-manual markers alone can not be tags according to this definition?

No, we believe that tags could in principle also be expressed entirely non-manually.

p. 12 I think you should say a couple of words about palm-up before stating here “whether it included a prosodically integrated PALM-UP sign;”

We added a footnote with a brief explanation of the sign/gesture and a relevant reference. In it, we also refer to figures in the ms that illustrate the sign.

p. 12:
I am not fully convinced by the decision to exclude single nods on the basis that they “may be used to mark an intonation phrase boundary and/or to signal emphasis” (Klomp, 2021). Single nods can also serve other functions, such as marking stance (Roseano et al., 2016; Pfänder & Couper-Kiuhlen, 2019), indicating affiliation (Stivers, 2008), or signaling affirmation.

What's relevant for our decision here is that single nods can have functions other than marking polarity. We mention here two such functions, which have been described for head nods in NGT, specifically. While we acknowledge that there may be other possible functions of head nods in NGT, the main point is that in the cases at hand, we do not think that they serve to mark polarity. We slightly rephrased the text to make this point clearer.

Also , it would be helpful to clarify how adjacent or sequential nods were treated in the annotation: for example, if a signer nods, pauses briefly, and then nods again, how did you determine whether this constitutes continuous head nodding versus separate events? Please specify the thresholds or criteria used for distinguishing individual nods from continuous nod sequences. Clearer explanation here would strengthen the transparency and reproducibility of your annotation methodology.

We did not define explicit thresholds. In our experience the difference between multiple single nods and continuous head nodding in our data was always clear-cut.

p. 12 “SRs that were marked for positive and negative polarity were annotated as ‘SR.pos’ and ‘SR.neg’, respectively.” Clarify here how this decision has been done. When a headshake accompanied SR it was marked SR.neg? and same with PU and multiple head nods. Not clear here.

We rephrased this sentence to make clear that one marker (manual or non-manual) sufficed to categorize SRs are positive/negative polarity marked.

p. 13 TOCH tag appear in table 5 for the first time in the paper – explain it here.

and

p. 13 similarly, explain the “confusion tag” HUH. How does it differ from open repair? (Dingemanse, 2024)

and

p. 13 clarify here already what you mean by inquisitive headshake.

The text surrounding Table 5 includes a reference to the results section in which the tags (TOCH, HUH, and inquisitive headshake) are described in detail.

Questions with the confusion tag HUH differ from open repairs in Dingemanse's sense in two ways: first, such questions are not necessarily repairs (they do not necessarily signal a failure in understanding the previous utterance), and secondly, in cases where they do function as repairs, they are not open, because they indicate which specific aspect of the previous utterance was not understood.

p. 15 here it is not clear why mouthings are not included into non-manuals, but it will become clear, but it is the second mention of this and it not clear. Especially while mouthing has been recently shown to be very important marker of (polar and content) questions in BSL (Hodge et al. 2025).

Hodge et al. (2025) show that mouthings play a crucial role in wh-questions in particular. For polar questions, the authors state that "question mouthings were absent for the overwhelming majority of cases". Our study only includes polar questions.

We do discuss mouthings in the ms to some extent, in particular in the discussion of or… *tags, where mouthings play a crucial role.*

p.15. if EXCL were excluded and are not shown, they should not be mentioned here. I suggest to delete the mention of them in "Table 8: Frequency of occurrence of the main question types in each experimental condition. SR = dark grey; SR-T1(-T2) = grey; EMB = diagonally striped; EXCL = not shown (white)."

We have changed the colors in the figure. EXCL is now represented in light gray.

p. 17. You write "Sometimes, there is a small pause between the two sentence parts. We can distinguish six types of tags in our data,..." Clarify here how long is the pause and what makes you think these are sentence parts.

We have not defined a temporal threshold for pauses between SRs and tags, but rather relied on qualitative judgments. SR-tag constructions do not necessarily involve a pause separating the two sentence parts. Other prosodic cues are also mentioned in the text:

"Tags are particular combinations of manual and non-manual markers that follow the SR, making up a separate prosodic unit. Constructions with tags typically display a marked shift in non-manuals between the SR and the tag, with typically multiple non-manual markers that are actively engaged in the SR changing their configuration in the tag. Sometimes, there is a small pause between the two sentence parts."

p. 18 – "Inquisitive headshake":
You write that you "often observed what we call an inquisitive headshake," and describe it as expressing uncertainty and possibly soliciting a response from the addressee. Without having yet consulted your 2023 paper (which I will), it seems that what you label inquisitive may

correspond to what has been described in the literature as pragmatic headshaking(e.g., Johnston 2018; Schoonjans 2014). While inquisitive is an appealing label, it also strikes me as somewhat restrictive: headshakes in both spoken and signed discourse have been shown to serve a range of pragmatic functions—not only requesting information, but also expressing negation, epistemic stance, or other modal, discursive, and interactional meaning.
If your intention is to use inquisitive specifically to denote headshakes that request information or seek a response, that is of course a legitimate terminological choice. However, in that case I recommend situating the term more clearly within the existing literature on pragmatic headshaking and clarifying how your usage relates to (or diverges from) these earlier accounts. This will help readers understand the scope of the category and avoid potential terminological confusion.

Thank you for this point. We have rephrased the text to situate our terminology more clearly in existing literature.

Figures 8–9-10-11:
While your study reports a higher number of non-manual occurrences compared to previous literature, the proportions of these non-manuals within each experimental condition remain relatively low. This discrepancy should be explicitly acknowledged. I recommend that you address this in either the limitations section or in the discussion

We don't think there are previous studies which are similar enough in design to do a concrete quantitative comparison. Moreover, as we mention at the beginning of the non-manuals results section, we are more interested in comparing non-manual proportions across different experimental conditions than interpretating absolute proportions in each condition.

p. 31 – Reference to Hodge et al. (2025):
The discussion of Hodge et al. (2025) is very brief, given the relevance of their contribution to the issues addressed in this paper. I believe their work deserves more detailed engagement—or at least a clearer explanation of how it relates to your analysis—than it currently receives as a footnote.

We agree. We have promoted the discussion of Hodge et al. (2025) from a footnote to the main body of the text, and we have expanded the discussion to make more explicit how their findings for BSL relate to the findings we report in our study.

p. 33, Figure 12:
Please spell out all abbreviations used in the figure within the caption. Providing the full terms will improve readability and ensure that readers can understand the figure without needing to search elsewhere in the manuscript.

We have added explanations of the abbreviations to the captions of Figures 8-12.

p. 35 – Comparison with Spoken Language:
The discussion on p. 35 regarding parallels with spoken language is very compelling, and I

believe it could be developed slightly further. In particular, it would be worth considering whether comparisons with visual cues during speech are a fruitful line of inquiry. Several aspects of your methodology rely on studies originally conducted on spoken language, yet co-speech non-manual gestures do not appear to be taken into account. Johnston (2018), for example, argues that headshake functions in broadly similar ways across signed and spoken modalities. This invites the question of whether head forward might serve an analogous function in spoken Dutch, particularly in polar questions. I encourage the authors to reflect briefly on whether such a cross-modal comparison is theoretically and empirically justified, whether their current methodology could accommodate such an investigation, and to clarify any limitations that might prevent direct comparison. Consider to move the discussion of Weijland (2024) from limitaion and further work to page 35.

We have added a paragraph in the Discussion section "What is the main non-manual marker of polar questions/inquisitiveness?", drawing a connection with work on co-speech gesture which has found that head/body forward often occurs with inquisitive constructions in spoken languages as well. We also discuss a possible functional explanation for this finding, but point out that this may not extend to the case of sign languages. The discussion of Weijland (2024), a pilot study of visual cues in polar questions in spoken Dutch using the same experimental design as the present study for NGT, is still in the "Limitations and Future Work" section at the end of the paper, because this work (a master thesis which we supervised) is really just a pilot study and does not yet provide a fully worked-out analysis of visual cues in polar questions in Dutch.

p. 37 – Use of the term "multimodal communication":
I noticed several instances where you use the term "multimodal communication" to refer specifically to spoken language accompanied by co-speech non-manual gestures, as in the sentence: "In multimodal communication, eyebrow raising has been shown to positively influence participants' perception of the politeness of verbal statements…" (Trees & Manusov, 1998). It is unclear whether this terminology is being used intentionally to distinguish spoken language plus gesture from signed language, which you may be conceptualizing as not multimodal because it is expressed within a single visual-gestural modality.
However, this distinction may not be theoretically aligned with broader usage in linguistics. In much of the recent literature, both spoken and signed languages are considered multimodal—spoken language integrates acoustic and visual cues, while signed language integrates manual and non-manual articulators, various channels and plus mouthing or fingerspelling which have a cognitive representation in spoken or written language (see further Cohn & Shilperoord 2024 for a broader conceptualization of multimodality). Therefore, the phrase "multimodal communication" as used here risks sounding imprecise or even misleading.
I recommend clarifying your intended distinction, and considering alternative phrasing (e.g., "spoken language with co-speech gestures") to avoid implying that signed languages are not inherently multimodal.

Thank you for this point. We do not mean to imply that sign languages are strictly unimodal, so we have changed the instances of 'multimodal communication' into 'co-speech gesture' or other more fitting phrases.

Sections 8.xx.xx – The discussion in these sections could be substantially tightened. At present, the text is overly long, contains noticeable repetition, and at several points engages in speculative interpretations that seem to rest on a very small number of occurrences or examples.

The entire manuscript has been edited for conciseness.

References:

The link/doi does not work: Oomen, M., & Roelofsen, F. (2023). Biased polar question forms in sign language of the netherlands (ngt): Two
functions of headshake. FEAST, 5, 156–168. https://doi.org/10.31009/FEAST.i5.13

We checked the link and it works.

Journal requirements

1. Please ensure that your manuscript meets PLOS ONE's style requirements, including those for file naming. We used the PLOS ONE tex template for our resubmission and made the necessary edits to ensure that our manuscript is in line with the journal's style requirements.

Figures have been renamed to meet the criteria.

2. Your ethics statement should only appear in the Methods section of your manuscript.

Done.

3. We noted in your submission details that a portion of your manuscript may have been presented or published elsewhere. [...] Please clarify whether this [conference proceeding or publication] was peer-reviewed and formally published. If this work was previously peer-reviewed and published, in the cover letter please provide the reason that this work does not constitute dual publication and should be included in the current manuscript.

None of the mentioned short conference proceedings papers were peer-reviewed.

4. We note that the grant information you provided in the 'Funding Information' and 'Financial Disclosure' sections do not match. When you resubmit, please ensure that you provide the correct grant numbers for the awards you received for your study in the 'Funding Information' section.

   We cannot find a 'Financial Disclosure' section in the submission portal. The correct funding information is as follows: Dutch Research Council (NWO) Vici, grant number VI.C.201.014 (PI Floris Roelofsen) Dutch Research Council (NWO) Veni, grant number VI.VENI.211C.052 (PI Marloes Oomen) We entered this information in the 'Funding Information' section on the portal, as requested.

5. Please amend either the title on the online submission form (via Edit Submission) or the title in the manuscript so that they are identical.

Done.

6. Please update your submission to use the PLOS LaTeX template.

Done.

7. Your abstract cannot contain citations. Please only include citations in the body text of the manuscript, and ensure that they remain in ascending numerical order on first mention.

Done.

8. We note that Figure(s) 1, 2, Appendix A in your submission contain copyrighted images. All PLOS content is published under the Creative Commons Attribution License (CC BY 4.0), which means that the manuscript, images, and Supporting Information files will be freely available online, and any third party is permitted to access, download, copy, distribute, and use these materials in any way, even commercially, with proper attribution. We require you to either (1) present written permission from the copyright holder to publish these figures specifically under the CC BY 4.0 license, or (2) remove the figures from your submission:

We are the copyright holders of these images. We previously included the relevant images in a document we archived and published under a CC BY 4.0 license in our university's repository: https://doi.org/10.21942/uva.21701954. Given this, we believe it is not necessary to request permission for publication nor to include reprint statements in the paper.

9. We note that Figure (3, 4, 5, 6, and 7, Oomen & Roelofsen 2023.pdf, Esselink, Oomen & Roelofsen 2023.pdf, Oomen, Essenlink, De Ronde & Roelofsen 2023.pdf, Polar questions in NGT.zip,) includes an image of a [patient / participant / in the study].

We reached out to all five participants shown in the images to request their consent for the publication of these images under the terms of a CC BY license. All participants gave their written informed consent. We have added the sentence "Participants who are shown in images in this article gave their written informed consent for publication of these images under the terms of a CC BY license" to the ethics statement in the methods section. The other documents mentioned were added as supporting files for the editorial office/reviewers only. They will not need to be published in PLOS ONE.

10. We note that there is identifying data in the Supporting Information file <FEC 202201 Roelofsen.pdf>. Due to the inclusion of these potentially identifying data, we have removed this file from your file inventory. Prior to sharing human research participant data, authors should consult with an ethics committee to ensure data are shared in accordance with participant consent and all applicable local laws.

This file does not include human research participant data. It is an approval letter from the ethics committee of our university's faculty of science, which includes the contact information of the chair of the board. We attached the letter as proof that we received approval for our study from the ethics board. To avoid the document being flagged again, we removed it now.

11. Please include captions for your Supporting Information files at the end of your manuscript, and update any in-text citations to match accordingly.

Done.

12. If the reviewer comments include a recommendation to cite specific previously published works, please review and evaluate these publications to determine whether they are relevant and should be cited. There is no requirement to cite these works unless the editor has indicated otherwise.

Noted. We have referenced some of the publications mentioned by reviewer 1 in the revised version of the manuscript (see next point).

13. Please review your reference list to ensure that it is complete and correct. If you have cited papers that have been retracted, please include the rationale for doing so in the manuscript text, or remove these references and replace them with relevant current references. Any changes to the reference list should be mentioned in the rebuttal letter that accompanies your revised manuscript. If you need to cite a retracted article, indicate the article's retracted status in the References list and also include a citation and full reference for the retraction notice.

We have reviewed the reference list and we do not cite any retracted papers. We have added the following references in response to comments made by reviewer 1:

[38] Cooperrider et al. (2018)

[48] Johnston (2018)

[49] Pfau (2015)

[58] Li (2014)

[59] Rasmussen (2014)

[60] Kendrick (2015)

[61] Trujillo & Holler (2021)

We have also switched to Vancouver citation and reference styles.
