# Supplementary material for: Polar questions in Dutch Sign Language (NGT): A production experiment
Source: PLoS One. 2026 Jul 29;21(7):e0354015. doi: 10.1371/journal.pone.0354015 (PMC13421764; doi:10.1371/journal.pone.0354015)
Supplement: Peer review history

# Peer review: Decision Letter 0

**DOI:** 10.1371/journal.pone.0354015.r001

**Authors:** Mary Diane Clark

17 Dec 2025

PONE-D-25-55016

Polar questions in Dutch Sign Language (NGT): A production experiment

PLOS One

Dear Dr. Oomen,

Thank you for submitting your manuscript to PLOS ONE. After careful consideration, we feel that it has merit but does not fully meet PLOS ONE’s publication criteria as it currently stands. Therefore, we invite you to submit a revised version of the manuscript that addresses the points raised during the review process.

Please submit your revised manuscript by Jan 31 2026 11:59PM. If you will need more time than this to complete your revisions, please reply to this message or contact the journal office at plosone@plos.org. When you're ready to submit your revision, log on to https://www.editorialmanager.com/pone/ and select the 'Submissions Needing Revision' folder to locate your manuscript file.

Thank you for the interesting and conceptually driven paper. The reviewers have given you detailed feedback about  how to make it a bit better. In addition, please look at the publication guidelines for Plos One's citations and references.  Yours do not follow their style.

Please include the following items when submitting your revised manuscript:

- A letter that responds to each point raised by the academic editor and reviewer(s). You should upload this letter as a separate file labeled 'Response to Reviewers'.
- A marked-up copy of your manuscript that highlights changes made to the original version. You should upload this as a separate file labeled 'Revised Manuscript with Track Changes'.
- An unmarked version of your revised paper without tracked changes. You should upload this as a separate file labeled 'Manuscript'.

-->If you would like to make changes to your financial disclosure, please include your updated statement in your cover letter. Guidelines for resubmitting your figure files are available below the reviewer comments at the end of this letter.

If applicable, we recommend that you deposit your laboratory protocols in protocols.io to enhance the reproducibility of your results. Protocols.io assigns your protocol its own identifier (DOI) so that it can be cited independently in the future. For instructions see: https://journals.plos.org/plosone/s/submission-guidelines#loc-laboratory-protocols. Additionally, PLOS ONE offers an option for publishing peer-reviewed Lab Protocol articles, which describe protocols hosted on protocols.io. Read more information on sharing protocols at https://plos.org/protocols?utm_medium=editorial-email&utm_source=authorletters&utm_campaign=protocols.

We look forward to receiving your revised manuscript.

Kind regards,

Mary Diane Clark, PhD

Academic Editor

PLOS One

Journal Requirements:

When submitting your revision, we need you to address these additional requirements.

1. Please ensure that your manuscript meets PLOS ONE's style requirements, including those for file naming. The PLOS ONE style templates can be found at https://journals.plos.org/plosone/s/file?id=wjVg/PLOSOne_formatting_sample_main_body.pdf and https://journals.plos.org/plosone/s/file?id=ba62/PLOSOne_formatting_sample_title_authors_affiliations.pdf

2. Your ethics statement should only appear in the Methods section of your manuscript. If your ethics statement is written in any section besides the Methods, please delete it from any other section.

3. We noted in your submission details that a portion of your manuscript may have been presented or published elsewhere. We have previously published a small proceedings paper discussing a subset of the data discussed in the manuscript. In that paper, we focus specifically on the distribution and functions of headshake in the data set. The submitted ms is based on a larger data set, has a different main focus, and includes considerably more in-depth analysis and discussion. Figure 3b in the ms was also published in the proceedings paper.

Reference:

Oomen, Marloes and Floris Roelofsen. 2023. Biased polar question forms in Sign Language of the Netherlands (NGT): Two functions of headshake. FEAST 5: 156-168. https://doi.org/10.31009/FEAST.i5.13

We have furthermore used the study described in the ms as a case study in a couple of methodological papers focusing on the capturing, analysis and/or annotation of non-manual markers. These papers include a brief description fo the study as background information. Some information in the methods sections of the papers show a small degree of overlap with information presented in different parts of the methods section of the submitted ms. The papers also include some video stills from the data set analyzed in this paper, although they are different from the ones included in the ms. Given their methodological focus, these papers do not discuss results included in the ms.

Oomen, Marloes, Lyke Esselink, Tobias de Ronde & Floris Roelofsen. 2023. First steps towards a procedure for annotating non-manual markers in sign language. Proceedings of North East Linguistic Society (NELS) 53, 256-266. Preprint DOI: https://doi.org/10.13140/RG.2.2.31312.28169

Esselink, Lyke, Marloes Oomen, & Floris Roelofsen. 2023. Exploring new methods for measuring, analyzing, and visualizing

facial expressions. FEAST. Formal and Experimental Advances in Sign language Theory, 5, 35–48. https://doi.org/10.31009/FEAST.i5.04.

Please clarify whether this [conference proceeding or publication] was peer-reviewed and formally published. If this work was previously peer-reviewed and published, in the cover letter please provide the reason that this work does not constitute dual publication and should be included in the current manuscript.

4. We note that the grant information you provided in the ‘Funding Information’ and ‘Financial Disclosure’ sections do not match.

When you resubmit, please ensure that you provide the correct grant numbers for the awards you received for your study in the ‘Funding Information’ section.

5. Please amend either the title on the online submission form (via Edit Submission) or the title in the manuscript so that they are identical.

6. Please update your submission to use the PLOS LaTeX template. The template and more information on our requirements for LaTeX submissions can be found at http://journals.plos.org/plosone/s/latex.

7. Your abstract cannot contain citations. Please only include citations in the body text of the manuscript, and ensure that they remain in ascending numerical order on first mention.

8. We note that Figure(s) 1, 2, Appendix A in your submission contain copyrighted images. All PLOS content is published under the Creative Commons Attribution License (CC BY 4.0), which means that the manuscript, images, and Supporting Information files will be freely available online, and any third party is permitted to access, download, copy, distribute, and use these materials in any way, even commercially, with proper attribution. For more information, see our copyright guidelines: http://journals.plos.org/plosone/s/licenses-and-copyright.

We require you to either (1) present written permission from the copyright holder to publish these figures specifically under the CC BY 4.0 license, or (2) remove the figures from your submission:

a. You may seek permission from the original copyright holder of Figure(s) 1, 2, Appendix A to publish the content specifically under the CC BY 4.0 license.

We recommend that you contact the original copyright holder with the Content Permission Form (http://journals.plos.org/plosone/s/file?id=7c09/content-permission-form.pdf) and the following text:

“I request permission for the open-access journal PLOS ONE to publish XXX under the Creative Commons Attribution License (CCAL) CC BY 4.0 (http://creativecommons.org/licenses/by/4.0/). Please be aware that this license allows unrestricted use and distribution, even commercially, by third parties. Please reply and provide explicit written permission to publish XXX under a CC BY license and complete the attached form.”

Please upload the completed Content Permission Form or other proof of granted permissions as an "Other" file with your submission.

In the figure caption of the copyrighted figure, please include the following text: “Reprinted from [ref] under a CC BY license, with permission from [name of publisher], original copyright [original copyright year].”

b. If you are unable to obtain permission from the original copyright holder to publish these figures under the CC BY 4.0 license or if the copyright holder’s requirements are incompatible with the CC BY 4.0 license, please either i) remove the figure or ii) supply a replacement figure that complies with the CC BY 4.0 license. Please check copyright information on all replacement figures and update the figure caption with source information. If applicable, please specify in the figure caption text when a figure is similar but not identical to the original image and is therefore for illustrative purposes only.

9. We note that Figure (3, 4, 5, 6, and 7, Oomen & Roelofsen 2023.pdf, Esselink, Oomen & Roelofsen 2023.pdf, Oomen, Essenlink, De Ronde & Roelofsen 2023.pdf, Polar questions in NGT.zip,) includes an image of a [patient / participant / in the study].

As per the PLOS ONE policy (http://journals.plos.org/plosone/s/submission-guidelines#loc-human-subjects-research) on papers that include identifying, or potentially identifying, information, the individual(s) or parent(s)/guardian(s) must be informed of the terms of the PLOS open-access (CC-BY) license and provide specific permission for publication of these details under the terms of this license. Please download the Consent Form for Publication in a PLOS Journal (http://journals.plos.org/plosone/s/file?id=8ce6/plos-consent-form-english.pdf). The signed consent form should not be submitted with the manuscript, but should be securely filed in the individual's case notes. Please amend the methods section and ethics statement of the manuscript to explicitly state that the patient/participant has provided consent for publication: “The individual in this manuscript has given written informed consent (as outlined in PLOS consent form) to publish these case details”.

If you are unable to obtain consent from the subject of the photograph, you will need to remove the figure and any other textual identifying information or case descriptions for this individual.

10. We note that there is identifying data in the Supporting Information file <FEC 2022-01 Roelofsen.pdf>. Due to the inclusion of these potentially identifying data, we have removed this file from your file inventory. Prior to sharing human research participant data, authors should consult with an ethics committee to ensure data are shared in accordance with participant consent and all applicable local laws.

Data sharing should never compromise participant privacy. It is therefore not appropriate to publicly share personally identifiable data on human research participants. The following are examples of data that should not be shared:

-Name, initials, physical address

-Ages more specific than whole numbers

-Internet protocol (IP) address

-Specific dates (birth dates, death dates, examination dates, etc.)

-Contact information such as phone number or email address

-Location data

-ID numbers that seem specific (long numbers, include initials, titled “Hospital ID”) rather than random (small numbers in numerical order)

Data that are not directly identifying may also be inappropriate to share, as in combination they can become identifying. For example, data collected from a small group of participants, vulnerable populations, or private groups should not be shared if they involve indirect identifiers (such as sex, ethnicity, location, etc.) that may risk the identification of study participants.

Additional guidance on preparing raw data for publication can be found in our Data Policy (https://journals.plos.org/plosone/s/data-availability#loc-human-research-participant-data-and-other-sensitive-data) and in the following article: http://www.bmj.com/content/340/bmj.c181.long.

Please remove or anonymize all personal information (<specific identifying information in file to be removed>), ensure that the data shared are in accordance with participant consent, and re-upload a fully anonymized data set. Please note that spreadsheet columns with personal information must be removed and not hidden as all hidden columns will appear in the published file.

11. Please include captions for your Supporting Information files at the end of your manuscript, and update any in-text citations to match accordingly. Please see our Supporting Information guidelines for more information: http://journals.plos.org/plosone/s/supporting-information.

12. If the reviewer comments include a recommendation to cite specific previously published works, please review and evaluate these publications to determine whether they are relevant and should be cited. There is no requirement to cite these works unless the editor has indicated otherwise.

13. Please review your reference list to ensure that it is complete and correct. If you have cited papers that have been retracted, please include the rationale for doing so in the manuscript text, or remove these references and replace them with relevant current references. Any changes to the reference list should be mentioned in the rebuttal letter that accompanies your revised manuscript. If you need to cite a retracted article, indicate the article’s retracted status in the References list and also include a citation and full reference for the retraction notice.

Additional Editor Comments :

Thank you for submitting this article to Plos One. It is an interesting and well written manuscript. The reviewers have given you some minor feedback to make the paper a bit stronger. Plos One use Vancouver citation and reference styles -- therefore the citations in the body of the paper are numbers and that is how they are entered into the reference section.

[Note: HTML markup is below. Please do not edit.]

Reviewers' comments:

Reviewer's Responses to Questions

**Comments to the Author**

1. Is the manuscript technically sound, and do the data support the conclusions?

The manuscript must describe a technically sound piece of scientific research with data that supports the conclusions. Experiments must have been conducted rigorously, with appropriate controls, replication, and sample sizes. The conclusions must be drawn appropriately based on the data presented.

Reviewer #1: Yes

Reviewer #2: Yes

**********

2. Has the statistical analysis been performed appropriately and rigorously?

Reviewer #1: Yes

Reviewer #2: Yes

**********

3. Have the authors made all data underlying the findings in their manuscript fully available?

The PLOS Data policy requires authors to make all data underlying the findings described in their manuscript fully available without restriction, with rare exception (please refer to the Data Availability Statement in the manuscript PDF file). The data should be provided as part of the manuscript or its supporting information, or deposited to a public repository. For example, in addition to summary statistics, the data points behind means, medians and variance measures should be available. If there are restrictions on publicly sharing data—e.g. participant privacy or use of data from a third party—those must be specified.

Reviewer #1: Yes

Reviewer #2: Yes

**********

4. Is the manuscript presented in an intelligible fashion and written in standard English?

PLOS ONE does not copyedit accepted manuscripts, so the language in submitted articles must be clear, correct, and unambiguous. Any typographical or grammatical errors should be corrected at revision, so please note any specific errors here.

Reviewer #1: Yes

Reviewer #2: Yes

**********

5. Review Comments to the Author

Please use the space provided to explain your answers to the questions above. You may also include additional comments for the author, including concerns about dual publication, research ethics, or publication ethics. (Please upload your review as an attachment if it exceeds 20,000 characters)

Reviewer #1: Revising the manuscript for PLOS ONE “Polar questions in Dutch Sign Language (NGT): A production experiment”( PONE-D-25-55016)

Overall impression:

Thank you for the opportunity to review your manuscript for PLOS ONE, “Polar questions in Dutch Sign Language (NGT): A production experiment” (PONE-D-25-55016). I found it to be a well-written, clear, and methodologically solid study with very interesting results. The manuscript provides convincing evidence that eyebrow raising is not in fact a marker of polar-questionhood in NGT. I especially appreciated your explanation for why earlier literature reached a different conclusion: an out-of-the-blue polar question may involve raised eyebrows not because this non-manual signals questionhood, but because introducing a new conversational topic strongly promotes eyebrow raising while eyebrow lowering is not similarly promoted. Your finding that head forward is the most consistently used non-manual marker in polar questions across experimental conditions is important and clearly demonstrated.

I think the study is timely—especially following recent corpus study on wh-questions in BSL by Hodge et al. (2025)—in contributing different data and more refined non-manual annotation to reassess traditional claims about question marking in sign languages. Although the manuscript is somewhat long, the discussion is strong.

I very much liked the study. Overall, I suggest minor revisions. Although I raise several points, they rather concern clarification, additional explanation, or reorganizing content; I am not asking for any major methodological or conceptual changes.

Because the review process cannot fully check this, I ask the authors to please ensure that all your earlier publications on polar questions are properly cited, and that any ideas, examples, or content drawn from your previous work are appropriately referenced.

A brief comment on the uploaded examples: they are beautifully done.

Given the length of the manuscript, I recommend considering the removal of information that is repeated in both the results and discussion (e.g., identical statements about numbers or percentages of mouth shrugs and similar).

In the discussion, you mention multimodal gestalts (Holler & Levinson, 2019). Would it be possible to add what you find to be the most common or frequent multimodal gestalt—i.e., combinations of non-manuals—associated with polar questions in each experimental condition? I think this would be very interesting and would improve the study.

One remaining point concerns mouthings. They are annotated in a somewhat unusual way—not as non-manuals but as “special interest”—yet no analysis or results are provided. Please consider either explaining this choice as a limitation or removing the mouthings component entirely from the manuscript.

Thank you again for this insightful and very valuable contribution. I look forward to seeing the revised version.

Minor comments:

p. 2 – Research questions:

I suggest revising the formulation of the research questions. As currently phrased, “Which polar question forms exist in NGT and which non-manual markers are used to mark them?” appears to combine two genuinely different questions, and the second cannot naturally be embedded within the first. In addition, the distribution and function of non-manual markers constitute an important empirical contribution of the study (as highlighted in the abstract), so it would be appropriate to present this as a distinct research question.

One possible restructuring would be to keep together those questions that deal with the forms and contexts of use of polar questions in NGT—e.g., “Which polar question forms exist in NGT, and in which contexts can each form be felicitously used?”—and then formulate a separate research question focusing specifically on non-manual marking: for example, “Which non-manual markers co-occur with the different polar question forms, and how do they contribute to their interpretation?” This separation would clarify the analytical aims and better reflect the contributions of the paper.

p. 3:

You may consider revising the term “prior speaker belief” to “prior belief” (or a similar formulation) so that the terminology is inclusive of signers and not restricted to spoken-language contexts.

p. 5 – Summary of previous literature:

This section provides a very clear and useful overview of earlier work, especially the explanation of Coerts (1992). The discussion effectively motivates your focus on prior speaker belief and contextual evidence, and it convincingly establishes why these aspects are central to your analysis.

p. 6 – “overall, while based on a relatively small dataset …”:

It would be helpful to clarify what exactly is meant by “a relatively small dataset” here. Do you refer to the total number of polar questions, the number of signers, or the amount of contextual material surrounding each question? Since you analyze 197 occurrences of polar question forms (see Table 8), specifying which aspect you consider “small” would make the statement more precise and prevent potential misunderstandings.

p.6. not quite clear here what you mean by “early signers of NGT”. Those who acquired NGT early? When? And why mentioning it here without further explaining it?

In the sentence, “The situations were loosely based on selected scenarios from a study on biased polar questions in spoken German and English (Domaneschi et al., 2017),” it would be helpful to provide additional detail on the extent to which elements from Domaneschi et al. were adopted or adapted for your stimuli. I also recommend briefly summarizing how the original study was designed, particularly with respect to whether it considered any visual or co-speech cues accompanying polar questions in spoken languages. Did Domaneschi et al investigate any visual cues accompanying polar questions in spoken languages?

p.9. “The confederate recordings made during the experimental sessions are not publicly available, but ... (available at https://doi.org/10.21942/uva.26870170).“ – this link does not work

p. 9 – Description of participant welcoming:

The passage beginning with “Participants were welcomed …” seems unnecessary for the purposes of the methods section, and given the overall length of the paper, this information could be omitted without loss. It can generally be taken for granted that participants were welcomed, so this level of detail feels superfluous. However, this may partly be a matter of stylistic preference.

p. 9 – Relationship between participants and experimenter/confederates:

It would be helpful to clarify whether the participants and the experimenter or confederates knew each other prior to the study. This information may be relevant, as familiarity can influence communicative behaviour. Including a brief note on this point would strengthen the methodological transparency. ...As I read later in your limitation section “the second confederate had a higher degree of authority, which sometimes led to the use of politeness markers in question forms“ – the reader would have appreciated this information earlier...

p. 9 – Hearing L2 signer as experimenter:

Consider adding information about the proficiency of the hearing L2 signer in NGT. Many readers remain cautious about the involvement of hearing experimenters in sign-language data collection, and a brief clarification of the signer’s competence would help pre-empt concerns and increase confidence in your methodology.

p. 9 specify what you told the participants while asking “to sign them as naturally as possible.” I think it is tricky for some native speakers or signers in front of the camera. And did the participants know aobut the research questions and the goal of the study? Why did you decide to tell or not to tell them this?

p. 10:

The sentence, “Participants were instructed to adapt their questions depending on the confederates’ utterances, meaning that they might want to ask the same question slightly differently for each variation within a situation,” is somewhat unclear. Please clarify what is meant by “adapt” in this context. Specifically, does it refer to adjusting the content, phrasing, stance, mood, or use of non-manual markers? How were participants instructed to interpret the confederates’ utterances, and was this adaptation clearly understood by them?

p. 10 The paragraph thanking the participants feels out of place in this section and adds little methodological value. Given the already considerable length of the paper, I recommend deleting or substantially shortening it. While this is a minor issue in itself, its presence—combined with remaining unanswered questions about the actual procedure—creates an imbalance: space is spent on acknowledgments rather than on essential methodological details.

Please revise this section to make it more concise and informative. In particular, it would be helpful to provide clearer information about how the sessions unfolded, including the role and necessity of having multiple people present in the room. This will allow readers to fully understand what was done and why.

p. 10 It is commendable that the authors have already included the videos of all six participants whose productions were analyzed (https://doi.org/10.21942/uva.21666203), and the link works well. However, it may be helpful to include translations or glosses in these videos for readers who do not understand NGT, in order to make the data more accessible and interpretable for a wider audience of PLOS ONE.

p. 11:

It appears that a considerable number of cases were subsequently excluded from the analysis. Could the authors comment on the possible reasons for this? For example, did participants have difficulty understanding the experimental instructions, experience fatigue, or find the productions themselves challenging? Including some discussion or speculation on why these exclusions occurred, as well as how they may have influenced the overall results and interpretation of the study, would strengthen the methodological transparency. Were such exclusions anticipated, or did they exceed expectations?

p. 11 error here – “For each video file in the dataset, we added annotations in ELAN across a set of 21 newly created tiers tiers.“

p. 11 I have never heard of “daughter tiers” :) – but of “children tiers”

p. 12 “We define tags as particular combinations of manual and non-manual markers following the sentence radical (SR), fulfilling a specific semantic function (see Section 5.2 for examples and discussion).“ Am I correct in assuming then that non-manual markers alone can not be tags according to this definition?

p. 12 I think you should say a couple of words about palm-up before stating here “whether it included a prosodically integrated PALM-UP sign;”

p. 12:

I am not fully convinced by the decision to exclude single nods on the basis that they “may be used to mark an intonation phrase boundary and/or to signal emphasis” (Klomp, 2021). Single nods can also serve other functions, such as marking stance (Roseano et al., 2016; Pfänder & Couper-Kiuhlen, 2019), indicating affiliation (Stivers, 2008), or signaling affirmation.

Also , it would be helpful to clarify how adjacent or sequential nods were treated in the annotation: for example, if a signer nods, pauses briefly, and then nods again, how did you determine whether this constitutes continuous head nodding versus separate events? Please specify the thresholds or criteria used for distinguishing individual nods from continuous nod sequences. Clearer explanation here would strengthen the transparency and reproducibility of your annotation methodology.

p. 12 “SRs that were marked for positive and negative polarity were annotated

as ‘SR.pos’ and ‘SR.neg’, respectively.” Clarify here how this decision has been done. When a headshake accompanied SR it was marked SR.neg? and same with PU and multiple head nods. Not clear here.

p. 13 TOCH tag appear in table 5 for the first time in the paper – explain it here.

p. 13 similarly, explain the “confusion tag” HUH. How does it differ from open repair? (Dingemanse, 2024)

p. 13 clarify here already what you mean by inquisitive headshake.

p. 15 here it is not clear why mouthings are not included into non-manuals, but it will become clear, but it is the second mention of this and it not clear. Especially while mouthing has been recently shown to be very important marker of (polar and content) questions in BSL (Hodge et al. 2025).

p.15. if EXCL were excluded and are not shown, they should not be mentioned here. I suggest to delete the mention of them in “Table 8: Frequency of occurrence of the main question types in each experimental condition. SR = dark grey; SR-T1(-T2) = grey; EMB = diagonally striped; EXCL = not shown (white).”

p. 17. You write “Sometimes, there is a small pause between the two sentence parts. We can distinguish six types of tags in our data,...“ Clarify here how long is the pause and what makes you think these are sentence parts.

p. 18 – “Inquisitive headshake”:

You write that you “often observed what we call an inquisitive headshake,” and describe it as expressing uncertainty and possibly soliciting a response from the addressee. Without having yet consulted your 2023 paper (which I will), it seems that what you label inquisitive may correspond to what has been described in the literature as pragmatic headshaking(e.g., Johnston 2018; Schoonjans 2014). While inquisitive is an appealing label, it also strikes me as somewhat restrictive: headshakes in both spoken and signed discourse have been shown to serve a range of pragmatic functions—not only requesting information, but also expressing negation, epistemic stance, or other modal, discursive, and interactional meaning.

If your intention is to use inquisitive specifically to denote headshakes that request information or seek a response, that is of course a legitimate terminological choice. However, in that case I recommend situating the term more clearly within the existing literature on pragmatic headshaking and clarifying how your usage relates to (or diverges from) these earlier accounts. This will help readers understand the scope of the category and avoid potential terminological confusion.

Figures 8–9-10-11:

While your study reports a higher number of non-manual occurrences compared to previous literature, the proportions of these non-manuals within each experimental condition remain relatively low. This discrepancy should be explicitly acknowledged. I recommend that you address this in either the limitations section or in the discussion

p. 31 – Reference to Hodge et al. (2025):

The discussion of Hodge et al. (2025) is very brief, given the relevance of their contribution to the issues addressed in this paper. I believe their work deserves more detailed engagement—or at least a clearer explanation of how it relates to your analysis—than it currently receives as a footnote.

p. 33, Figure 12:

Please spell out all abbreviations used in the figure within the caption. Providing the full terms will improve readability and ensure that readers can understand the figure without needing to search elsewhere in the manuscript.

p. 35 – Comparison with Spoken Language:

The discussion on p. 35 regarding parallels with spoken language is very compelling, and I believe it could be developed slightly further. In particular, it would be worth considering whether comparisons with visual cues during speech are a fruitful line of inquiry. Several aspects of your methodology rely on studies originally conducted on spoken language, yet co-speech non-manual gestures do not appear to be taken into account. Johnston (2018), for example, argues that headshake functions in broadly similar ways across signed and spoken modalities. This invites the question of whether head forward might serve an analogous function in spoken Dutch, particularly in polar questions. I encourage the authors to reflect briefly on whether such a cross-modal comparison is theoretically and empirically justified, whether their current methodology could accommodate such an investigation, and to clarify any limitations that might prevent direct comparison. Consider to move the discussion of Weijland (2024) from limitaion and further work to page 35.

p. 37 – Use of the term “multimodal communication”:

I noticed several instances where you use the term “multimodal communication” to refer specifically to spoken language accompanied by co-speech non-manual gestures, as in the sentence: “In multimodal communication, eyebrow raising has been shown to positively influence participants’ perception of the politeness of verbal statements…” (Trees & Manusov, 1998). It is unclear whether this terminology is being used intentionally to distinguish spoken language plus gesture from signed language, which you may be conceptualizing as not multimodal because it is expressed within a single visual-gestural modality.

However, this distinction may not be theoretically aligned with broader usage in linguistics. In much of the recent literature, both spoken and signed languages are considered multimodal—spoken language integrates acoustic and visual cues, while signed language integrates manual and non-manual articulators, various channels and plus mouthing or fingerspelling which have a cognitive representation in spoken or written language (see further Cohn & Shilperoord 2024 for a broader conceptualization of multimodality). Therefore, the phrase “multimodal communication” as used here risks sounding imprecise or even misleading.

I recommend clarifying your intended distinction, and considering alternative phrasing (e.g., “spoken language with co-speech gestures”) to avoid implying that signed languages are not inherently multimodal.

Sections 8.xx.xx – The discussion in these sections could be substantially tightened. At present, the text is overly long, contains noticeable repetition, and at several points engages in speculative interpretations that seem to rest on a very small number of occurrences or examples.

References:

The link/doi does not work: Oomen, M., & Roelofsen, F. (2023). Biased polar question forms in sign language of the netherlands (ngt): Two

functions of headshake. FEAST, 5, 156–168. https://doi.org/10.31009/FEAST.i5.13

Reviewer #2: Article Review- Polar questions in Dutch Sign Language (NGT)

Title: Polar questions in Dutch Sign Language (NGT): A production experiment

Authors: Marloes Oomen, Lyke Esselink, Floris Roelofsen

Journal: PLOS ONE (Manuscript draft PONE-D-25-55016)

Methodology: Controlled production experiment with native/early NGT signers

PONE-D-25-55016Review of Polar Questions in Dutch Sign Language (NGT): A Production Experiment

Overview and Contribution

Oomen, Esselink, and Roelofsen present a methodologically rigorous and theoretically significant study examining polar questions in Dutch Sign Language (NGT). Polar questions, also known as yes/no questions, are interrogatives that ask whether a proposition is true or false (e.g., Is Kim a vegetarian?), as opposed to content questions such as who, what, or where questions (Dryer, 2013). In linguistic terms, the polar questions examined in this study correspond to closed-ended questions, as they constrain responses to affirmation or negation. By contrast, open-ended (content) questions invite propositional elaboration and play a central role in Deaf-centered discourse practices, language pedagogy, and interpreter education. While polar questions have been extensively studied in spoken languages, particularly in relation to pragmatic bias, they remain comparatively underexplored in sign languages.

This study addresses that gap by investigating how Deaf signers of NGT produce polar questions across systematically varied pragmatic contexts. The authors make three significant contributions: (a) they document a rich inventory of polar question forms in NGT, including polarity-marked sentence radicals, multiple clause-final tags, and embedded interrogatives; (b) they demonstrate that these forms are systematically constrained by pragmatic context, specifically prior speaker belief and contextual evidence; and (c) they challenge long-standing assumptions that raised eyebrows constitute the primary or defining marker of polar questions in sign languages. Together, these findings advance both sign language linguistics and Deaf-centered linguistic description.

Theoretical Framing

The analysis is grounded in influential work on polar questions in spoken languages, which has shown that yes/no questions are not pragmatically neutral but are shaped by speaker expectations and evidential context (Büring & Gunlogson, 2000; Ladd, 1981). By adopting this framework, the authors position NGT as fully comparable to spoken languages in pragmatic sophistication rather than as a special or reduced case.

Crucially, the authors do not impose spoken-language categories wholesale onto NGT. Instead, they allow the grammatical resources of the language—manual structure, non-manual markers, and tag constructions—to emerge from production data. This approach respects the linguistic autonomy of signed languages while enabling meaningful cross-modal comparison.

Methodology

The study employs a controlled production experiment using role-play to elicit polar questions from six Deaf native or early NGT signers. Two contextual variables were manipulated: prior speaker belief (positive, neutral, negative) and contextual evidence (positive, neutral, negative). This design yielded 197 analyzable polar questions across seven experimental conditions.

Data collection and annotation are significant strengths of the study. Manual signs were glossed using established Signbank conventions (Crasborn et al., 2008), and non-manual markers were annotated across twelve ELAN tiers using a carefully developed annotation scheme. The authors’ decision to make all data, annotations, and materials publicly available reflects a strong commitment to transparency and replicability.

Key Findings

Diversity of Polar Question Forms

The authors identify a wide range of polar question constructions in NGT, including sentence radicals with positive, negative, or unmarked polarity, multiple clause-final tag constructions (including confirmation-type tags comparable to English right?), and embedded interrogatives. Each construction exhibits a distinct distribution across pragmatic contexts.

Pragmatic Bias and Contextual Sensitivity

A central contribution of the study is its demonstration that polar questions in NGT are systematically sensitive to pragmatic bias, paralleling well-established findings from spoken languages (Büring & Gunlogson, 2000; Ladd, 1981). Certain question forms presuppose positive speaker belief, others are favored when contextual evidence contradicts expectations, and some are infelicitous in neutral contexts.

Reconsidering Non-Manual Markers

Traditional descriptions of NGT and cross-linguistic surveys of sign languages have emphasized raised eyebrows as the primary marker of polar questions (Coerts, 1992; Zeshan, 2004). The present study challenges this view. Raised eyebrows are neither consistently present nor necessary in NGT polar questions. Instead, a forward head or body position emerges as the most reliable marker of polar interrogativity, while eyebrow configuration appears to encode pragmatic meanings such as expectation, doubt, or surprise.

Significance for Deaf Studies and Applied Contexts

Beyond its theoretical importance, this study is highly relevant to Deaf Studies, signed language pedagogy, and interpreter education. By demonstrating that polar questions are shaped by pragmatic reasoning rather than fixed facial rules, the authors support pedagogical approaches that treat non-manual markers as meaning-bearing and context-sensitive resources, aligning with Deaf-centered educational practice.

Conclusion

Polar Questions in Dutch Sign Language (NGT): A Production Experiment represents a major contribution to sign language linguistics. By explicitly demonstrating that yes/no (polar) questions in NGT are structurally diverse, pragmatically constrained, and sensitive to bias, the authors challenge long-standing assumptions and advance a more nuanced understanding of signed language grammar and use.

The study is theoretically impactful, methodologically sound, and highly relevant to Deaf-centered scholarship. It merits publication in a leading interdisciplinary journal and will serve as an important reference for future research on polar questions in sign languages.

References

Büring, D., & Gunlogson, C. (2000). Aren’t positive and negative polar questions the same? Journal of Semantics, 17(1), 1–38. https://doi.org/10.1093/jos/17.1.1

Coerts, J. (1992). Nonmanual grammatical markers: An analysis of interrogatives, negations, and topicalisations in Sign Language of the Netherlands. University of Amsterdam.

Crasborn, O., Zwitserlood, I., & Ros, J. (2008). The corpus NGT: A digital open access corpus of movies and annotations of sign language of the Netherlands. [http://hdl.handle.net/hdl:1839/00-0000-0000-0004-DF8E-6].

Dryer, M. S. (2013). Polar questions. In M. S. Dryer & M. Haspelmath (Eds.), The World Atlas of Language Structures Online. Max Planck Institute for Evolutionary Anthropology.

Ladd, D. R. (1981). A first look at the semantics and pragmatics of negative questions and tag questions. Chicago Linguistic Society, 17, 164–171.

Zeshan, U. (2004). Interrogative constructions in signed languages: Crosslinguistic perspectives. Language, 80(1), 7–39.

**********

6. PLOS authors have the option to publish the peer review history of their article (what does this mean?). If published, this will include your full peer review and any attached files.

If you choose “no”, your identity will remain anonymous but your review may still be made public.

**Do you want your identity to be public for this peer review?**  For information about this choice, including consent withdrawal, please see our Privacy Policy.

Reviewer #1: **Yes:** Anastasia A. Bauer

Reviewer #2: **Yes:** Beverly Buchanan

**********

[NOTE: If reviewer comments were submitted as an attachment file, they will be attached to this email and accessible via the submission site. Please log into your account, locate the manuscript record, and check for the action link "View Attachments". If this link does not appear, there are no attachment files.]

To ensure your figures meet our technical requirements, please review our figure guidelines: https://journals.plos.org/plosone/s/figures

You may also use PLOS’s free figure tool, NAAS, to help you prepare publication quality figures: https://journals.plos.org/plosone/s/figures#loc-tools-for-figure-preparation.

NAAS will assess whether your figures meet our technical requirements by comparing each figure against our figure specifications.

# Author comment: Author response to Decision Letter 1

**DOI:** 10.1371/journal.pone.0354015.r002

20 Apr 2026

Please see the Response to reviewers document for our response to each point raised by the editor and reviewers.

Attachment Submitted filename: Polar questions in NGT - Response to reviewers.pdf

# Editor report: Decision Letter 1

**DOI:** 10.1371/journal.pone.0354015.r003

**Authors:** Mary Diane Clark

2 Jul 2026

Polar questions in Dutch Sign Language (NGT): A production experiment

PONE-D-25-55016R1

Dear Dr. Oomen,

We’re pleased to inform you that your manuscript has been judged scientifically suitable for publication and will be formally accepted for publication once it meets all outstanding technical requirements.

Within one week, you’ll receive an e-mail detailing the required amendments. When these have been addressed, you’ll receive a formal acceptance letter and your manuscript will be scheduled for publication.

An invoice will be generated when your article is formally accepted. Please note, if your institution has a publishing partnership with PLOS and your article meets the relevant criteria, all or part of your publication costs will be covered. Please make sure your user information is up-to-date by logging into Editorial Manager at Editorial Manager® and clicking the ‘Update My Information' link at the top of the page. For questions related to billing, please contact billing support.

If your institution or institutions have a press office, please notify them about your upcoming paper to help maximize its impact. If they’ll be preparing press materials, please inform our press team as soon as possible -- no later than 48 hours after receiving the formal acceptance. Your manuscript will remain under strict press embargo until 2 pm Eastern Time on the date of publication. For more information, please contact onepress@plos.org.

Kind regards,

Mary Diane Clark, PhD

Academic Editor

PLOS One

Additional Editor Comments (optional):

Thank you for all of the work you put into this revision. I tried to get some additional reviewers but everyone is busy in the summer so I have carefully reviewed your paper and believe it is ready to be accepted. Some of the tables are wide but I am not sure how that will be addressed.

Reviewers' comments:

# Editor report: Acceptance letter

**DOI:** 10.1371/journal.pone.0354015.r004

**Authors:** Mary Diane Clark

PONE-D-25-55016R1

PLOS One

Dear Dr. Oomen,

I'm pleased to inform you that your manuscript has been deemed suitable for publication in PLOS One. Congratulations! Your manuscript is now being handed over to our production team.

At this stage, our production department will prepare your paper for publication. This includes ensuring the following:

* All references, tables, and figures are properly cited

* All relevant supporting information is included in the manuscript submission,

* There are no issues that prevent the paper from being properly typeset

You will receive further instructions from the production team, including instructions on how to review your proof when it is ready. Please keep in mind that we are working through a large volume of accepted articles, so please give us a few days to review your paper and let you know the next and final steps.

Lastly, if your institution or institutions have a press office, please let them know about your upcoming paper now to help maximize its impact. If they'll be preparing press materials, please inform our press team within the next 48 hours. Your manuscript will remain under strict press embargo until 2 pm Eastern Time on the date of publication. For more information, please contact onepress@plos.org.

You will receive an invoice from PLOS for your publication fee after your manuscript has reached the completed accept phase. If you receive an email requesting payment before acceptance or for any other service, this may be a phishing scheme. Learn how to identify phishing emails and protect your accounts at https://explore.plos.org/phishing.

If we can help with anything else, please email us at customercare@plos.org.

Thank you for submitting your work to PLOS ONE and supporting open access.

Kind regards,

PLOS ONE Editorial Office Staff

on behalf of

Dr. Mary Diane Clark

Academic Editor

PLOS One
